# Halochromic modulation of amorphous calcium carbonate crystallization driven by pH-responsive bioinspired pigments

**DOI:** 10.1038/s41467-026-75888-8

**Published:** 2026-08-03

**Authors:** Vaskar Sardhalia, Claudio Dos Reis Ferreira, Guillaume P. Laurent, Mohamed Selmane, Anne Vallée, Mathieu Frégnaux, Xiaoyan Li, Marta de Frutos, Andrew Fitch, Clemens N. Z. Schmitt, Shahrouz Amini, Zhaoyong Zou, Iryna Polishchuk, Boaz Pokroy, Peter Fratzl, Thierry Azaïs, Nadine Nassif, Marie Albéric

**Affiliations:** 1 Sorbonne Université, CNRS, Laboratoire Chimie de la Matière Condensée de Paris, LCMCP, Paris, France; 2 Sorbonne Université, CNRS, Fédération de Chimie et Matériau de Paris Centre, FCMat, Paris, France; 3 Sorbonne Université, CNRS, Laboratoire de Réactivité de Surface, LRS, Paris, France; 4 Université Paris-Saclay, UVSQ, CNRS, Institut Lavoisier de Versailles, Versailles, France; 5 Université Paris-Saclay, CNRS, Laboratoire de Physique des Solides, LPS, Orsay, France; 6 European Synchrotron Radiation Facility, Grenoble, France; 7 Max Planck Institute of Colloids and Interfaces, Biomaterials Department, Potsdam, Germany; 8 State Key Laboratory of Advanced Technology for Materials Synthesis and Processing, Wuhan University of Technology, Wuhan, China; 9 Technion Israël Institute of Technology, Department of Materials Science and Engineering, Haifa, Israël

**Keywords:** Biomineralization, Biomimetic synthesis, Organic-inorganic nanostructures

## Abstract

Colors in biominerals often arise from the occlusion of low-molecular-weight organic pigments within calcium carbonate (CaCO_3_) mineral phases during biomineralization processes; however, the physicochemical origin of these colors has been overlooked. Here, inspired by the growth of colored sea urchin spines that occurs through amorphous CaCO_3_ (ACC) precursors, we show an in-vitro halochromic modulation of ACC crystallization. In this mechanism, the deprotonation of a natural red pigment naphthazarin (NZ) is driven by pH variations during mineral formation and subsequently stabilized within CaCO_3_- hybrid pigments. The latter show lavender-blue to violet-blue hues upon crystallization. Deprotonated NZ has a minimal impact on ACC formation and crystallization, which occurs through local dissolution and reprecipitation mechanisms; however, it limits the formation of vaterite, and calcite distorted nanodomains. Deprotonated NZ nano-inclusions that interact with calcite through water are thus formed. Overall, this study brings insights into the processes behind the coloration of biomineralized organisms.

## Abstract (web summary)

It is unclear where biomineral colors, which arise from organic pigments trapped during mineral growth, originate. Here, the authors show that calcium carbonate crystallization determines the color of a bioinspired pigment sensitive to pH and involved in the mineralization process.

## Introduction

**Fig. 1:**
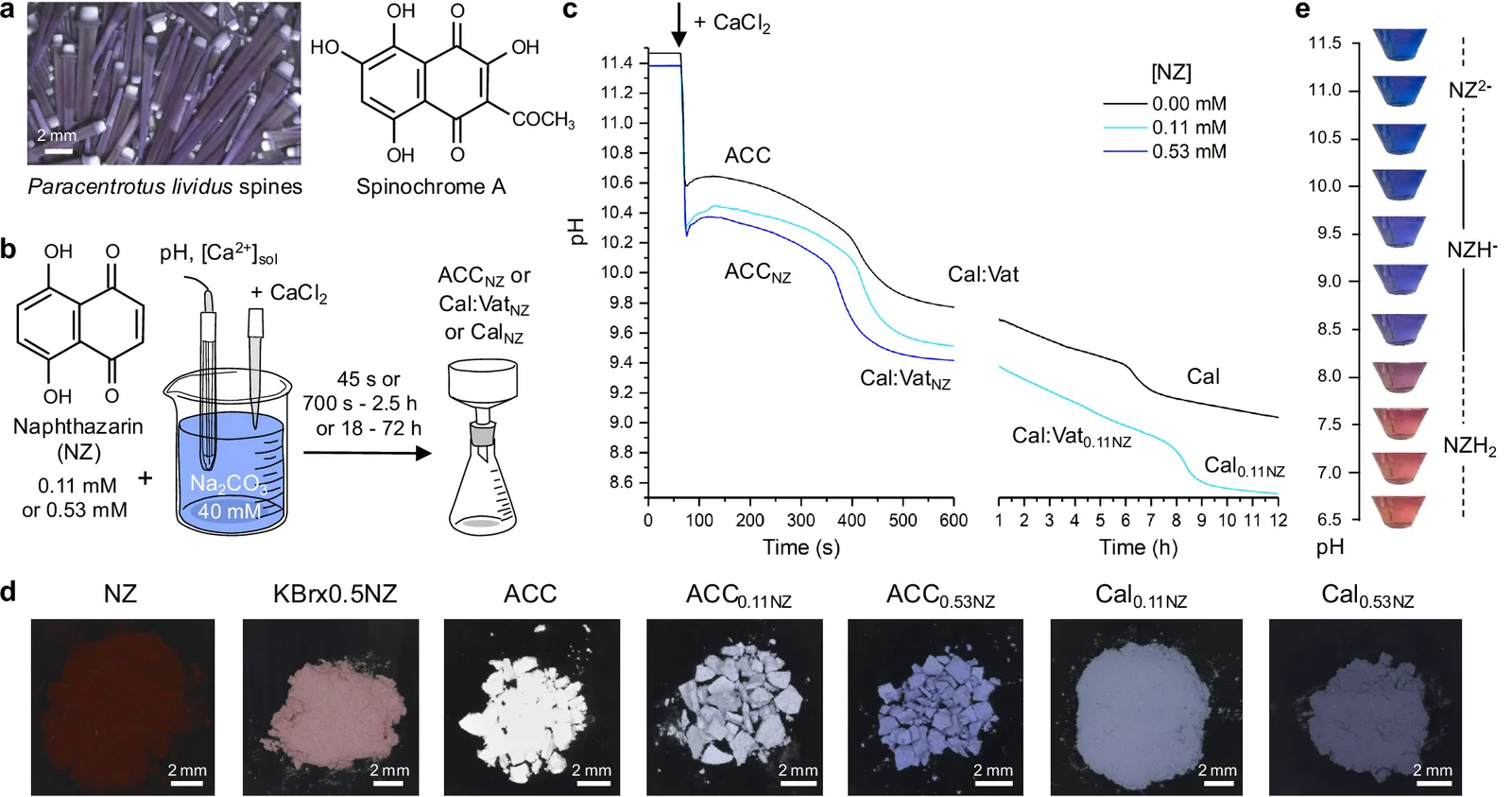
CaCO_3_ hybrid pigment synthesis. **a** Molecular structure of spinochrome A, the main PHNQ molecule of purple *Paracentrotus lividus* sea urchin spines, **b** schematic illustration of the direct precipitation of CaCO_3_ in the absence and in the presence of 0.11 and 0.53 mM NZ, **c** pH profiles as a function of time during ACC precipitation and crystallization into calcite (Cal) and vaterite (Vat) as well as vaterite conversion into calcite, in the presence of 0.11 or 0.53 mM [NZ] as compared to 0.00 mM [NZ], the addition of CaCl_2_ into the Na_2_CO_3_ solution, the plateau of ACC stability, its crystallization and the vaterite conversion are indicated, **d** stereo microscope images of pure NZ powder, KBrx0.5NZ powder obtained by physical mixing and ACC, ACC_0.11NZ_, ACC_0.53NZ_, Cal_0.11NZ_ and Cal_0.53NZ_ powders synthesized by the direct precipitation method and **e** color changes according to pH of a 0.11 mM NZ solution with the three NZ forms displayed: NZ^2-^, NZH^-^ and NZH_2_.

Marine life is known for its ability to produce a large variety of multifunctional biomineralized structures that are often colorful. The wide range of hues observed in calcium carbonate (CaCO_3_)-based biominerals can be attributed to physical and/or chemical factors^1,2^. Among these, is the occlusion of low-molecular-weight organic pigment molecules ( < 1 kg mol^−1^), such as carotenoids, bilins, porphyrins, melanin, and naphthoquinones, within the skeletal elements of marine invertebrates, including mollusks, brachiopods, crustaceans, red corals, crinoids, and sea urchins^2,3^. Of particular interest are the polyhydroxy-1,4-naphthoquinone pigments (PHNQs)^4^, which are biosynthesized by sea urchins through enzymatic pathways^5^. About 40 PHNQs have been discovered so far, such as spinochrome A (3-acetyl-2,5,7,8-tetrahydroxy-1,4-naphthoquinone), e.g., extracted from purple spines of *Paracentrotus lividus* sea urchins (Fig. 1a)^1^, and echinochrome A (7-ethyl-2,3,5,6,8-pentahydroxy-1,4-naphthoquinone), which exhibits UV-absorption, antioxidant, and antibacterial properties^6–8^. These bio-active secondary metabolites are also responsible for the multiple colors observed in sea urchins, from red in the immune red-spherule cells to yellow, pink, red, purple, and green once encapsulated within the CaCO_3_ biominerals^1,6,9^, at a level of ≈ 0.1 wt.%^10^. The intense colors displayed by PHNQs result from significant electron delocalization within the quinoid structure^11^ and vary in solution according to pH due to successive deprotonations of the numerous hydroxyl groups^10,12–14^. Hence, the local pH at the site of mineralization in sea urchins^15^ may dictate the final color of the biominerals by tuning the protonation state of PHNQs during their encapsulation within the mineral phase. Sea urchins are therefore producing biogenic hybrid pigments, whose colors resist chemical treatments^16^ due to the protection provided by the mineral phase of the otherwise unstable organic pigments, a mechanism well-known from the ancient Maya blue pigment^17^.

Although little is known about the biological mechanisms of organic pigment incorporation into biominerals, biomineralization pathways, i.e., the formation of minerals by organisms, have been largely studied^18,19^. In sea urchins^20,21^, as in other marine organisms^22,23^, they were shown to occur through the deposition of metastable amorphous calcium carbonate (ACC) nanoparticles that inorganic and organic additives can temporarily stabilize before crystallizing into calcite or aragonite^24–32^. In this regard, the role of organic molecules in the composition, structure, stabilization, and crystallization mechanisms of biogenic and synthetic ACCs has been extensively investigated^24–32^. In addition, calcite crystals have been previously colored with synthetic sulfonated dyes that exhibit preferential interactions with specific calcite planes^33–35^. However, the role of natural organic pigments, such as secondary metabolites, on ACC formation and crystallization mechanisms into calcite, and vice versa, remains unexplored.

Here, we investigate the impact of naphthazarin on ACC formation and crystallization, thereby elucidating the origin of color changes during these transformations and investigating the incorporation of this dye within the various CaCO_3_ polymorphs classically formed. We chose naphthazarin (NZ, 5,8-dihydroxy-1,4-naphthoquinone), a secondary metabolite found in plants^36^, because it has an analogous structure to PHNQs and is commercially available, unlike PHNQs. In addition, NZ exhibits similar halochromic properties to those of spinochrome A, which changes from red to purple to blue with increasing pH^10,37^. We thus produced colored amorphous and crystalline CaCO_3_ hybrid pigments with different shades of blue from lavender to violet, using a red organic pigment. Hence, we demonstrate the halochromic modulation of ACC crystallization pathways, in which NZ modifies the kinetic and thermodynamic formation of the crystalline CaCO_3_ polymorphs while being occluded in biologically relevant concentrations.

## Results

### Influence of NZ on ACC formation and crystallization

The direct precipitation of CaCO_3_ in the presence of NZ was performed by the fast and automated addition of CaCl_2_ into 40 mM Na_2_CO_3_ solutions^38,39^ with [NZ] = 0.11 or 0.53 mM, using a Metrohm® Titrando setup (Fig. 1b). This setup enables monitoring the pH and free [Ca^2+^] in solution during the different steps of the reaction (see Methods). A pH plateau is observed until 400-450 s, which corresponds to the time period when most of the ACC phase is stable (Fig. 1c). Afterward, a first pH decrease is measured as well as a second one after several hours, which respectively correspond to the crystallization of ACC into a mixture of calcite and vaterite and the conversion of the transient vaterite into calcite. The formation of the different CaCO_3_ polymorphs, typically observed in these alkaline pH conditions^27,39^, is confirmed by laboratory X-rays diffraction (XRD) and high-resolution XRD (HR-XRD) measurements (Supplementary Fig. 1). In this study, ACC nanoparticles, calcite: vaterite (Cal:Vat), and calcite (Cal) crystals are respectively recovered at 45 s, 700 s, or 2.5 h and 18-72 h after CaCl_2_ addition and filtration of the precipitate (Fig. 1b). The short names corresponding to those different samples are detailed in Table 1.

**Table 1:** List of samples

Sample names	Description
ACC	ACC powder obtained by direct precipitation at 45 s after CaCl_2_ addition into the Na_2_CO_3_ solution
ACC_0.11NZ_	ACC obtained in the presence of 0.11 mM NZ in the Na_2_CO_3_ solution
ACC_0.53NZ_	ACC obtained in the presence of 0.53 mM NZ in the Na_2_CO_3_ solution
Cal:Vat	Mixture of calcite and vaterite powder obtained by direct precipitation at 700 s or 2.5 h after CaCl_2_ addition into the Na_2_CO_3_ solution
Cal:Vat_0.11NZ_	Cal:Vat obtained in the presence of 0.11 mM NZ in the Na_2_CO_3_ solution
Cal:Vat_0.53NZ_	Cal:Vat obtained in the presence of 0.53 mM NZ in the Na_2_CO_3_ solution
Cal	Calcite powder obtained by direct precipitation at 18, 24, 48 or 72 h after CaCl_2_ addition into the Na_2_CO_3_ solution
Cal_0.11NZ_	Cal obtained in the presence of 0.11 mM NZ in the Na_2_CO_3_ solution
Cal_0.53NZ_	Cal obtained in the presence of 0.53 mM NZ in the Na_2_CO_3_ solution
Calx0.5NZ	Physical mixture of Cal obtained at 48 h and NZ at 0.5 wt.%
KBrx0.5NZ	Physical mixture of KBr powder and NZ at 0.5 wt.%
NZH_2_ powder	Protonated NZ powder
NZH^-^ powder	Singly deprotonated NZ powder
NZ^2-^ powder	Doubly deprotonated NZ powder

Although NZ appears to slightly lower the pH at which ACC precipitates, it does not significantly influence its formation. Indeed, neither the size of the ACC nanoparticles (140 ± 40 nm), the ACC local structure (Fig. 1, Supplementary Fig. 2), nor its stability against crystallization in solution is significantly modified by the presence of NZ. For [NZ] ≤ 0.11 mM, ACC crystallization is delayed by ≈ 50 s whereas it is accelerated by ≈ 50 s for [NZ] > 0.11 mM (Fig. 1c). In contrast, NZ increases the Cal:Vat percentage from 59:41 to 65:35 wt.% immediately after ACC crystallization (700 s) (Supplementary Fig. 1c, d), therefore promoting the formation of calcite over vaterite. In addition, NZ largely delays the conversion of the transient vaterite phase into calcite by 2 h for [NZ] = 0.11 mM (Fig. 1c) and up to 72 h for [NZ] = 0.53 mM (Supplementary Fig. 1e, f).

The direct precipitation of CaCO_3_ in the presence of NZ results in the formation of colored CaCO_3_ powders with various shades of blue, while pure ACC powder is white and pure NZ, dark red (Fig. 1d, Supplementary Fig. 3). In addition, NZ physically mixed with KBr powder in different proportions shows light red shades (Fig. 1d, Supplementary Fig. 3). We hypothesize that the various shades of blue of the synthesized hybrid pigments likely originate from the halochromic properties of NZ in solution, which changes from red at acid pH to purple and then to blue at basic pH^37^ (Fig. 1e). These color differences are caused by successive deprotonations of the OH groups, resulting in three NZ forms: fully protonated (NZH_2_), singly deprotonated (NZH^-^), or doubly deprotonated (NZ^2-^). We therefore further investigate the origin of these blue shades by determining the protonation state of NZ in solution as well as within the solid hybrid pigments during ACC formation and crystallization into calcite.

### Color changes during ACC formation and crystallization

**Fig. 2:**
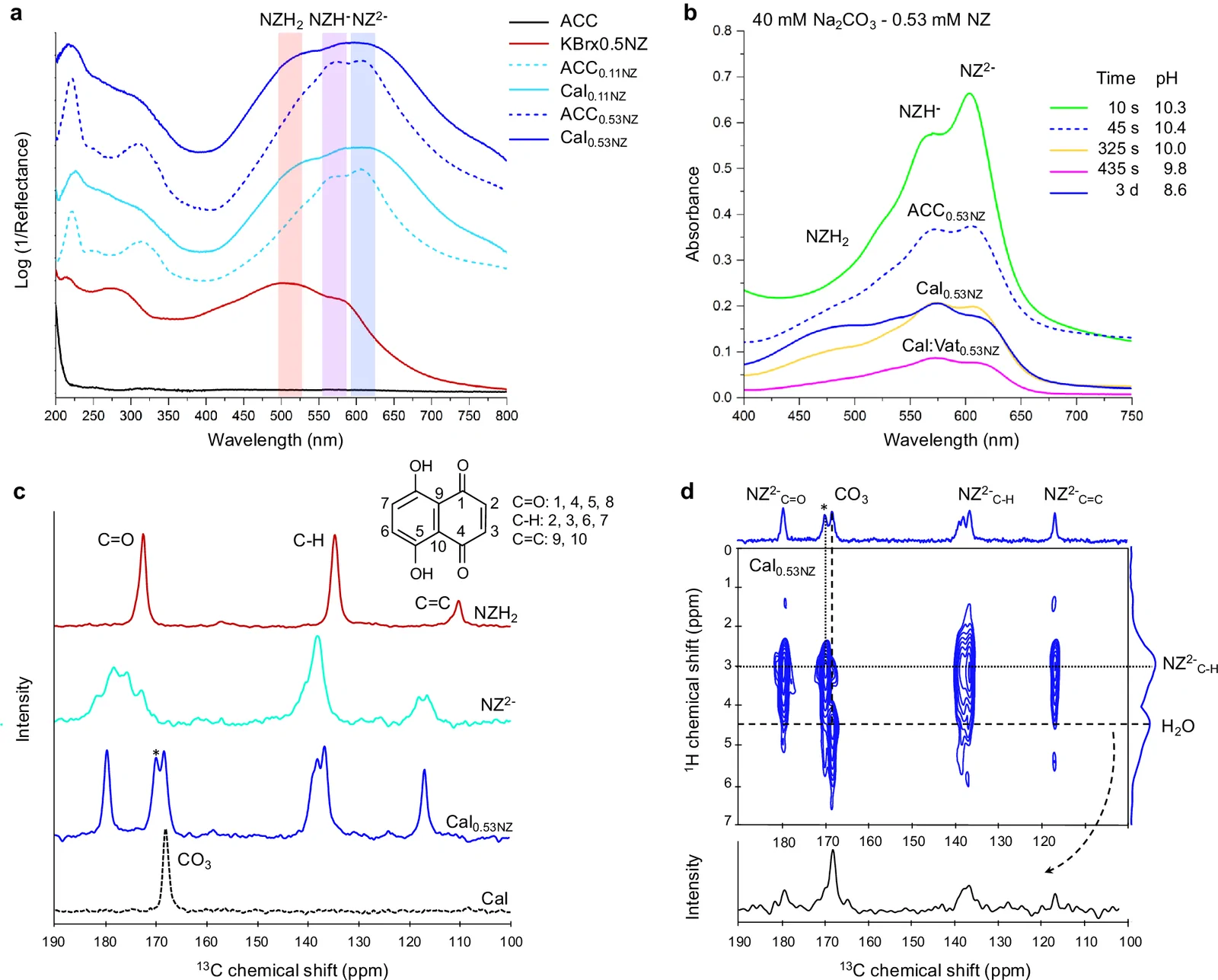
NZ protonation states during ACC formation and crystallization. **a** UV-Vis DRS spectra of ACC (in solid black), KBrx0.5NZ (in solid red), ACC_0.11NZ_ (in dashed cyan), Cal_0.11NZ_ (in solid cyan), ACC_0.53NZ_ (in dashed blue) and Cal_0.53NZ_ (in solid blue), the colored vertical bars indicate the absorption maxima of the NZ^2-^ (in light red), NZH^-^ (in light purple) and NZH_2_ (in light blue) forms, **b** quantitative absorbance measurements of the reaction medium taken at 10 s (in green), 45 s (in dashed blue), 325 s (in yellow), 435 s (in magenta), and 3 days (in solid blue) after CaCl_2_ addition, the corresponding pH and sample names at the different time points of the reaction are indicated, absorption maxima of the NZ^2-^, NZH^-^ and NZH_2_ forms are also annotated on top of the UV-Vis spectra, **c** ^13^C CPMAS NMR spectra of NZH_2_ powder (in red), NZ^2-^ powder prepared at pH 12 (in turquoise), Cal_0.53NZ_ (in bleu) and Cal (in dashed black) and **d** 2D ^1^H-^13^C HETCOR NMR spectrum of Cal_0.53NZ_ (contour map, ^1^H and ^13^C spectra in blue), the asterisk (*) indicates the 170 ppm peak, which correlates with the C-H resonance of NZ^2-^ (dotted black lines), the correlation between the CO_3_ resonance from calcite and the ^1^H resonance from H_2_O is displayed by dashed black lines, the ^13^C slice (black spectra) extracted from the ^1^H resonance is also shown and, the C = O, C-H, and C = C resonances of NZ, and the CO_3_ resonance of calcite, indicated.

The different colors of the samples were quantified by UV-Vis diffuse-reflectance spectroscopy (DRS) measurements (Fig. 2a, and Supplementary Fig. 3a, b), which allow for the calculation of the L*, a*, b* parameters as well as the sRGB values of the different samples (Supplementary Table 1, see “Methods”) and finally, the assignment of the lavender-blue and violet-blue colors of the ACC_NZ_ and Cal_NZ_ samples, respectively. The UV-Vis DRS spectra also confirm the presence of NZ in the hybrid pigments, which display spectra similar to those of NZ. In contrast, the spectra of pure ACC and Cal display no characteristic absorbance bands (Fig. 2a). We note that the DRS spectra of Cal_NZ_ samples show broader bands than those of ACC_NZ_, likely due to the larger size of calcite crystals compared to ACC nanoparticles. In more detail, NZ mixed with KBr (KBrx0.5NZ) to reduce its strong absorption, shows bands centered at 212, 270, 420, and 500 nm (Fig. 2a, and Supplementary Fig. 3a). These bands are very similar to those previously reported for NZ solutions. They are all attributed to several *π*→π* transitions induced by the planar conformation of NZ^37,40–42^ that give its red color to this dye. The colored CaCO_3_ samples also display similar visible bands, but are all red-shifted by ≈ 80 nm as compared to NZ, thus explaining the blue colors of the powders (Fig. 1b). These bands at 530, 570, and 606 nm display different relative intensities depending on [NZ], suggesting different protonation states of NZ and, consequently, various hues, as explained in detail below. Furthermore, the two UV bands at 220 and 310 nm, attributed to electron transfer in the quinoid moiety of NZ^42^, are also red-shifted, revealing some particular inorganic-organic interactions within the synthesized hybrid pigments.

As mentioned above, pH and free [Ca^2+^] vary during the formation and crystallization of ACC (Fig. 1c). For [NZ] = 0.53 mM, ACC_0.53NZ_ forms at pH 10.4 and free [Ca^2+^] = 0.5 mM; crystallization into calcite and vaterite occurs at pH 9.8; and the conversion of vaterite into calcite happens at pH 8.6 and free [Ca^2+^] < 0.1 mM (Supplementary Fig. 4a). To relate these pH variations to the speciation of NZ in solution, its two pK_a_ values were calculated based on UV-vis spectroscopy measurements under the conditions of ACC formation, i.e., with solutions of 40 mM Na_2_CO_3_ containing 0.53 mM NZ (Supplementary Fig. 4b and Supplementary Note 1). The calculated pK_a1_ is 8.2, and pK_a2_ is 10.3 for these specific conditions. These values fall within the reported ranges of 6-9.3 for pK_a1_ and 10.4-11.8 for pK_a2_, depending on the solvent used^37,40,41^. Because the presence of Ca^2+^ in solution is known to shift pK_a_ values of PHNQs and promote their oxidation at basic pH^12^, the effect of Ca^2+^ on NZ protonation states and oxidation was also studied (Supplementary Fig. 4d–f and Supplementary Note 1). UV-Vis spectroscopy measurements show no significant oxidation of NZ, but the pK_a_ values decrease by one unit in the presence of 2 mM Ca^2+^. The latter suggests the formation of Ca^2+^-NZ complexes, as previously observed for several PHNQ molecules^8,14^.

The speciation of NZ can be further quantitively linked to the different colors of the solutions, visible to the naked eye, by calculating the *x* and *y* values of the commission internationale de l’éclairage (CIE) color space based on the visible spectra and plotting them on the CIE diagram (Supplementary Fig. 4c, see “Methods”). The calculated colors, according to pH, follow a linear trend from blue to red likely due to the relative intensities of the 515, 575, and 615 nm absorbance bands, respectively assigned to the NZH_2_, NZH^-^, and NZ^2-^ forms.

The protonation states of NZ, and consequently the color changes of the solution during CaCO_3_ formation, were further determined through UV-vis spectroscopy measurements of the supernatants at various key time points of the reaction (Fig. 2b). All spectra exhibit the characteristic absorbance bands of NZH_2_, NZH^-,^ and NZ^2-^ with different relative intensities. The spectrum of the solution 10 s after CaCl_2_ addition displays a more intense 615 nm band than the 575 nm one, revealing the predominant presence of the NZ^2-^ form even though pH=pK_a2_. Indeed, at this time point, the presence of [Ca^2+^] = 1.2 mM likely decreases the pK_a_ value. This also suggests the formation of Ca^2+^-NZ complexes in solution and, therefore, some competition between NZ^2-^ and CO_3_^2-^ for binding to Ca^2+^ ions. About 45 s after CaCl_2_ addition, when ACC is recovered, the 615 and 575 nm bands are of equal intensity, indicating the presence of both NZ^2-^ and NZH^-^ forms at pH≈pK_a2_ and free [Ca^2+^] = 0.5 mM. The effect of Ca^2+^ on the pK_a_ decrease is negligible here, suggesting the disappearance of the Ca^2+^-NZ complexes in solution. During ACC crystallization, the proportion of the NZH^-^ form increases until it becomes higher than the NZ^2-^ one at pH 9.8, after complete crystallization into a mixture of calcite and vaterite. At pH 8.6, after the conversion of vaterite into calcite, the NZH^-^ form is the main one.

Therefore, pH fluctuations during ACC formation and subsequent crystallization appear to regulate NZ speciation in solution, which directly influences the color of the reaction medium, ranging from blue to purple.

To further link these color changes in solution to those of the synthesized solid hybrid pigments, we investigated the NZ protonation states of the powder samples. Similarly to in solution, they can, in principle, be assessed based on the relative intensity of the absorption bands measured by DRS (Fig. 2a). For ACC_0.53NZ_, equal intensity of the NZ^2-^ and NZH^-^ bands is measured, indicating that these two forms are equally present in the solid sample, similarly to the ones in solution. For ACC_0.11NZ_, the NZ^2-^ form appears to be more present than the NZH^-^ one. This difference in NZ protonation state according to [NZ] is in agreement with the pH at which both ACC samples are synthesized (Fig. 1c).

The protonation state of NZ of the Cal_NZ_ samples is, however, difficult to determine based on their UV-Vis spectra because they display broad bands (Fig. 2a). It was therefore investigated through ^13^C nuclear magnetic resonance (NMR) solid-state spectroscopy by measuring Cal_0.53NZ_ and pristine NZ in its three different protonated states: NZH_2_, NZH^-^_,_ and NZ^2-^ (Fig. 2c, and Supplementary Fig. 5a, b) (see “Methods”). The ^13^C spectra of pristine NZ samples display three main resonances assigned to the different carbon groups: carbonyl carbons C = O (1, 4, 5, 8), protonated carbons =C-H (2, 3, 6, 7), and quaternary carbons C = C (9, 10)^43^. Upon deprotonation, the position of these ^13^C resonances shifts toward higher chemical shifts (*δ*), from 173 to 180 ppm for C = O, 135 to 138 ppm for =C-H, and 110 to 117 ppm for C = C (Fig. 2c). This indicates that the ^13^C signal positions are a reliable indicator of NZ protonation states. This is further confirmed by liquid-state NMR measurements, which, in addition, evidenced ^1^H peak shifts toward lower ppm values upon deprotonation (Supplementary Fig. 5c, d). Moreover, two-dimensional (2D) ^1^H-^13^C heteronuclear correlation (HETCOR) NMR experiments showed to be a good proxy of NZ protonation states as well. Indeed, the OH groups are clearly detected (*δ*(^1^H) ≈ 13 ppm) for the NZH_2_ form, while they are absent for the NZ^2-^ form (Supplementary Fig. 5a, b).

Comparison of these NZ ^13^C spectra with that of Cal_0.53NZ_ reveals similar positions of the three ^13^C resonances from NZ at 180, 137, and 117 ppm to those of the NZ^2-^ form (Fig. 2c). This indicates that NZ is deprotonated when associated with calcite. However, the linewidth of the resonances differs, suggesting a specific environment induced by the presence of calcite, which is identified by the ^13^C resonance at 168 ppm, characteristic of carbonates. The additional ^13^C signal (indicated by a star) detected at 170 ppm is also assigned to NZ because it correlates with the ^1^H resonance from the C-H groups at 3.1 ppm (Fig. 2d, dotted black lines). Although it is difficult to conclude about the protonation state corresponding to this signal, the lack of specific ^1^H resonance from OH groups around 12-14 ppm (as compared to the 2D ^1^H-^13^C HETCOR of NZH_2_; Supplementary Fig. 5d) confirms the deprotonated form of NZ molecules associated with calcite in Cal_0.53NZ_.

Overall, the combination of UV-Vis spectroscopy and solid-state ^13^C and ^1^H NMR measurements demonstrates that both ACC_NZ_ and Cal_NZ_ samples contain deprotonated NZ forms, which contribute to their different shades of blue from lavender to violet.

### Interactions of NZ molecules with CaCO_3_

The 2D ^1^H-^13^C HETCOR spectrum also allows investigating NZ, water, and calcite interactions by probing spatial proximities at the molecular scale (Fig. 2d). The correlation measured between the CO_3_ resonance and the ^1^H resonance at 4.6 ppm (Fig. 2d, dashed black lines) attributed to a local proton environment, presumably adsorbed and trapped water, reveals that water molecules are associated with calcite. In addition, the ^13^C slice (Fig. 2d, black spectra) extracted from the ^1^H resonance from water molecules shows that the latter shares a certain proximity to NZ molecules. In contrast, no direct interaction between calcite and NZ is measured. Therefore, we propose that the interaction between calcite and NZ molecules is mediated by the presence of water molecules, as similarly evidenced by solid-state NMR in other systems^44^.

**Fig. 3:**
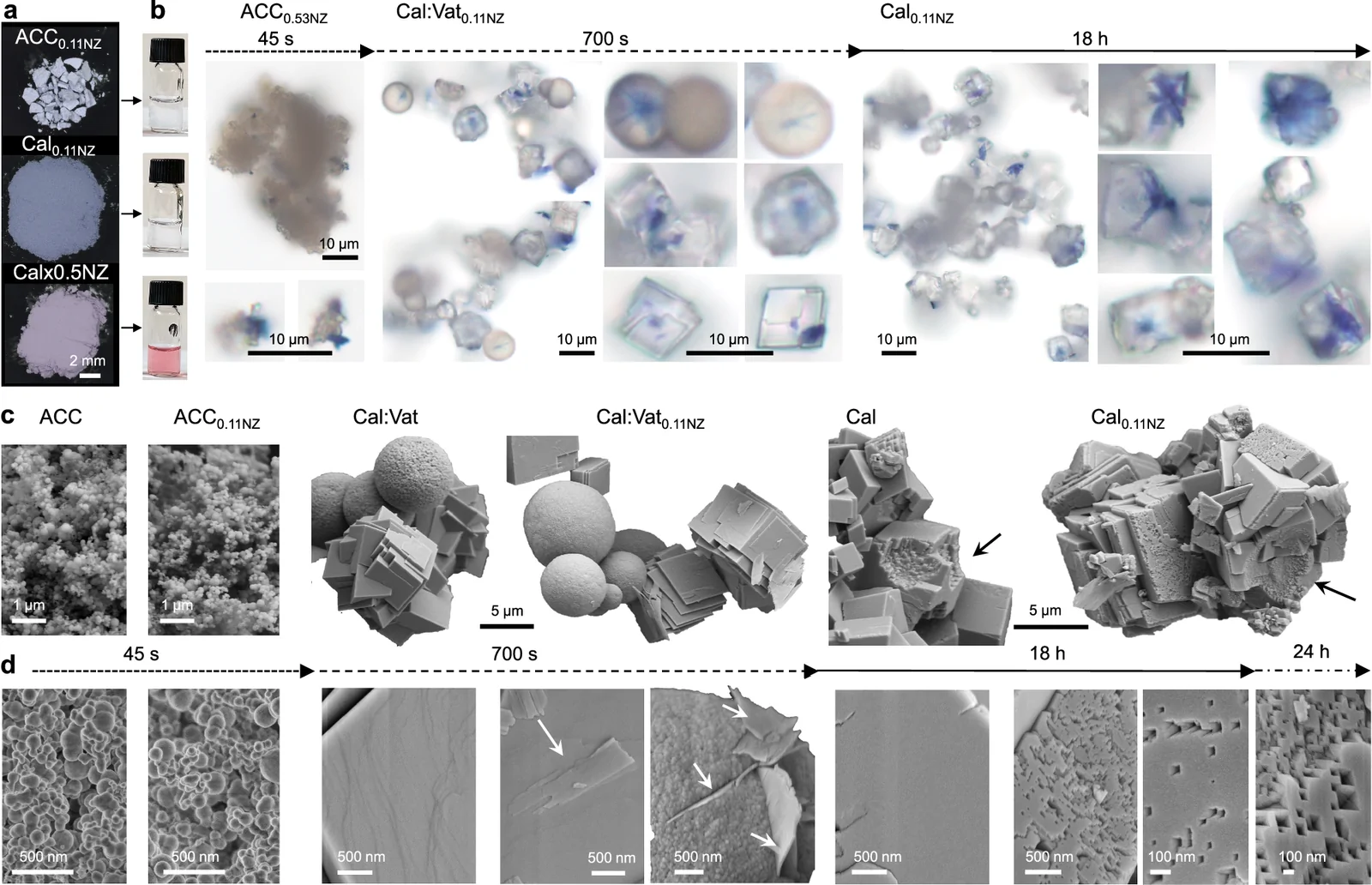
NZ-CaCO_3_ interactions from the macro- to the micro-scale. **a** from top to bottom, colors of the powders and the ethanol solutions obtained after suspension of ACC_0.11NZ_, Cal_0.11NZ_ and Calx0.5NZ, **b** transmission optical microscope images of ACC_0.53NZ_ nanoparticles at 45 s, Cal:Vat_0.11NZ_ crystals at 700 s as well as Cal_0.11NZ_ crystals at 18 h, **c** low- and **d** high-resolution FEG-SEM micrographs of the ACC and ACC_0.11NZ_ at 45 s; Cal:Vat and Cal:Vat_0.11NZ_ at 700 s as well as Cal and Cal_0.11NZ_ at 18 h and 24 h, the white arrows point to NZ crystals and the dark arrows to pseudospherical casts.

The occlusion of NZ within the ACC_0.11NZ_ and Cal_0.11NZ_ samples was assessed by suspending them in ethanol for 2 h (Fig. 3a). To serve as a control, a pink-purple powder obtained by physical mixing of pure calcite with NZ (called Calx0.5NZ) was also suspended in ethanol. After suspension, the solutions obtained from the precipitated samples are colorless, whereas the one resulting from the physically mixed powder is light red. This demonstrates the strong electrostatic adsorption of NZ molecules onto the surface of ACC_0.11NZ_ and Cal_0.11NZ_, as well as their potential encapsulation, in contrast to their weak adsorption on the crystals of Calx0.5NZ, which is obtained by physical mixing.

The presence of NZ molecules at the surface versus within the bulk of the precipitated samples was further quantified by X-ray photoelectron spectroscopy (XPS) measurements (Supplementary Fig. 6) and UV-vis spectroscopy (Supplementary Table 2), respectively.

In agreement with the literature, the XPS C 1 *s* spectrum of ACC and Cal shows, respectively, a carbonate peak at 289.5 ± 0.1 eV^28^ and 289.2 ± 0.1 eV^45^. In contrast, the spectrum of ACC_0.53NZ_ and Cal_0.53NZ_ displays additional peaks, attributed to the C = O, C-OH and C *sp*^2^ bonds of NZ (Supplementary Fig. 6). Fitting these spectra allows for the calculation of surface atomic percentage of organic C of 31% for ACC, 54% for ACC_0.53NZ_, 38% for Cal and 69% for Cal_0.53NZ_ (see *C*_organic_/*C*_inorganic+organic_, Supplementary Table 2). This indicates that calcite crystals present more adsorbed NZ at their surface than ACC nanoparticles.

The total amount of NZ associated with the ACC_NZ_ and Cal_NZ_ samples was determined by dissolving the powders with HCl and calculating the different [NZ] concentrations released in solution based on spectroscopy measurements (see “Methods”). The results summarized in Supplementary Table 2, show that ACC_0.11NZ_ contains 0.51 wt.% NZ, ACC_0.53NZ_ holds 2.40 wt.% NZ, Cal_0.11NZ_, 0.47 wt.% NZ and Cal_0.53NZ_, 2.26 wt.% NZ. This indicates that all samples capture most of the NZ molecules initially dissolved in the Na_2_CO_3_ solution, confirming significant interactions between NZ and CaCO_3_.

The occlusion of NZ within the calcite crystals was further quantified by subjecting the Cal_NZ_ powders to 1% NaClO treatment to remove adsorbed NZ molecules prior to dissolution and visible spectroscopy measurements. Similar to sea urchin spines (0.1 wt.%^10^), only 0.05 wt.% and 0.15 wt.% NZ are occluded within the Cal_0.11NZ_ and Cal_0.53NZ_ samples, respectively, representing 8% and 6% of the total NZ amount (Supplementary Table 2). The incorporation of NZ within the ACC samples could not be evidenced using this approach due to the dissolution of ACC in the NaClO solution, which resulted in the further degradation of the released NZ molecules. Nevertheless, because Cal_NZ_ and ACC_NZ_ comprise a similar amount of total NZ and ACC_NZ_ shows less adsorbed NZ than Cal_NZ_ (XPS results), the former must encapsulate more than the latter. These results therefore suggest that ACC crystallization into calcite likely occurs through dissolution/reprecipitation mechanisms where NZ is released during ACC dissolution and reincorporated during calcite precipitation, but in smaller amounts.

Despite the intense and homogeneous shades of blue perceived at the macroscale, optical microscopy observations reveal heterogeneous distributions of the coloration within the CaCO_3_ samples (Fig. 3b). Both pure ACC and ACC_NZ_ samples, which consist of aggregated spherical nanoparticles, appear orange. This orange hue, attributed to a structural color, likely originates from the specific size of the aggregated nanoparticles^46^ and might prevent the visualization of possible lavender blue pigmentation of the nanoparticles. Nevertheless, blue stars and flakes are observed at the surface of the ACC nanoparticles (Fig. 3b). They appear to be crystallized NZ molecules, which are better observed under field emission gun scanning electron microscopy (FEG-SEM) (Supplementary Fig. 7a). While blue crystallized NZ is also observed on the surface of some Cal_NZ_ crystals, additional NZ molecules appear to be occluded within several calcite crystals (Fig. 3b), which then exhibit internal diffuse violet and blue colorations even when changing the focal plane (Supplementary Fig. 7b, d). In most cases, the blue shade is concentrated in the middle of the Cal_NZ_ crystals while pure Cal ones are transparent (Supplementary Fig. 7e).

As seen by optical microscopy (Fig. 3b), FEG-SEM observations reveal characteristic rhombohedral calcite and spherical vaterite crystals, regardless of the presence of NZ (Fig. 3c). In addition, vaterite spheres show nano-crystalline spherical sub-units of about 50-80 nm diameter, indicating the partial dissolution of some ACC nanoparticles ( > 80 nm)^47^ and their subsequent aggregation and crystallization into vaterite^27^. After vaterite conversion, some calcite crystals exhibit pseudospherical casts, suggested to be the result of a dissolution/reprecipitation mechanism of vaterite into calcite^48^ (Fig. 3c, black arrows).

High-resolution FEG-SEM images allow for visualizing the potential effect of NZ interactions with the growing crystals (Fig. 3d, and Supplementary Fig 8a–d). After ACC crystallization, the surface of the crystals is not affected by the presence of NZ, which form blue flakes or stars on the calcite surface or in between the spherical sub-units of vaterite, as already reported for Brilliant Blue R dye^34^. After vaterite conversion, however, the presence of NZ significantly modifies the surface of calcite crystals (Fig. 3d, and Supplementary Fig. 8a). They exhibit crevasses and numerous 20–50 nm rhombohedral holes that increase in size up to 200 nm after two to three days of synthesis (Fig. 3d, and Supplementary Fig. 8b, c). Similar crevasses were observed in the presence of large polymers^49^ proposed to disturb crystal growth due to nonspecific interactions^49,50^ and induce the dissolution of the calcite surface^49^. Although individual NZ molecules are small (about 6 Å in size), they are reported to form larger molecular packing motifs^51^ and chelate complexes with several metallic ions^52,53^. We therefore suggest that stacked NZ molecules could also generate such crevasses at the surface of the calcite crystals. In parallel, the rhombohedral holes resemble the etch pits obtained during calcite dissolution in acidic aqueous solution^54^ and up to pH 8.6^55^, indicating that NZ could also indirectly induce these pits by lowering the pH of the synthesis medium from 9.2 to 8.6, as measured here (Fig. 1c).

Moreover, numerous ellipsoid nanopores with a broad size distribution ranging from 20 to 250 nm were observed within a focused ion beam (FIB) lamella of the Cal_0.53NZ_ sample (Supplementary Fig. 8f, g). They resemble the internal inclusions filled with negatively charged polymers in synthetic calcite^56^ or with highly acidic proteins in mollusk shells^57^. We note that round or rhombohedral internal pores were also obtained, depending on the charge of the organic molecules considered^58^, revealing the importance of charges in the formation of such composite material, as discussed below in the case of NZ, considering its protonation state.

**Fig. 4:**
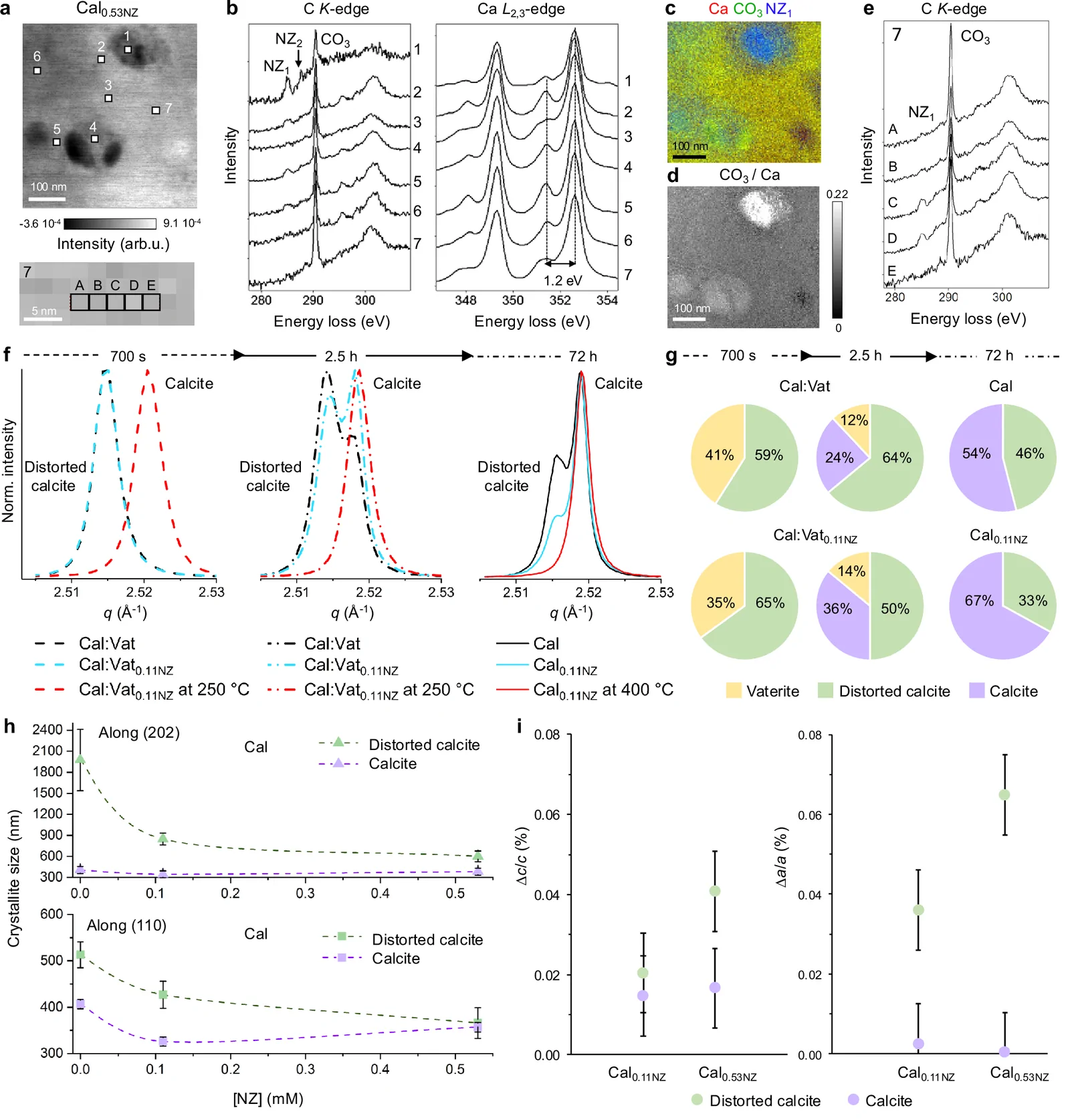
NZ-CaCO_3_ interactions at the nanoscale. **a** HAADF-STEM image of a representative area of the FIB lamella from the Cal_0.53NZ_ sample, **b** C *K*- and Ca *L*_2,3_-edges spectra integrated over seven key areas (40 × 40 nm^2^) labeled on a, **c** RGB composite image combining the maps of Ca (in red), CO_3_ (in green) and NZ_1_ (in blue), for the grayscale of each channel see Supplementary Fig. 9b and **d** CO_3_/Ca map of the representative area displayed in a, **e** C *K*-edge spectra integrated over one pixel (4 × 4 nm^2^) of area 7 labeled in a, **f** (110) calcite peaks of the HR-XRD profiles of Cal:Vat (in black), Cal:Vat_0.11NZ_ (in cyan) at 700 s (in dashed lines) and 2.5 h (in dashdotted line) before and after annealing at 250 °C (in red) and Cal (in black), Cal_0.11NZ_ (in cyan) at 72 h (in solid lines) before and after annealing at 400 °C (in red), **g** proportions of vaterite (in yellow), distorted calcite (in light green) and calcite (in light purple) in Cal:Vat, Cal:Vat_0.11NZ_, Cal and Cal_0.11NZ_ samples, **h** crystallite size (nm) of the distorted calcite (in light green) and calcite (in light purple) nanodomains along the (202) and (110) directions and **i** calcite lattice distortions along the *c*-axis (∆*c*/*c*) and (*ab*) plane (∆*a*/*a*) of the distorted calcite (in light green) and calcite (in light purple) nanodomains of Cal_0.11NZ_ and Cal_0.53NZ_ with respect to Cal. The error bars correspond to the standard error of the fits, respectively with the Voigt function and Rietveld refinement.

The spatial distribution of occluded NZ molecules within the calcite crystals was then studied by analyzing the FIB lamella of the Cal_0.53NZ_ sample by high-angle annular dark-field (HAADF)–scanning transmission electron microscopy (STEM) coupled with Electron Energy Loss Spectroscopy (EELS) (Fig. 4a–d). Several EELS data sets were acquired using the spectrum-imaging mode, which allows for the subsequent integration of C *K*-edge and Ca *L*_2,3_-edge spectra (see “Methods”). The C *K*-edge spectra of seven key areas of the FIB lamella, labeled on the HAADF image (Fig. 4a.), exhibit a series of peaks corresponding to C 1 *s* → π* electronic transitions^59^ (Fig. 4b). The intense peak at 290.3 eV is characteristic of carbonates, and the two small peaks at 285.1 and 287.5 eV, respectively denoted NZ_1_ and NZ_2_, are assigned to the presence of NZ based on the analysis of pure NZ (Supplementary Fig. 9a). The Ca *L*_2,3_-edge spectra of most areas exhibit the characteristic signature of calcite with two major peaks at 349.3 and 352.6 eV, as well as two minor peaks at 348.1 and 351.4 eV, in agreement with refs. ^60,61^. Areas 1, 2, 4, and 5, localized within or at the periphery of the 100 nm pores, clearly show the presence of NZ, as evidenced by the C *K*-edge peak at 285.1 eV (Fig. 4b). The homogenous distribution of NZ within the 100 nm pores is confirmed by the NZ_1_, NZ_1_/CO_3_ and NZ_1_/Ca maps as well as the RGB composite maps showing the spatial distribution of Ca, CO_3_ and NZ_1_ (Fig. 4c and Supplementary Fig. 9b, c). In addition, the Ca/CO_3_ maps reveal the enrichment in CO_3_ of the 100 nm pores, thus co-localizing with NZ. The bulk matrix, however, appears to contain less NZ on average (areas 3, 6, and 7, Fig. 4a, b). Nevertheless, the C *K*-edge spectra of some individual pixels (4 × 4 nm^2^) of area 7 also reveal the presence of NZ (pixels C and D, Fig. 4d), while others do not (pixels A, B and E, Fig. 4d). When not detected, the occurrence of NZ at a concentration below the detection limit cannot be excluded. However, these data do suggest a heterogeneous distribution of NZ at the nano-scale within the bulk calcite matrix of the Cal_0.53NZ_ sample.

To further study the possible incorporation of NZ molecules within the calcite lattice of the Cal_NZ_ samples at 72 h and the Cal:Vat_NZ_ samples at 700 s and 2.5 h, we performed synchrotron HR-XRD measurements^56,62–65^. The samples at 2.5 and 72 h show a splitting of certain calcite diffraction peaks, regardless of the presence of NZ (Fig. 4e, Supplementary Fig. 10a, b). Similar shoulders were observed in synthetic calcite with occluded amino acids^65^. This splitting is here attributed to two different calcite nanodomains as confirmed by Rietveld refinements (Supplementary Fig. 10a). Nanodomains corresponding to the component of this splitting at a high scattering vector (*q*) exhibit similar lattice parameters to those of typical geological and synthetic calcite^66^ and are named “calcite”. In contrast, nanodomains corresponding to the component at a low *q* show smaller *c*- and bigger *a*-parameters than typical calcite and are thus called “distorted calcite”. The latter exhibit anisotropic lattice distortions of ∆*c*/*c* = -0.02% to -0.07% and ∆*a*/*a* = 0.08% to 0.15% with respect to the former (Supplementary Fig. 10d). In average, the crystallite size of the distorted calcite nanodomains is also anisotropic and exhibit a bigger size distribution than calcite nanodomains with 397 ± 112 nm versus 316 ± 48 nm in the (110) direction and 1005 ± 568 nm versus 336 ± 41 nm in the (202) direction (Fig. 4g, and Supplementary Fig. 11a). These results suggest the incorporation of impurities within the calcite lattice of the distorted nanodomains of all samples. Importantly, samples after ACC crystallization at 700 s, only show distorted calcite nanodomains, which then transform into typical calcite with time and after heating (Fig. 4e, f and Supplementary Fig. 10c). This time-dependent and thermally activated reorganization indicates that the formation of these distorted nanodomains is likely due to the occlusion of water molecules within the calcite lattice during ACC crystallization.

Interestingly, while the presence of NZ in the Cal_NZ_ samples has no effect on the nanostructure of the calcite nanodomains, it does modify that of the distorted nanodomains. Indeed, it decreases their amount and crystallite sizes and slightly increases their lattice distortions with maximum values of 0.07% along the (001) plane (∆*a*/*a*) for Cal_0.53NZ_ (Fig. 4e–h, Supplementary Fig. 10c, e). These lattice distortion values are similar to those obtained for native sea urchin spines^62^, but are significantly smaller than those of synthetic calcite comprising organics within their crystalline lattices^63,65^, likely because of the lower concentration of occluded NZ. In addition, the Cal_0.53NZ_ sample exhibits a 0.15% increase in lattice distortions of its distorted nanodomains with respect to its calcite ones (Supplementary Fig. 10d). Overall, these results, therefore suggest that few NZ molecules are preferentially occluded anisotropically within the lattice of the distorted calcite nanodomains at the end of the reaction, while most of them are incorporated within isolated nano-occlusions, in agreement with the EELS data.

## Discussion

We previously demonstrated that the purple color of *P. lividus* sea urchin spines originates from the encapsulation of deprotonated spinochrome A within the calcite phase^10^. Here, we hypothesize that this mechanism occurs during calcite growth, shown to proceed through ACC precursors^21,60^. Our in-vitro study, considering NZ as an analogue to spinochrome A, validates this hypothesis by demonstrating that the decrease in pH induced by ACC precipitation and crystallization controls the protonation state of the dye, and thus the lavender blue and violet-blue colors of the hybrid pigments. In addition, similar CaCO_3_ synthesis performed in the presence of spinochrome A also leads to purple calcite, which exhibits the same UV-Vis spectral signature as that of purple sea urchin spines^10^.

**Fig. 5:**
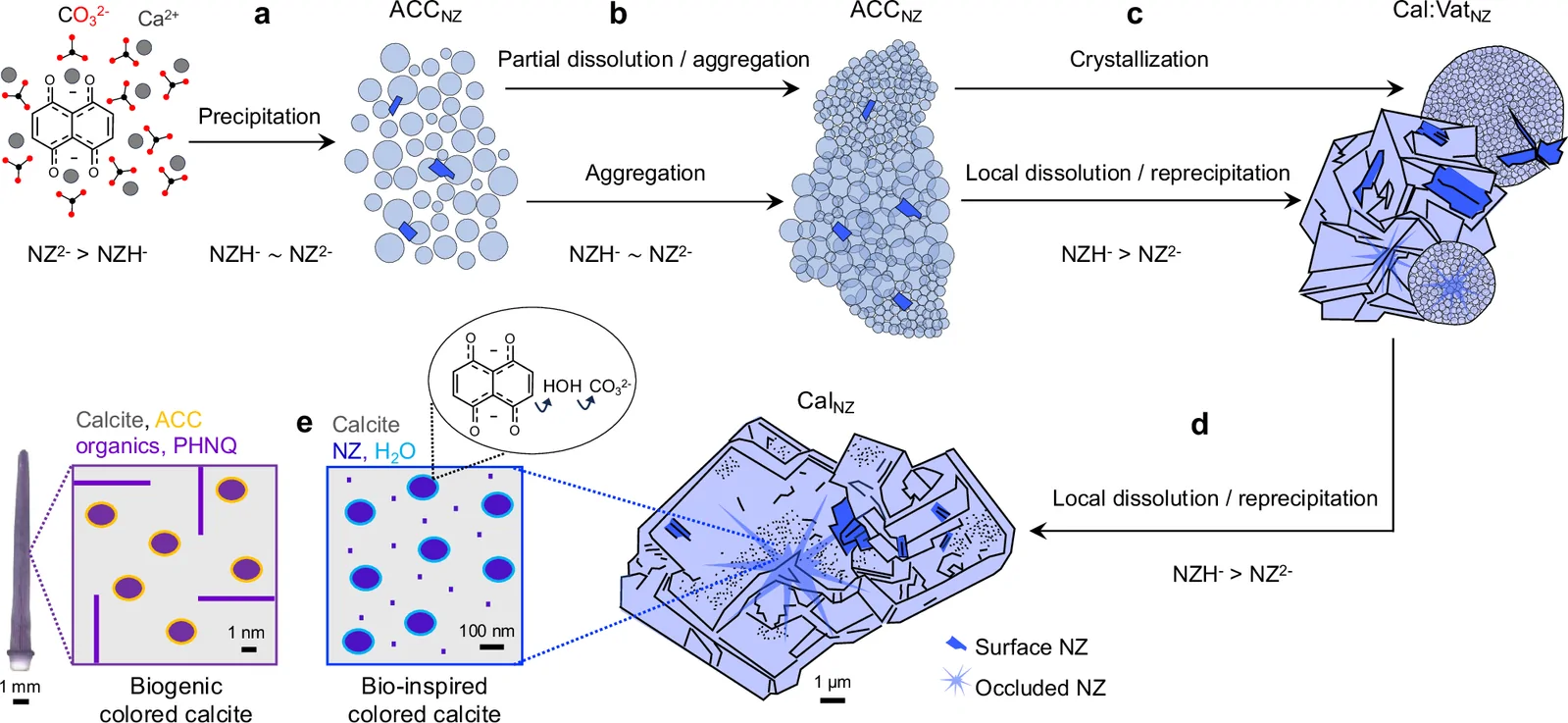
Schematic illustration of the proposed mechanism for the formation of bioinspired colored crystals. **a** ACC_NZ_ precipitation, **b** ACC_NZ_ particles partial dissolution and /or aggregation, **c** Cal:Vat_NZ_ formation where vaterite spheres are obtained after the crystallization of aggregated ACC particles, and calcite with distorted nanodomains is formed after the local dissolution of ACC particles and further reprecipitation, **d** Cal_NZ_ formation via the local dissolution and reprecipitation of vaterite and calcite distorted nanodomains, and **e** internal nanostructure of Cal_NZ_ with nano NZ-inclusions, the interaction between NZ, water and CO_3_ is also displayed and the internal nanostructure of sea urchin spines represented based on the results obtained in refs. ^62,80,81^. The protonation states of NZ in solution during the different transformations are indicated.

By combining the different results described above, we here propose a mechanism for the formation of such bioinspired colored crystals and discuss the role of deprotonated NZ on the formation and crystallization of ACC as well as on the final calcite nanostructure (Fig. 5).

During the first seconds of ACC precipitation, the formation of Ca^2+^- NZ^2-^ complexes might be responsible for the subsequent association of deprotonated NZ with ACC. While most of the NZ molecules present in solution are captured by the ACC phase, they do not influence its formation (nanoparticle size and local structure) nor its stability. Similar amounts of organic molecules with low molecular weights, such as aspartic acid and citric acid ( < 300 g mol^−1^, organics/Ca ≤ 1%), were also shown to have a minimal effect on ACC formation and stabilization^25,67^. However, these molecules are not incorporated into the ACC phase, unlike organics that significantly stabilize ACC^64,67,68^. Here, we therefore propose that NZ does not influence ACC formation due to short-range interactions with the calcium carbonate complexes, as proposed for aspartic acid^25^. Nevertheless, NZ^2-^-Ca^2+^ and NZH^-^-Ca^2+^ interactions appear strong enough to be responsible for the association of the ACC phase with nearly all the NZ molecules introduced in the Na_2_CO_3_ solution. Strong associations between ACC and PHNQs can also be expected in vivo, allowing ACC to serve as a pigment shuttle at the mineralization front, further emphasizing the critical role of amorphous precursors in the incorporation of organic molecules within biogenic calcite. In addition, NZ seems involved in pH regulation by lowering the pH of the Na_2_CO_3_ solution and capturing some protons after ACC precipitation, as it evolves from NZ^2-^ to NZH^-^. In vivo, we speculate that PHNQs may have a similar function, as they are known to be released from red cells after, e.g., microbial infection, where a pH decrease is essential, and are further encapsulated during biomineralization, which occurs near neutral pH^15^. Tuning the PHNQs protonation state could also increase their solubility, complexation with Ca^2+^ ions, and antioxidant activity^8,12^ before being incorporated into the final calcite through ACC crystallization.

In this in vitro study, the crystallization of ACC occurs mainly through local dissolution and reprecipitation mechanisms^27,29,48^ (Fig. 5). In particular, we propose that vaterite spheres form via the partial dissolution, aggregation and further crystallization of some ACC nanoparticles^27,47^ and that their conversion into calcite happens through local dissolution/reprecipitation at the vaterite-calcite interface^48^. The formation of the calcite phase with distorted nanodomains likely occurs via the local dissolution of aggregated ACC nanoparticles and further reprecipitation, in agreement with Zou et al.^47^. This mechanism could explain the occlusion of water molecules within the calcite lattice of the distorted nanodomains after ACC crystallization. Although distorted nanodomains have not yet been reported in sea urchin biominerals, low lattice distortions have been measured due to the presence of remnant ACC^62^. In addition, nano-crystalline domains^69^, also called internal nanocrystallinity^70^ or lattice distortions across scales^71^, have often been reported in biogenic and synthetic calcitic systems. Here, the transformation of the distorted nanodomains into typical calcite is proposed to proceed via local structural rearrangement, possibly through local dissolution/reprecipitation as well. We suggest that this transformation occurs from the surface to the inner part of the crystals, where some distorted nanodomains, enriched with NZ, remain present in the center of the final calcite crystals.

In addition, we show that NZ limits the presence of the distorted domains, perhaps by reducing the occlusion of water. It also limits the formation of vaterite and slows down its conversion into calcite, thereby slightly modifying the kinetic and thermodynamic formation of the final calcite. Acknowledging their importance in biomineralization, the role of hydroxyl-rich molecules in CaCO_3_ polymorph selection has been extensively studied^10,29,72–75^. In particular, negatively charged polyaspartic acid used in low concentrations was also shown to promote the nucleation and growth of calcite over vaterite^29,75^ and spinochrome A to delay the conversion of vaterite into calcite^10^. Hydroxyl-rich small molecules were also evidenced to strongly bind to calcite surfaces^76^ by producing a hydrophobic layer^77^, low-charged hydroxyl-rich macromolecules to incorporate in high amounts in calcite without affecting its structure^72^ and producing rhombohedral internal organic inclusions^58^, and charged hydroxyl-rich macromolecules to interact with specific calcite planes^56^. Although NZ bears two -OH groups, its protonation state varies during ACC formation and crystallization from fully deprotonated to partially deprotonated. NZ deprotonation limits, in principle, its adsorption and further incorporation into calcite, which is, on average, negatively charged under these non-equilibrium conditions^78^. Nevertheless, deprotonated NZ molecules here strongly adsorb on the calcite crystal surfaces, possibly on the (012) plane, which is positively charged as mainly composed of Ca^2+^^79^.

A scheme for the nanostructure of the violet-blue calcite is finally proposed (Fig. 5e), displaying heterogeneously distributed nano inclusions of NZ with the biggest ones enriched with NZ and CO_3_. The interaction between NZ molecules and calcite is likely mediated by water molecules, as in some biominerals, where organic inclusions are stabilized by water molecules interfaced with CaCO_3_^44^_._ The structure of the colored calcite resembles that of sea urchin spines^80,81^, allowing us to speculate that PHNQ molecules might be associated with the remnant ACC phase in the biominerals as well. Although low amounts of NZ are incorporated (0.05–0.15 wt.%) with respect to other organics^28,63,74^, they are similar to that of intra-crystalline PHNQs in purple sea urchin spines (0.1 wt.%)^10^. To achieve such biologically relevant concentrations within the crystals, the concentration of NZ had to be ten times higher in the reaction medium. This suggests that PHNQ local concentration at the site of mineralization must be quite high or that other molecules can chaperone the pigment occlusion^34^ by thus forming PHNQ-macromolecule complexes ready to be incorporated within sea urchin spines during biomineralization.

Inspired by the growth of colored sea urchin spines that occurs through ACC precursors, we have here demonstrated an in vitro halochromic modulation of calcium carbonate formation. These results contribute to our better understanding of the color origin of biominerals, where organic pigment deprotonation is driven by ACC crystallization. Hence, starting from a red dye, we synthesized an amorphous lavender-blue hybrid pigment that further crystallizes into violet-blue calcite whose nanostructure resembled that of sea urchin spines. While blue colors in nature typically arise from structural coloration^82^, our findings indicate that they can also be obtained by tuning red pigments via pH shifts, consistent with mechanisms observed in some plants^83^. Although these pigment molecules might not be directly involved in biomineralization pathways at the ACC stage, they surely participate in the formation of the final crystals by contributing to the polymorph selection and the emergence of complex composite nanostructures. Ultimately, this study may inspire the development of bio-sourced hybrid pigments for various applications such as more sustainable textile finishing^10^.

## Methods

### Materials

The reagents were purchased from Sigma-Aldrich and Carl Roth and used without further purification. These included NZ (C_10_H_6_O_4_) (85%), calcium chloride dihydrate (CaCl_2_⋅2H_2_O) (99.9%), sodium carbonate (Na_2_CO_3_) ( ≥ 99.5%), potassium bromide (KBr) ( ≥ 99%), sodium 3-(trimethylsilyl)-1-propanesulfonate (DSS) (97%), adamantane ( ≥ 99%), cerium oxide ( ≥ 99%), acetic acid ( ≥ 99.7%), sodium hypochlorite (NaOCl) with 12% active chlorine and free alkali (as NaOH) ≤ 2.0 %, 1 M sodium hydroxide (NaOH) solution ( ≥ 98%), 37% hydrochloric acid (HCl) ( ≥ 99.9%), deuterated water (D_2_O) (99.9 atom %), and absolute ethanol ( ≥ 99.8%). A Millipore Milli-Q® Integral 10 water purification system was used to obtain double distilled water with a resistivity of 18.2 MΩ cm at 25 °C and TOC < 5 ppb. Double distilled water was used in all experiments.

### CaCO_3_ syntheses and sample preparation

The Metrohm® Titrando setup is equipped with motorized pumps for the automatic addition of solutions at specific rates and intervals, as well as a pH electrode and a Ca-ion-selective (Ca-ISE) electrode. The pH electrode is calibrated with standard buffers of pH 4.01, 7.00, and 9.21, and the Ca-ISE electrode is calibrated with Ca^2+^ solutions of concentrations 0.1, 1, 10, and 50 mM, with pH adjusted to 10.5–11.0 using 1 M NaOH. Before and between each experiment, electrodes were thoroughly cleaned with 0.1 M acetic acid, rinsed with double-distilled water, and recalibrated to ensure accuracy. During all experiments, including calibration and synthesis, measurements of [Ca^2+^] and pH potentiometry curves were recorded at intervals of 0.1 or 1 s, depending on the experiment duration, with the Methrom® tiamo 2.5 software.

During synthesis, both electrodes are immersed in 48 mL of the 40 mM Na_2_CO_3_ solution containing varying [NZ] from 0 to 0.53 mM. Once pH is stabilized (typically within 1 to 5 min), 2 mL of 1 M CaCl_2_ solution is added at a rate of 15 mL min^−1^ under continuous magnetic stirring (300 rpm) to initiate the reaction, which occurs within a few seconds. We chose to synthesize ACC at a concentration of 40 mM, which yields 200 mg powder as a good compromise between ACC stability in air at this concentration^25^ and biologically relevant [NZ]. As soon as the synthesis is stopped, the precipitates are filtered using Büchner filtration through a sintered filter with a pore size between 10 and 16 µm and a diaphragm vacuum pump (Knf® type N860. 3FT.40.18). The precipitates are rinsed with cold ethanol during filtration and immediately transferred into a desiccator under vacuum for 25 min.

Crystalline polymorphs for XPS, solid-state NMR, STEM-EELS, and HR-XRD experiments at ID22 (ESRF) were synthesized using a Thermo Scientific™ Cimarec i Multipoint magnetic stirrer plate with multiple positions, instead of the Metrohm® Titrando setup. Under continuous stirring (at 300 rpm), 2 mL of 1 M CaCl_2_ solution was added to the reaction mixture (40 mM Na_2_CO_3_ + [NZ]) (48 mL) using a pipette. The addition time was approximately equivalent to that of Metrohm® titrando (8–10 s). The reaction products were filtered using Büchner filtration and rinsed with ethanol. The samples were then placed under vacuum in a vial for 30 min to remove any excess water or moisture.

For HR-XRD measurements, the Cal and Cal_0.11NZ_ samples were annealed at a heating rate of 5 °C min^−1^ to temperatures ranging from 150 to 400 °C in a Nabertherm furnace, and the Cal:Vat_0.11NZ_ sample was annealed at 250 °C for 0.5 h direclty at the beamline. All annealed samples were measured at room temperature, as they exhibit intrinsic calcite expansion upon heating.

The CalxNZ and KBrxNZ samples were prepared by mixing 200 mg of Cal and KBr powder, respectively, with 1 to 24 mg of NZH_2_ powder, and then grinding in a mortar with a pestle for 10 min, resulting in 0.5 to 12 wt.% NZ.

For solid-state NMR measurements, lyophilized deprotonated NZ powders were obtained by analogy to a previous study that determined the acid-base properties of lyophilized histidine powder by evidencing significant ^13^C peak shifts according to pH^84^. Hence, 0.4 mg mL^−1^ NZ solutions were prepared by adjusting the pH to 9 and 12 with NaOH, further frozen at -80 °C for 2 h, and subsequently freeze-dried. These samples are named respectively NZH^-^ powder and NZ^2-^ powder.

For liquid-state NMR, three solutions at pH levels of 11, 9, and 6 were prepared by dissolving 0.1 mM of NZ in D_2_O and adjusting the pH using 40 mM NaOH prepared in D_2_O.

For STEM-EELS, a FIB lamella was obtained from a Cal_0.53NZ_ sample, further bleached for 2 h with 1% NaOCl. The FIB-lamella was obtained using a Zeiss Crossbeam 540 equipped with a Ga-ion source. Before cut-out and thinning of the lamella, a Pt-layer of a few microns thickness was deposited on and around the particle to enable a large enough cross-section area for lamella preparation. The lamella was prepared by subsequent milling with a 30 kV:30 nA, 30 kV:3 nA, and 30 kV:1.5 nA FIB-probe. After lift-out, the lamella was attached to a TEM lamella holder and further thinned and polished with 30 kV:100-20 pA FIB-probes to expose a transparent window of ≈5 × 5 µm^2^. Once lamella transparency was achieved, the lamella surfaces were exposed to a 5 kV:5 pA beam to remove the amorphous layer. The final thickness of the lamella was about 50 nm.

### Stereo microscopy

Powder photographs were obtained using a Zeiss Stemi 508 equipped with an AxioCam ICc 5 camera, with a magnification ratio of 0.5 to 1. Samples were illuminated with a polarized ring light. Exposure and colors were calibrated using a QPcard 101 and a X-rite ColorChecker Classic Mini, respectively. Images were acquired with the Zen core v3.8 software and further processed using RawTherapee 5.10 software.

### Light microscopy

Light microscopy was performed on glass slides using a transmission Zeiss AxioImager A2 POL, equipped with an AxioCam CCD camera. Images were acquired with the Zen core v2.7 software.

### Liquid UV-Vis spectroscopy

Liquid UV-Vis spectroscopy was performed using a spectrometer (CARY 5000, Agilent Technologies) operated in scan mode from 200 to 800 nm, with a scan speed of 600 nm s^−1^ in absorbance mode. Absorbance values were recorded at 515 nm with the Cary Win UV scan Application software v 6.2.0.1588.

The amount of NZ associated with ACC and calcite was measured through UV-Vis spectroscopy. This involved dissolving the ACC_NZ_ and Cal_NZ_ samples in a 1 M HCl solution, resulting in a clear, red-colored solution. Before obtaining the absorbance spectrum, the solutions were filtered. The solution’s pH was adjusted to 5.5, at which a calibration curve was established. For establishing a calibration curve at pH 5.5, NZ was dissolved in water at various concentrations ranging from 0.01 to 0.055 mM due to its low solubility of 0.01 mg mL^−1^ at pH 5.5.

Calculation of [Ca^2+^] in the solution after CaCO_3_ dissolution:

1 $$[{{{\rm{Ca}}}}^{2+}]({{\rm{mM}}})=\frac{{{\rm{Amount}}}\; {{\rm{of}}}\; {{\rm{sample}}}\; {{\rm{used}}}\; {{\rm{for}}}\; {{\rm{the}}}\; {{\rm{quantification}}}\left({{\rm{mg}}}\right)\times 0.4\,}{{{\rm{molar}}}\; {{\rm{mass}}}\; {{\rm{of}}}\; {{\rm{Ca}}}\left({{\rm{g}}}{{{\rm{mol}}}}^{-1}\right)\times 0.05{{\rm{L}}}}$$

0.4 corresponds to 40% of Ca in CaCO_3_

Calculation of [NZ] associated with CaCO_3_ adjusted to the ideal yield of the synthesis:

2 $$[{{\rm{NZ}}}]({{\rm{mM}}})=\frac{\,\left[{{\rm{NZ}}}\right]{{\rm{obtained}}}\; {{\rm{from}}}\; {{\rm{the}}}\; {{\rm{calibration}}}\; {{\rm{curve}}}\left({{\rm{mM}}}\right)\times 200{{\rm{mg}}}}{{{\rm{Amount}}}\; {{\rm{of}}}\; {{\rm{sample}}}\; {{\rm{used}}}\; {{\rm{for}}}\; {{\rm{the}}}\; {{\rm{quantification}}}({{\rm{mg}}})}$$

3 $$[{{\rm{NZ}}}]({{\rm{wt}}}.\%)=\frac{\left[{{\rm{NZ}}}\right]\left({{\rm{mM}}}\right)\times {{\rm{molar}}}\; {{\rm{mass}}}\; {{\rm{of}}}\; {{\rm{NZ}}}\left({{\rm{g}}}{{{\rm{mol}}}}^{-1}\right)\times 0.05{{\rm{L}}}}{200{{\rm{mg}}}}\times 100$$

0.05 L of solvent was used to dissolve the powder samples, 200 mg is the ideal yield for the synthesis of 40 mM CaCO_3_, M(NZ) = 190.15 g mol^−1^, M(NZ^-^) = 189.15 g mol^−1^ and M(NZ^2-^) = 188.15 g mol^−1^.

pK_a_ calculations

The dissociation constants for NZH₂, NZH⁻ and NZ²⁻ have been first reported by Land et al.^37^, with pK_a1_ = 7.85 and pK_a2_ = 10.7. These values were determined in aqueous NaOH solution, where the pH was modulated by titration with HCl. Experimental absorbance values (*A*) corresponding to different pH conditions were fitted using Eq. (4) to extract the pKₐ values^37^.

4 $$A=	 \frac{{A}_{1}}{\left(1+{10}^{{{\rm{pH}}}-{{{\rm{pK}}}}_{1}}+{10}^{{{\rm{pH}}}-{{{\rm{pK}}}}_{2}}\right)}+\frac{{A}_{2}}{\left(1+{10}^{{{\rm{pH}}}-{{{\rm{pK}}}}_{1}}+{10}^{{{\rm{pH}}}-{{{\rm{pK}}}}_{2}}\right)} \\ 	+\frac{{A}_{3}}{\left(1+{10}^{{{\rm{pH}}}-{{{\rm{pK}}}}_{1}}+{10}^{{{\rm{pH}}}-{{{\rm{pK}}}}_{2}}\right)}$$

where *A*_1_, *A*_2_, and *A*_3_, refer to absorbances attributed to the forms NZH_2_, NZH^-^ and NZ^2-^, respectively, and pK_1_ and pK_2_ are the pKa values of NZH_2_, and NZH^-^.

To gain insight into the pK_a_ values of NZ during CaCO₃ crystallization, UV-Vis absorption spectroscopy was therefore performed on  NZ solutions across a range of pH values adjusted with HCl in the presence of Na₂CO₃ (Supplementary Fig. 4b). To precisely quantify these protonation equilibria, the pK_a_ values of NZ in Na₂CO₃ solutionswere determined by fitting the absorbance spectra across the pH range using Eq. 4. Two distinct pK_a_ values, pK_1_ = 8.2 and pK_2_ = 10.3, were obtained.

### UV-Vis DRS

UV-Vis DRS was performed on powder samples using the CARY 5000 spectrometer (Agilent Technologies) with an integrating sphere, which consists of a spherical inner surface made of barium sulfate. Measurements were performed in scan mode, with a wavelength range of 200-800 nm and a scan speed of 600 nm s^−1^. The powder samples were packed in a cell holder covered by a quartz window. BaSO_4_ was used as a reflectance standard. The diffuse reflectance (*R*) spectrum of each sample was measured and converted into absorbance by the Cary Win UV software v 6.2.0.1588, using *A* = Log (1/*R*). The chromaticity CIE-1931 diagram was generated using a plug-in in OriginPro that allows for the calculation of *x*, *y* values, and L*, a*, b* color parameters based on the measured reflectance (*R*) values. L* a* b* is a uniform color space recommended by the CIE, where L*, a*, and b* correspond to the lightness, the red-green axis, and the yellow-blue axis, respectively^85^. sRGB values and simulated color patches were subsequently calculated using the converter of the nixsensor.com website based on the above-mentioned L* a* b* parameters as well as the D65 illuminant and 2° reference angle.

### XPS

XPS analyses were performed with a Thermo Scientific ESCALAB 250xi spectrometer using a monochromatic Al *K*_α_ X-ray source. The acquisition parameters were a spot size of 650 μm, a primary energy of 14.3 kV, an emission intensity of 15.0 mA, a CAE mode (20 eV), and an energy step of 0.1 eV. The use of a low-energy electron and ion flood gun was necessary to perform the analysis. The sample preparation was carried out by suspending the samples in absolute ethanol. A few 100 µL of the solutions were deposited on the sample holder, followed by rapid solvent evaporation before introduction into the XPS chamber (dried droplet method). Spectra were acquired and analyzed using the Avantage© softwares v5.9931 and v5.9925, respectively. The quantification was obtained considering C 1 *s*, O 1 *s*, and Ca 2*p* peaks after Shirley-type background subtraction. Photopeaks were reconstructed considering C *sp*^2^, -C-OH, -C = O, -O-H contributions. The data were calibrated with respect to the carbonate peak of the C 1 *s* spectral region.

### FEG-SEM

The ACC particles, as well as vaterite and calcite crystals, were imaged by a FEG-SEM Hitachi SU-70 microscope (PC_SEM.3.1 software). The samples were spread on carbon tape and then coated with a 10 nm Pt layer. The samples were examined at an accelerating voltage ranging from 2 to 5 kV under high-vacuum conditions. The FIJI software v 2.0.0-rc-69 was used to analyze the particle size.

### XRD and HR-XRD

XRD measurements were acquired on a Bruker D8 DISCOVER diffractometer (at 40 kV, 30 mA, 0.010° step, and 2*θ* of 5° − 80° with the COMMANDER v8.61 software. A Cu tube (*K*_α_, *λ* = 1.54 Å) was used.

HR-XRD measurements were performed at the ID22 beamline (ESRF, France) at 35 keV with a 1 × 1 mm^2^ beamsize and 2*θ* of 0.5° − 50°. Data were acquired on a rotating 0.7 mm capillary filled with the sample powders. The beamline uses a 13-channel Si 111 multi-analyzer stage with a Dectris Eiger2 X 2M-W CdTe detector^86,87^. Data were normalized and rebinned into steps of 0.001° using the standard local software (id22sum). Rietveld refinements were conducted using the Fullprof software suite with the derivative difference method approach^88^ for the D8 XRD data, and with the GISAS II software^89^ for the ID22 HR-XRD data. In addition, crystalline sizes from the HR-XRD data were extracted after peak fitting with a Voigt function following ref. ^79^.

### X-ray scattering for PDF analysis

Measurements were carried out at ID15A beamline (ESRF, France). X-ray scattering data were acquired using a Pilatus CdTe 2 M with 0.5–1 s measurement time. Beam energy was set to 60 keV with a beam size of 0.15 × 0.15 mm^2^. The calibration was performed with a CeO_2_ powder standard. The samples were packed within a 1 mm-thick copper plate featuring a central aperture designed to accommodate the sample. The PDFs were calculated with the PDFget × 3 software^90^.

### Liquid-state NMR

NMR experiments were recorded at a magnetic field of 11.7 T using a Bruker Avance III spectrometer equipped with a TCI cryoprobe. The NMR spectra were recorded at a temperature of 25 or 37 °C in D_2_O. A DSS (0.1 mM) solution was used as an internal reference for calibrating the chemical shift. The data were acquired and analysed using the Bruker Topspin software 3.6.3.

### Solid-state NMR

NMR experiments were performed at a magnetic field of 7.05 T using a Bruker Avance III spectrometer equipped with a 4 mm HX probe. The spectra were acquired under magic-angle spinning conditions at a frequency of 14 kHz. ^1^H and ^13^C were referenced using adamantane at 1.71 and 37.7 ppm, respectively.

^1^H-^13^C cross-polarization was obtained with a contact time of 1-2 ms and radio-frequency fields of 62 and 48 kHz for ^1^H and ^13^C, respectively. 93 kHz SPINAL-64 ^1^H decoupling was used. RD was adjusted to 1.3 * *T*_1_, corresponding to 19.6, 16.4, and 2.2 s for NZ, NZ^2-^, and Cal_NZ_ samples, respectively. The number of scans was 112, 400, and 1024, respectively. For 2D HETeronuclear CORrelation (HETCOR) experiments, 100 kHz Frequency-Switched Lee-Goldburg (FSLG) ^1^H homonuclear decoupling was added with 48-96 time increments. The data were acquired and analysed using the Bruker Topspin software 3.7.

### HAADF-STEM microscopy and EELS

HAADF-STEM imaging and EELS analysis were conducted using a monochromated Cs-corrected Nion Hermes 200-S STEM operated at 100 kV, equipped with a Nion Iris spectrometer, a single-tilt cryo-specimen holder (Henny Z), and a Merlin Direct Electron Detector camera (Quantum Detectors, UK). The convergence semi-angle was set to 10 mrad and the energy resolution to ≈150 meV by adjusting the monochromator slit (half-width of the zero-loss peak). The electron beam was scanned over the area of interest and a complete EEL spectrum was acquired with the Nion Swift software v16.9.1 at each position simultaneously with the HAADF image. A dose-effect study (doses ranging from 10 to 2,900 e- Å) was conducted on pure NZ to find a compromise between spatial resolution, signal-to-noise ratio, and electron beam damage (Supplementary Fig. 9a). An electron dose of 400 e- Å^-2^ was determined to be optimal (pixel size of 2 nm, a dwell time of 5 ms, and a beam current *I* = 6 pA). Core-loss EEL spectra were then acquired on the FIB lamella with an energy dispersion of Δ*E* = 0.11 eV per channel in the range between 280 and 355 eV, covering the characteristic atomic edges of C *K* and Ca *L*_2,3_. The energy scale calibration relies on ref. ^61^, specifically the peaks associated with the carbonate peak at 290.3 eV and the Ca *L*_2,3_-edge peaks at 349.3 and 352.6 eV. Regions of interest were localized by HAADF-STEM imaging before performing EELS chemical analysis. EELS data were acquired in the spectrum-imaging mode^91^, recording a spectrum covering the entire C *K*- and Ca *L*₂,₃-edge range at each position. A typical hyperspectral image covers ≈500 × 500 nm² and contains ≈10⁴ spectra. To enhance the signal per pixel and improve the signal-to-noise ratio for mapping, EELS raw spectrum-images were binned by a factor of 2, resulting in a 4 nm pixel size (final spatial resolution) and, after gain correction, data were denoised using the Gatan’s Digital Micrograph software v3.62.4986.0 via PCA (3 and 6 components for carbon and calcium spectra, respectively) to minimize noise while preserving small spectral features. Background subtraction was then applied, and the integral of the intensity of the spectroscopic features provided abundance maps for Ca, NZ, and CO_3_. The ratios NZ_1_/Ca, NZ_1_/CO_3,_ and CO_3_/Ca were calculated by dividing the corresponding individual maps.

### Statistics and reproducibility

Each sample was replicated about 10 times resulting in about 134 samples synthesized for this study. All replicates showed no significant variation after characterization. pH and Ca measurements were repeated about 10 times, light and SEM microscopy images were acquired on about 20 crystals (Fig. 3b-d). Stereo microscopy images (Fig. 1d), spectroscopy (UV-visible, NMR) spectra and XRD profiles were performed on several mg of powders, therefore providing good statistics of the bulk samples. About 20 EELS spectra were acquired within the FIB lamella and 7 HAADF maps acquired (Fig. 4a, c).

### Reporting summary

Further information on research design is available in the Nature Portfolio Reporting Summary linked to this article.

## Supplementary information


Supplementary Information
Reporting Summary
Transparent Peer Review file


## Data Availability

The data that support the finding of this study are available from Zenodo^92^ and from the corresponding authors upon request.
