# Supplementary material for: Halochromic modulation of amorphous calcium carbonate crystallization driven by pH-responsive bioinspired pigments
Source: Nat Commun. 2026 Aug 3;17:9378. doi: 10.1038/s41467-026-75888-8 (PMC13534625; doi:10.1038/s41467-026-75888-8)
Supplement: Supplementary file 1 [file 41467_2026_75888_MOESM1_ESM.pdf]

Supplementary information

Halochromic modulation of amorphous calcium carbonate crystallization driven by pH-responsive bioinspired pigments

Vaskar Sardhalia[1], Claudio Dos Reis Ferreira[1], Guillaume P. Laurent[1], Mohamed Selmane[2], Anne Vallée[3], Mathieu Frégnaux[4], Xiaoyan Li[5], Marta de Frutos[5], Andrew Fitch[6], Clemens N.Z. Schmitt[7], Shahrouz Amini[7], Zhaoyong Zou[8], Iryna Polishchuk[9], Boaz Pokroy[9], Peter Fratzl[7], Thierry Azaïs[1], Nadine Nassif[1] and Marie Albéric*[1]

[1] Sorbonne Université, CNRS, Chimie de la Matière Condensée de Paris, LCMCP, F-75005 Paris, France

[2] Sorbonne Université, CNRS, Fédération de Chimie et Matériau de Paris Centre, FCMat, F-75005 Paris, France

[3] Sorbonne Université, CNRS, Réactivité de Surface, LRS, F-75005 Paris, France

[4] Université Paris-Saclay, UVSQ, CNRS, Institut Lavoisier de Versailles, 78000 Versailles, France

[5] Université Paris-Saclay, CNRS, Physique des Solides, LPS, 91405 Orsay, France

[6] European Synchrotron Radiation Facility, 38000 Grenoble, France

[7] Max Planck Institute of Colloids and Interfaces, Biomaterials Department, 14476 Potsdam, Germany

[8] State Key Laboratory of Advanced Technology for Materials Synthesis and Processing, Wuhan University of Technology, 430070 Wuhan, China

[9] Technion Israël Institute of Technology, Department of Materials Science and Engineering, 3200003 Haifa, Israël

*corresponding author: marie.alberic@sorbonne-universite.fr

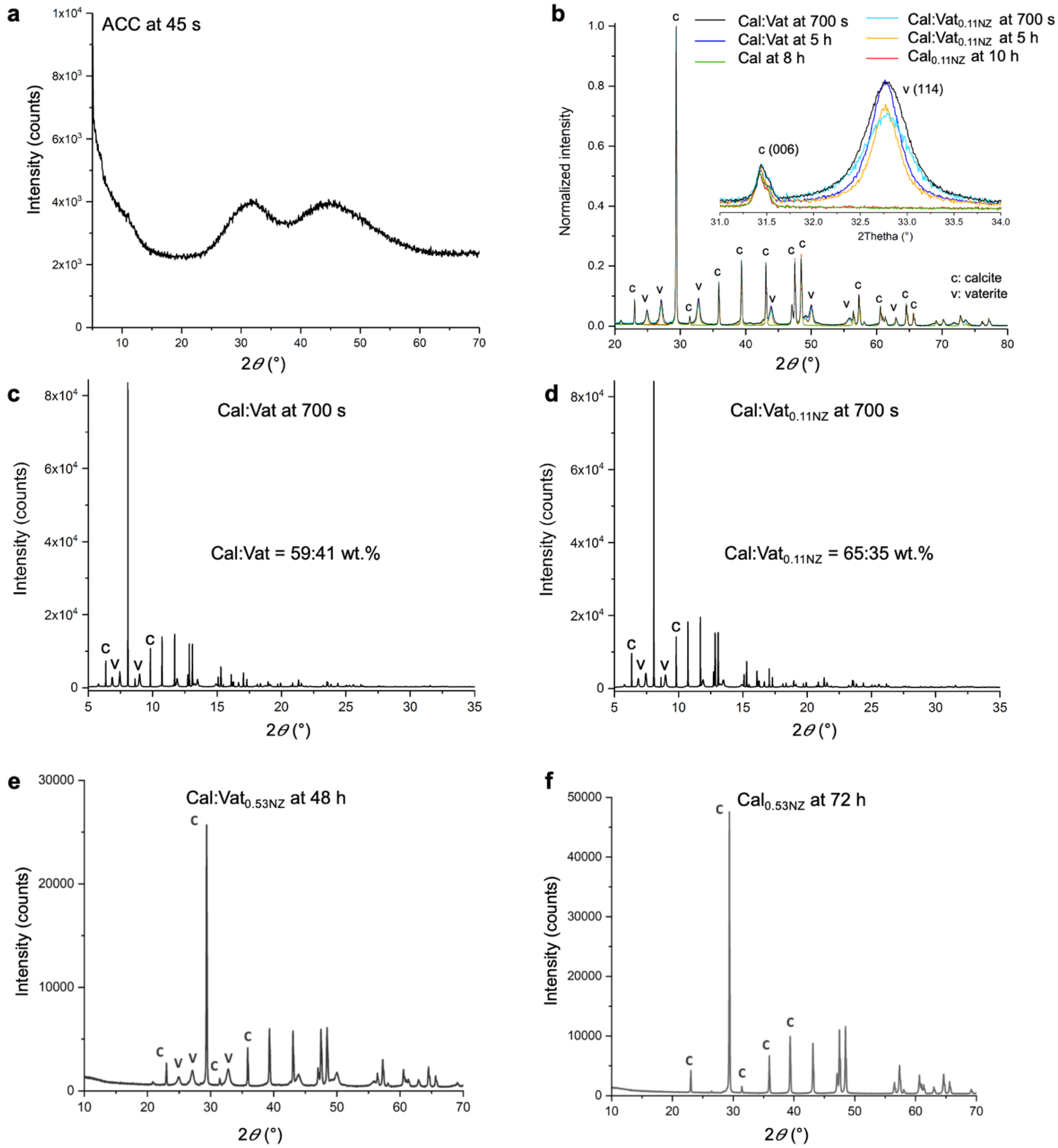

Supplementary Fig. 1. $CaCO_3$ polymorphs identification. Laboratory XRD profiles of **a)** ACC, **b)** Cal:Vat, Cal, Cal:Vat$_{0.11NZ}$ and Cal$_{0.11NZ}$ samples taken at different times of the reaction, **c-d)** synchrotron HR-XRD profiles of the Cal:Vat and Cal:Vat$_{0.11NZ}$ samples at 700 s with the Cal:Vat ratio obtained from Rietveld refinements and **e-f)** laboratory XRD profiles of Cal:Vat$_{0.53NZ}$, showing a mixture of calcite and vaterite at 48 h and showing only calcite at 72 h.

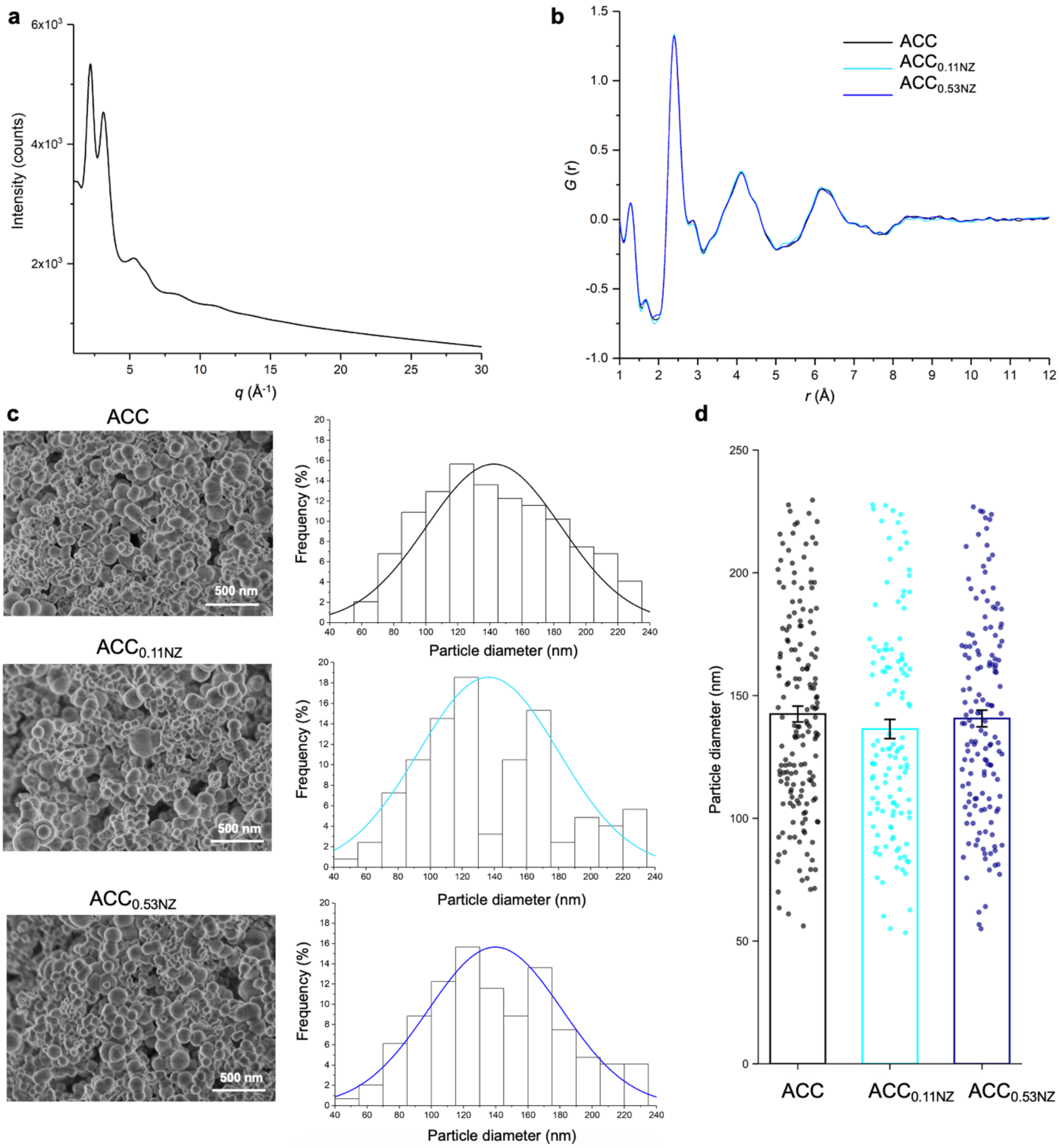

Supplementary Fig. 2. ACC characterization. a) XRD profile of ACC acquired at the synchrotron ID15A beamline (ESRF, France), **b)** PDF analysis of ACC (black), $ACC_{0.11NZ}$ (cyan) and $ACC_{0.53NZ}$ (blue) calculated from ID15 data that all show the same characteristic inter-atomic distances with positions at 2.4, 2.9, 4.1 and 6.2 Å, containing respectively the contributions from Ca-O pairs, O-O pairs, Ca-O/Ca-Ca pairs (1st shell) and Ca-O/Ca-Ca pairs (2nd shell) and exhibiting short and medium-range order up to 10 Å, **c)** characteristic SEM micrographs of ACC, $ACC_{0.11NZ}$ and $ACC_{0.53NZ}$ with their respective nanoparticle size distributions plotted in the forms of histograms and gaussian functions and **d)** bar chart with the corresponding data points as dot plots for ACC (n= 168), $ACC_{0.11NZ}$ (n= 124) and $ACC_{0.53NZ}$ (n= 146), the error bar correspond to the standard error of the mean.

Supplementary Table 1. *L**, *a**, *b** values calculated from the UV-visible diffuse reflectance spectra as well as sRGB values and simulated colors calculated with the color convertor of the nixsensor.com website.

Samples	*L**, *a**, *b**	sRGB values	Simulated colors
ACC	99.3, 0.1, 0.2	253 ,253, 253	
ACC$_{0.11NZ}$	76.9, 3.9, -18.6	179, 189, 224	
ACC$_{0.53NZ}$	59.3, 6.8, -21.6	135, 141, 180	
Cal	97.2, 0.1, 0.2	247, 247, 247	
Cal$_{0.11NZ}$	62.0, 4.7, -14.3	146, 148, 175	
Cal$_{0.53NZ}$	49.9, 4.9, -12.2	117 ,117, 139	

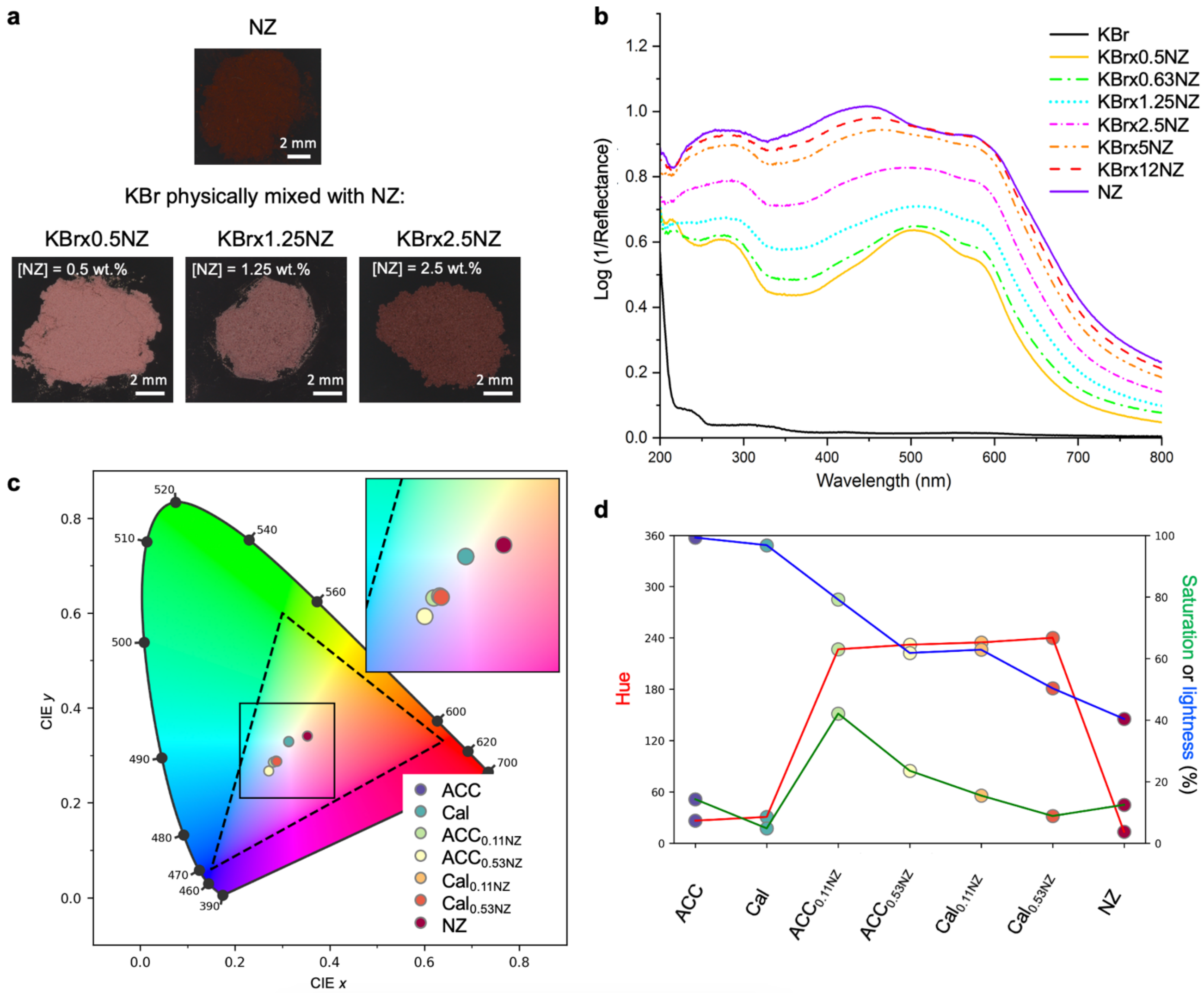

Supplementary Fig. 3. Color quantification of all samples. a) Stereo microscope images of NZ powder and powders obtained by physical mixing of KBr and NZ in different amounts from 0.5 to 12 wt.%, and **b)** corresponding UV-visible spectra acquired by DRS measurements, **c)** CIE 1931 chromaticity diagram of NZ, ACC, $ACC_{0.11NZ}$, $ACC_{0.53NZ}$, $Cal_{0.11NZ}$, and $Cal_{0.53NZ}$ showing the respective hues with **d)** the saturation and lightness parameters that better allow for distinguishing the different colors of the samples.

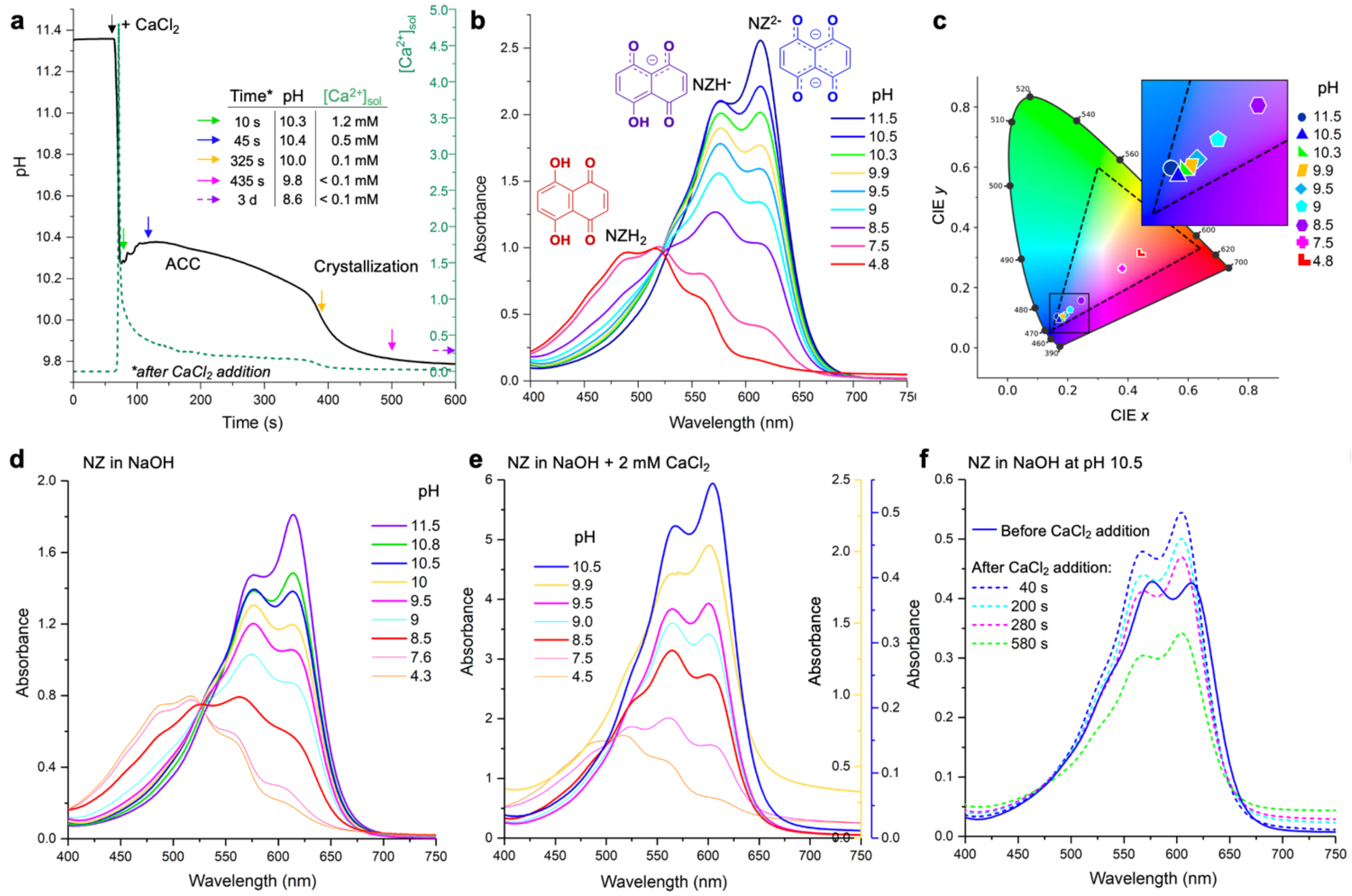

Supplementary Fig. 4. UV-vis in solution. a) pH and free [Ca^{2+}] profiles during ACC precipitation and crystallization in the presence of 0.53 mM NZ, pH and [Ca^{2+}] values at characteristic time points are summarized in the table (insert), **b)** UV-visible spectra of 40 mM Na_2CO_3 solutions with 0.53 mM NZ at pH from 11.5 to 4.8 adjusted with 0.1 M HCl, **c)** CIE 1931 diagram obtained based on the spectra shown in b) and UV-visible spectra of 40 mM NaOH solutions at pH from 11.5 to ≈4.5 adjusted with 0.1 M HCl with **d)** 0.53 mM NZ without Ca and **e)** 0.11 or 0.53 mM NZ with 2 mM $CaCl_2$, and **f)** of a 40 mM NaOH solution with 0.11 mM NZ at pH 10.5 before and after the addition of 2 mM $CaCl_2$ from 40 s to 580 s.

Supplementary Note 1

Supplementary Fig. 4b illustrates the progressive changes in the intensities of the UV-visible spectral bands as the pH increases. At pH 4.8 and pH 7.5, the solution is red, and the 515 nm band, attributed to the NZH_2 form, is the most intense. At pH 8.5, the solution is violet and the 575 nm band that corresponds to the NZH^- form is the most intense whereas at pH 10.3, the solution turns violet-blue and the band at 575 nm shows equal intensity to the one at 615 nm, attributed to the NZ^{2-} form, and thus indicating the presence of both NZH^- and NZ^{2-} forms. Finally, at pH 11.5, the 615 nm band is the most intense, and the solution is deep blue, due to the presence of NZ^{2-} only. We note the significant

decrease in the extinction coefficient with decreasing pH, likely due to the aggregation of the NZ molecules [1], which are less soluble at acidic pH (0.06 mM at pH 5.5) than at basic pH (2.2 mM at pH 11.5).

Supplementary Fig. 4d and 4e display the UV-Vis spectra of NZ measured immediately after the addition of 2 mM Ca^{2+}. They show the characteristic NZ absorption bands described above. However, the relative intensity of those bands, depending on pH values, differs from that in the absence of Ca^{2+}.This results in a decrease in the pK_a values by one pH unit. Indeed, pK_a values extracted by fitting the absorbance spectra acquired using equation 1 yielded to $pKa_1 = 7.5$ and $pKa_2 = 9.4$.
Finally, the spectra measured at pH 10.5 as a function of time until 580 s after Ca^{2+} addition (Supplementary Fig. 4f), exhibit the characteristic NZ absorbance bands whose intensities only decrease slightly, indicating no significant oxidative decomposition of NZ in these conditions.

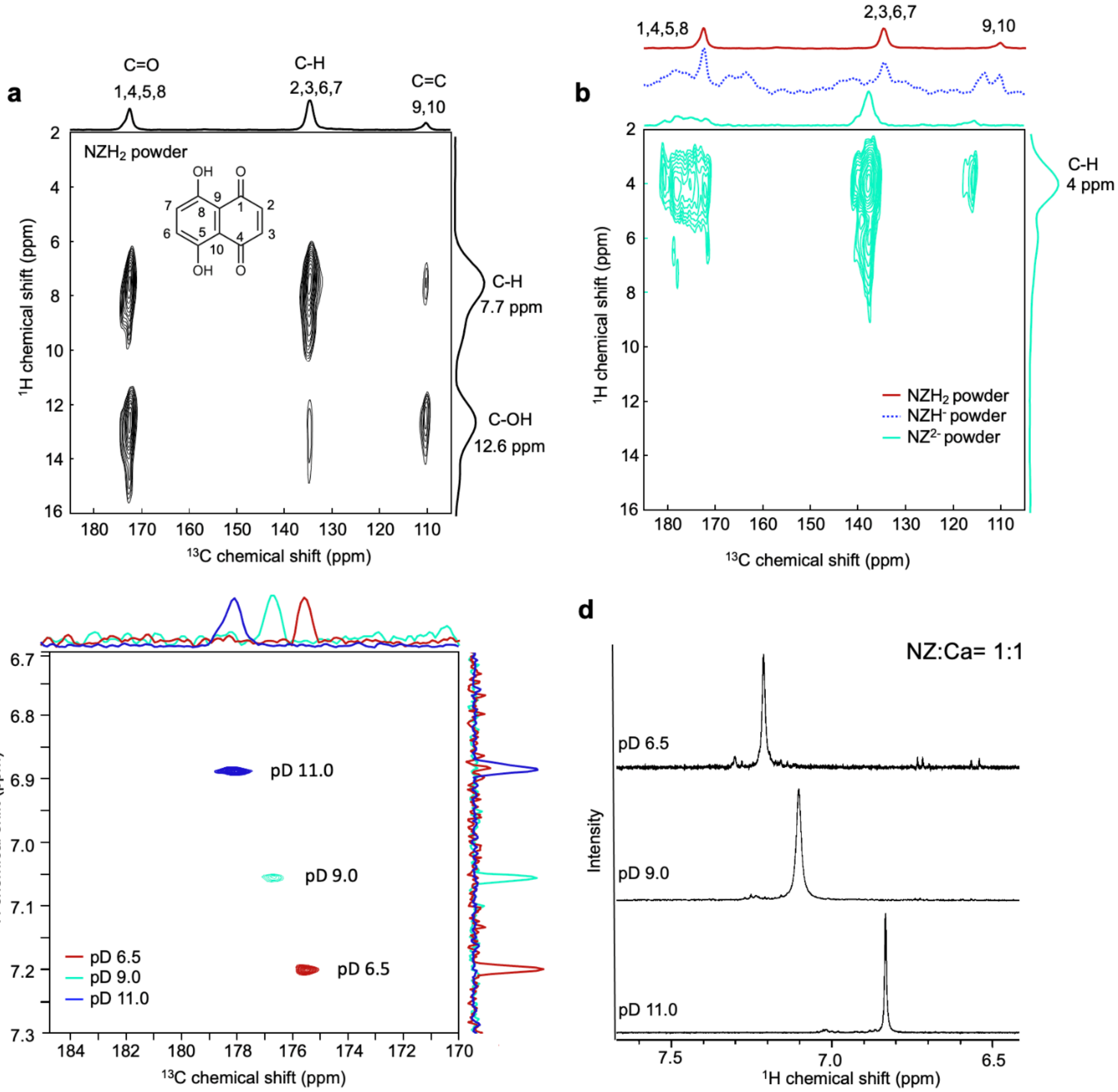

Supplementary Fig. 5. NMR data of the reference samples. 2D ^{1}H-^{13}C HETCOR solid-state NMR spectrum of **a)** NZ powder in its protonated form (NZH_2) that shows the correlations between the three carbon resonances at 173 ppm for C=O, 135 ppm for =C-H and 110 ppm for C=C with the two proton resonances at 7.7 ppm for =C-H and 12.6 ppm for C-OH and **b)** NZ prepared at pH 12 (NZ^{2-} powder) that shows a shift to lower ppm values of the ^{1}H signals and the decrease of the C-OH resonance with increasing pH. The ^{13}C CPMAS NMR spectra of NZH_2 powder, and NZ prepared at pH 9 (NZH^{-} powder) are also shown, **c)** ^{1}H-^{13}C 2D HMBC liquid-state NMR spectra of three NZ solutions prepared in D_2O at pD = 6.5 (gold), 9 (red) and 11 (blue) measured at 25 °C for pD = 11 and 37 °C for pD = 6.5 and 9.0 to increase NZ solubility. The ^{13}C resonance corresponding to the 1,4,5,8 carbons, shifts toward higher ppm values with pH increase (from 175.6 ppm to 176.4 ppm and to 178.2 ppm)

whereas the ^{1}H resonance to lower ppm values (from 7.2 ppm to 7.06 ppm to 6.89 ppm) and **d)** ^{1}H spectra of the tree solutions of c) in the presence of Ca^{2+} ions (NZ:Ca, 1:1), which exhibit smaller ^{1}H peak shifts than the ones evidenced upon pH variations. This suggests that peak shifts observed for $Cal_{0.53NZ}$ (Fig. 2d) are more likely due to NZ deprotonation rather than to Ca complexation. Chelation of metallic ions with anthraquinone dyes was previously reported to also induce ^{1}C peak shifts [2,3].

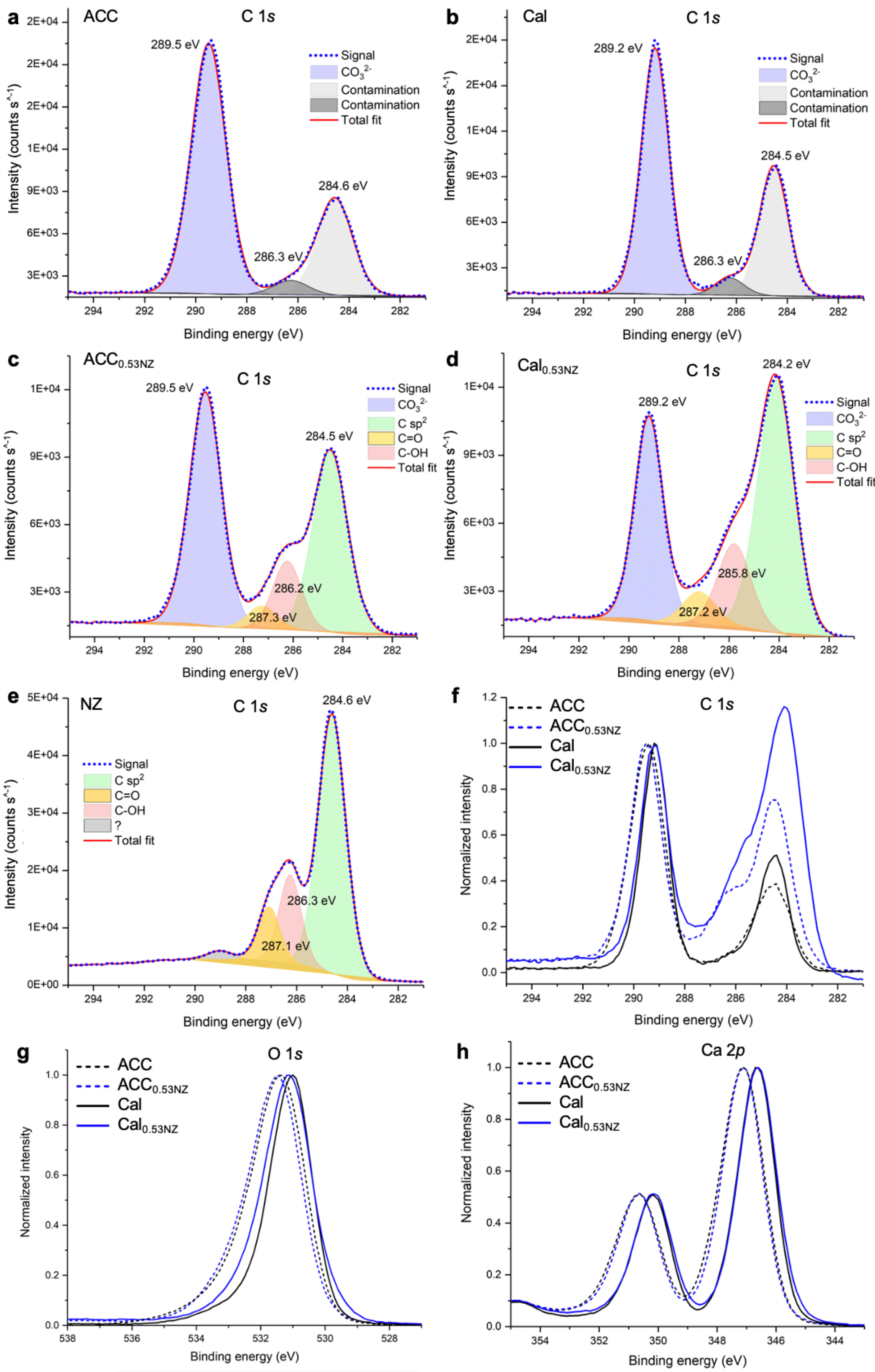

Supplementary Fig. 6. XPS data. XPS spectra of C 1*s* and fitted components (CO_3^{2-}, C *sp*^{2}, C-OH and C=O) of **a)** ACC, **b)** Cal, **c)** $ACC_{0.53NZ}$, **d)** $Cal_{0.53NZ}$, **e)** NZ, the peaks at 284.5/6±0.1 and 286.3±0.1 eV for Cal and ACC are due to the presence of carbonaceous contamination, the peaks at 287.3±0.1 and

287.2±0.1 eV, 286.2±0.1 and 285.8±0.1 eV as well as 284.4±0.2 eV and 284.2±0.2 eV for $ACC_{0.53NZ}$ and $Cal_{0.53NZ}$ are respectively attributed to the C=O bonds, C-OH bonds and the C sp^2 bonds of the NZ molecules, **f)** C 1*s*, **g)** O 1*s* and **h)** Ca 2*p* core levels of ACC, $ACC_{0.53NZ}$, Cal and $Cal_{0.53NZ}$ samples. Notably, the carbonate contributions on the C 1*s*, O 1*s* and Ca 2*p* core levels of ACC exhibit higher binding energies than those of calcite (289.2±0.1 eV) and are closer to that of aragonite [4].

Supplementary Table 2. NZ content in solution before ACC precipitation as well as in the solid amorphous and calcite samples $ACC_{0.11NZ}$, $ACC_{0.53NZ}$, $Cal_{0.11NZ}$ and $Cal_{0.53NZ}$ (before and after bleaching). NZ contents of the powders are obtained from UV visible measurements of the solution after the dissolution of the powders. Surface atomic percentages ($C_{organic}/C_{organic+inorganic}$) are obtained from XPS analysis.

Samples / NZ content		$ACC_{0.11NZ}$	$ACC_{0.53NZ}$	$Cal_{0.11NZ}$	$Cal_{0.53NZ}$	Bleached	
						$Cal_{0.11NZ}$	$Cal_{0.53NZ}$
Initial [NZ] in Na_2CO_3 solution (mM)		0.11	0.53	0.11	0.53	0.11	0.53
Initial NZ/Ca (%)		0.28	1.33	0.28	1.33	0.28	1.33
Amount of dissolved sample (mg)		60	20	50	20	100	50
$[Ca^{2+}]$ after $CaCO_3$ dissolution (mM)		12	4	10	4	20	12.5
[NZ] after $CaCO_3$ dissolution (mM)*		0.033	0.051	0.026	0.048	0.006	0.008
[NZ] associated with $CaCO_3$*	mM	0.109	0.51	0.10	0.48	0.01	0.03
	mol.%	0.27	1.28	0.25	1.2	0.03	0.08
	wt.%	0.51	2.40	0.47	2.26	0.05	0.15
Surface atomic percentage $C_{organic}/C_{organic+inorganic}$ (%)**		-	54	-	69	-	-

*Obtained from UV-visible measurements

**Surface atomic percentage obtained from XPS analysis, for pure ACC: 31% and for pure calcite: 38%.

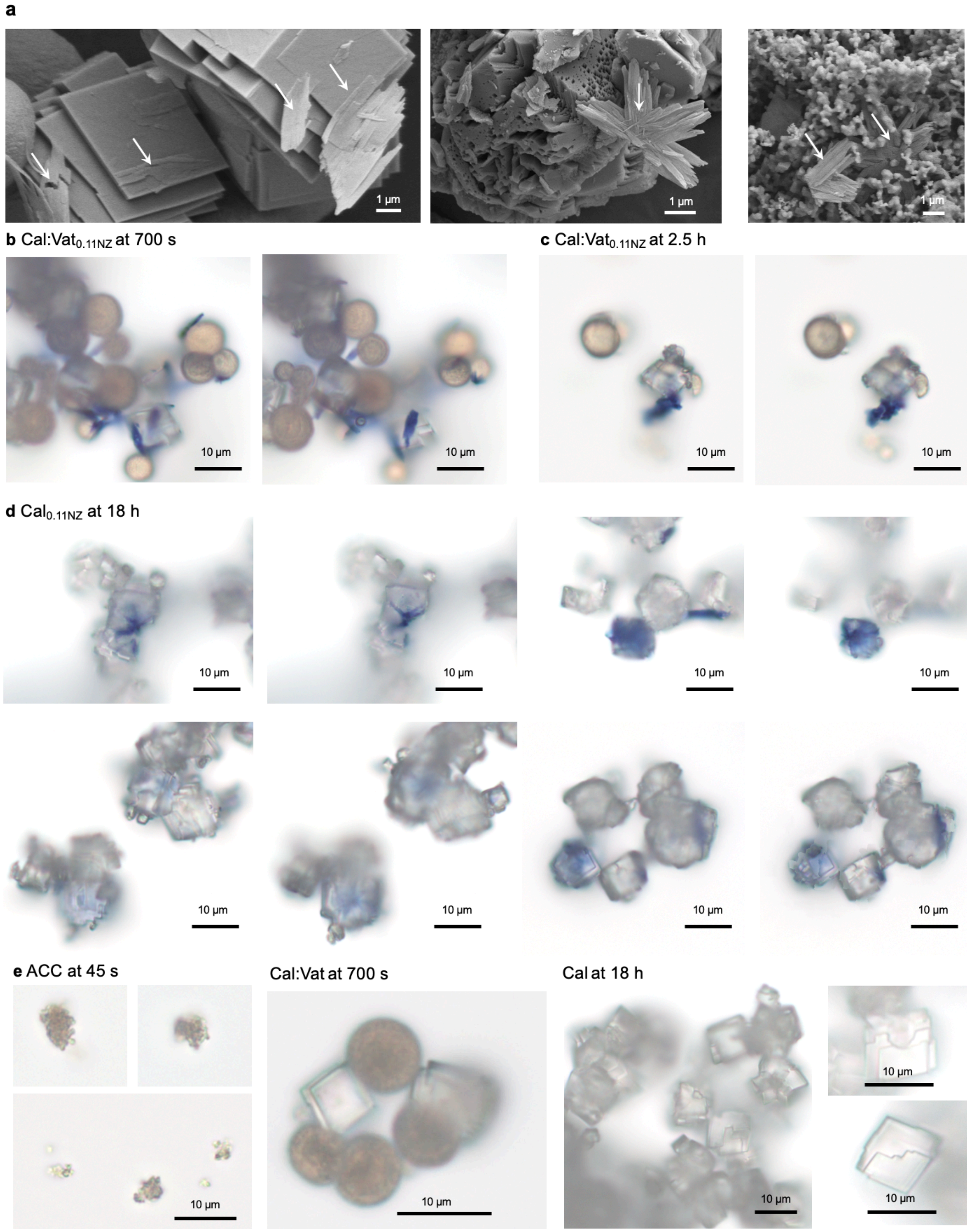

Supplementary Fig. 7. NZ visualization. a) FEG-SEM observations of crystallized NZ (indicated by the white arrows) at the surface of calcite crystals and ACC nanoparticles, transmission optical microscope images with two different focal planes of **b)** Cal:Vat$_{0.11NZ}$ at 700s, **c)** Cal:Vat$_{0.11NZ}$ at 2.5h, **d)** Cal$_{0.11NZ}$ at 18h and **e)** transmission optical microscope images of ACC, Cal:Vat and Cal samples.

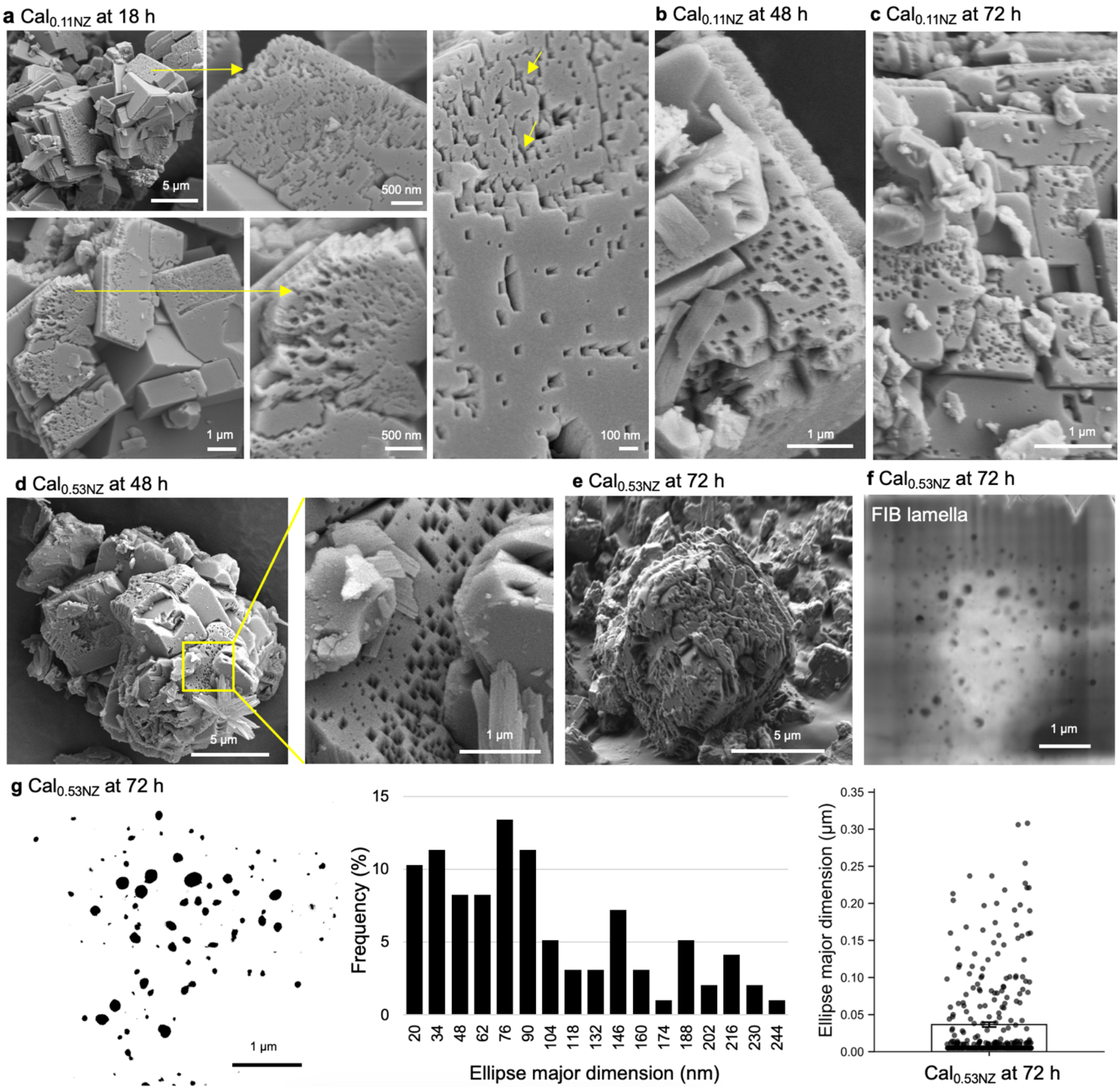

Supplementary Fig. 8. External and internal pores analyses. SEM micrographs of **a)** $Cal_{0.11NZ}$ after 18 h synthesis, the yellow arrows show crevasses at the surface of the crystals, **b-c)** $Cal_{0.11NZ}$ after 48 and 72 h synthesis, **d)** $Cal_{0.53NZ}$ after 48. h synthesis. At [NZ] = 0.53 mM, the rhombohedral pits are similar to the ones at [NZ] = 0.11 mM in terms of shape and size but the overall calcite crystal shape is less rhombohedral and more isotropic with no defined facets, **e)** selected crystal of a bleached $Cal_{0.53NZ}$ sample after 72 h synthesis with **f)** its FIB lamella observed under SEM (in lens detector) and **g)** particle analysis of the internal pores with n=334 particles.

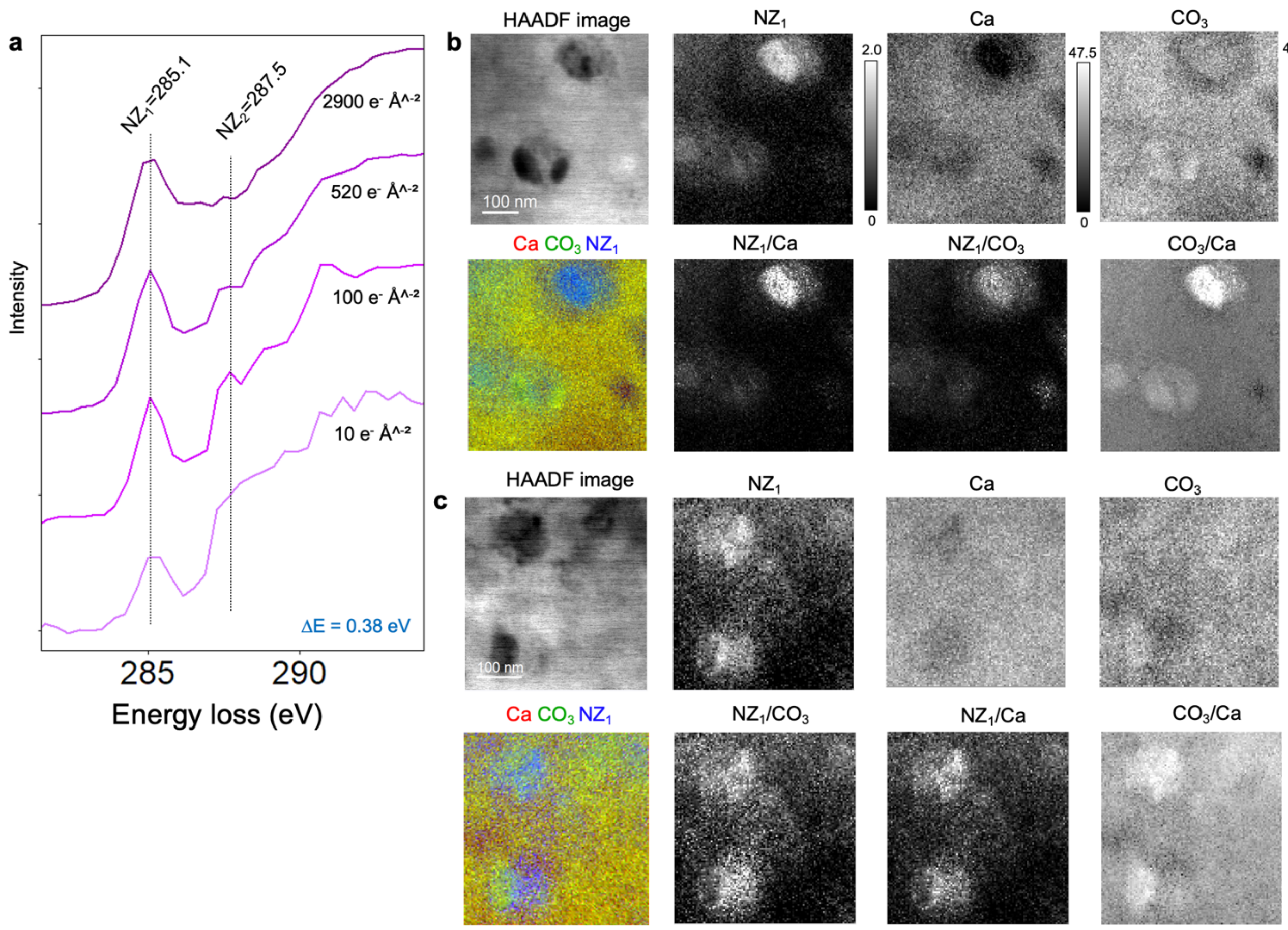

Supplementary Fig. 9. **EELS data. a)** C *K*-edge EELS spectra from NZ powder measured at a low energy dispersion ΔE of 0.38 eV per channel, required for simultaneous C and Ca analysis, and electron doses from 10 to 2900 e^{-} Å^$^{-2}$ averaged over an area of 500 x 500 nm^^{2}. Two peaks are detected at 285.1 eV and 287.5 eV denoted NZ_1 and NZ_2, respectively. As the dose increases (≥100 e^{-} Å^$^{-2}$), NZ degrades progressively, with peak broadening of the NZ_1 peak and the emergence of peak NZ_2, attributed to CO formation from NZ radiolysis[5]. At the lowest dose (10 e^{-} Å^$^{-2}$), spectral features remain undegraded[6], but the large pixel size (10 nm) and the low signal-to-noise ratio prevent chemical mapping. Therefore, the NZ_1 peak is used here to detect and map NZ because it dominates the NZ spectrum for all explored doses. In addition, the sample being a FIB lamella with NZ as the sole organic species, NZ_1 mapping is unambiguous and free from carbon film contributions. **b-c)** HAADF images of two selected areas from the FIB lamella with their corresponding EELS maps obtained by integrating the C *K*-edge of the NZ_1 peak, the Ca $L_{2,3}$-edge and the C *K*-edge of the CO_3 peak. The corresponding RGB composite images combining the spatial distributions of Ca (in red), CO_3 (in green) and NZ_1 (in blue) and the NZ_1/Ca, NZ_1/CO_3 and the CO_3/Ca maps are shown.

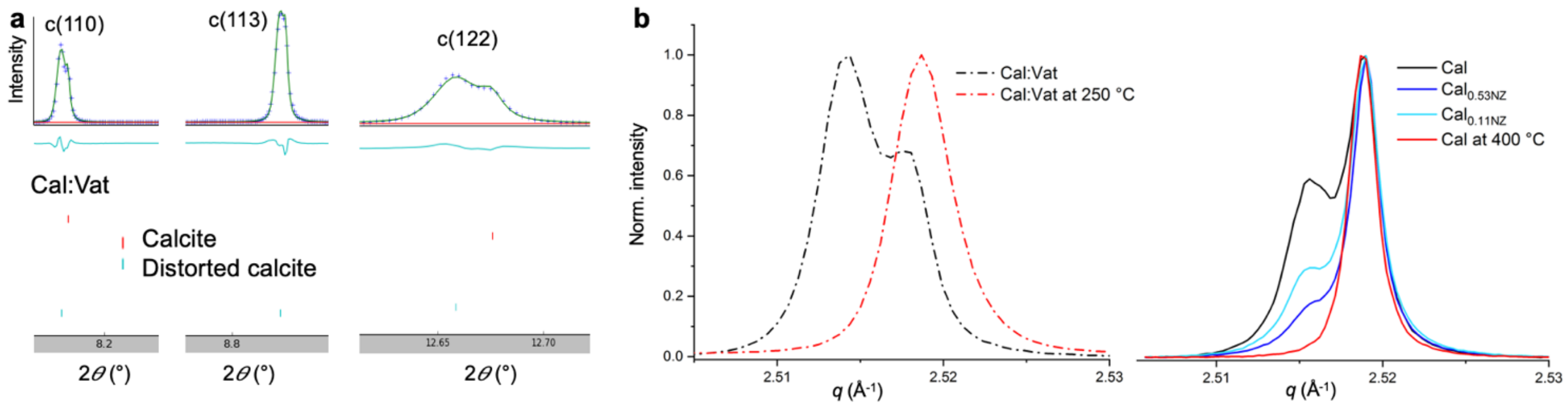

c

Samples	calcite		distorted calcite		vaterite		calcite : distorted calcite : vaterite (wt.%)	wR	GOF
	a	*c*	*a*	*c*	*a*	*c*			
Cal:Vat 700 s	-	-	4.99794	17.05753	4.12772	8.47306	0 : 59 : 41	9.758	11.37
Cal:Vat 700 s, at 250 °C	4.98977	17.08263	-	-	4.13130	8.46695	67 : 0 : 33	12.33	18.08
Cal:Vat$_{0.11NZ}$ 700 s	-	-	4.99218	17.0607	4.12687	8.47511	0 : 65 : 35	8.72	14.41
Cal:Vat$_{0.11NZ}$ 700 s, at 250 °C	4.98956	17.07929	-	-	4.13051	8.46705	73 : 0 : 27	11.74	14.79
Cal:Vat 2.5 h	4.99050	17.06430	4.99768	17.05310	4.12721	8.46912	24 : 64 : 12	7.483	6.65
Cal:Vat 2.5 h, at 250 °C	4.98954	17.07290	-	-	4.13323	8.46449	90 : 0 : 10	10.032	8.74
Cal:Vat$_{0.11NZ}$ 2.5 h	4.99038	17.06596	4.99715	17.05647	4.12662	8.47281	36 : 50 : 14	7.375	8.46
Cal:Vat$_{0.11NZ}$ 2.5 h, at 250 °C	4.98658	17.06935	-	-	4.13532	8.46540	95 : 0 : 5	11.492	9.22
Cal	4.98912	17.05617	4.99322	17.04786	-	-	54 : 46 : 0	6.574	2.86
Cal$_{0.53NZ}$	4.98914	17.05900	4.99646	17.05481	-	-	72 : 28 : 0	6.632	3.22
Cal$_{0.11NZ}$	4.98925	17.05868	4.99502	17.05134	-	-	67 : 33 : 0	6.574	2.86
Cal$_{0.11NZ}$ at 150 °C	4.98925	17.05886	4.99461	17.04803	-	-	66 : 34 : 0	7.552	2.92
Cal$_{0.11NZ}$ at 200 °C	4.98932	17.05873	4.99581	17.04707	-	-	65 : 35 : 0	9.436	3.8
Cal$_{0.11NZ}$ at 250 °C	4.98925	17.05938	4.99414	17.04946	-	-	65 : 35 : 0	8.318	3.14
Cal$_{0.11NZ}$ at 300 °C	4.98872	17.06002	-	-	-	-	100 : 0 : 0	8.79	3.66
Cal$_{0.11NZ}$ at 400 °C	4.98579	17.06074	-	-	-	-	100 : 0 : 0	9.42	4.07

d

Samples	Lattice distortions of distorted calcite with respect to calcite	
	Δa/a (%)	*Δc/c* (%)
Cal:Vat 2.5 h	0.14	-0.07
Cal:Vat$_{0.11NZ}$ 2.5 h	0.14	-0.06
Cal	0.08	-0.05
Cal$_{0.11NZ}$	0.12	-0.04
Cal$_{0.53NZ}$	0.15	-0.02

e

Samples	Calcite		Distorted calcite	
	Δa/a (%)	*Δc/c* (%)	*Δa/a* (%)	*Δc/c* (%)
Cal:Vat$_{0.11NZ}$ 700 s	-	-	-0.12	0.02
Cal:Vat$_{0.11NZ}$ 2.5 h	0.00	0.01	-0.01	0.02
Cal$_{0.11NZ}$	0.00	0.02	0.04	0.02
Cal$_{0.53NZ}$	0.00	0.02	0.07	0.04

Lattice distortions calculated with respect to Cal and Cal:Vat

Supplementary Fig. 10. Rietveld refinement analysis. a) fits of the HR-XRD profile for the Cal:Vat sample taken at 2.5 h of the reaction, **b)** (110) HR-XRD reflections of the calcite phase of Cal:Vat, Cal:Vat$_{0.11NZ}$ at 2.5 h before and after annealing at 250 °C, Cal before and after annealing at 400 °C as well as Cal$_{0.11NZ}$ and Cal$_{0.53NZ}$, **c)** results of Rietveld refinement of all samples, **d)** lattice distortions of the distorted nanodomains calculated with respect to the undistorted calcite nanodomains and **e)** lattice distortions of the calcite and distorted nanodomains calculated with respect to Cal:Vat and Cal.

a

Samples	Calcite				Distorted calcite			
	(110)		(202)		(110)		(202)	
	Cryst. size	Error	Cryst. size	Error	Cryst. size	Error	Cryst. size	Error
Cal:Vat 700 s	-	-	-	-	0.205	0.008	0.288	0.030
Cal:Vat 700 s, at 250 °C	0.231	0.009	0.271	0.025	-	-	-	-
Cal:Vat$_{0.11NZ}$ 700 s	-	-	-	-	0.226	0.006	0.291	0.023
Cal:Vat$_{0.11NZ}$ 700 s, at 250 °C	0.234	0.007	0.260	0.017	-	-	-	-
Cal:Vat 2.5 h	0.355	0.018	0.364	0.050	0.375	0.014	1.755	0.222
Cal:Vat 2.5 h, at 250 °C	0.309	0.016	0.324	0.020	-	-	-	-
Cal:Vat$_{0.11NZ}$ 2.5 h	0.336	0.011	0.350	0.018	0.372	0.019	1.404	0.171
Cal:Vat$_{0.11NZ}$ 2.5 h, at 250 °C	0.325	0.011	0.329	0.013	-	-	-	-
Cal	0.407	0.007	0.407	0.005	0.513	0.028	1.976	0.438
Cal$_{0.53NZ}$	0.357	0.002	0.383	0.002	0.366	0.033	0.602	0.077
Cal$_{0.11NZ}$	0.326	0.003	0.345	0.002	0.427	0.029	0.848	0.084
Cal$_{0.11NZ}$ at 150 °C	0.326	0.003	0.350	0.002	0.486	0.043	0.893	0.096
Cal$_{0.11NZ}$ at 200 °C	0.324	0.003	0.349	0.002	0.472	0.037	0.905	0.116
Cal$_{0.11NZ}$ at 250 °C	0.326	0.003	0.346	0.003	0.524	0.047	1.089	0.236
Cal$_{0.11NZ}$ at 300 °C	0.261	0.003	0.282	0.003	-	-	-	-
Cal$_{0.11NZ}$ at 400 °C	0.303	0.003	0.338	0.003	-	-	-	-

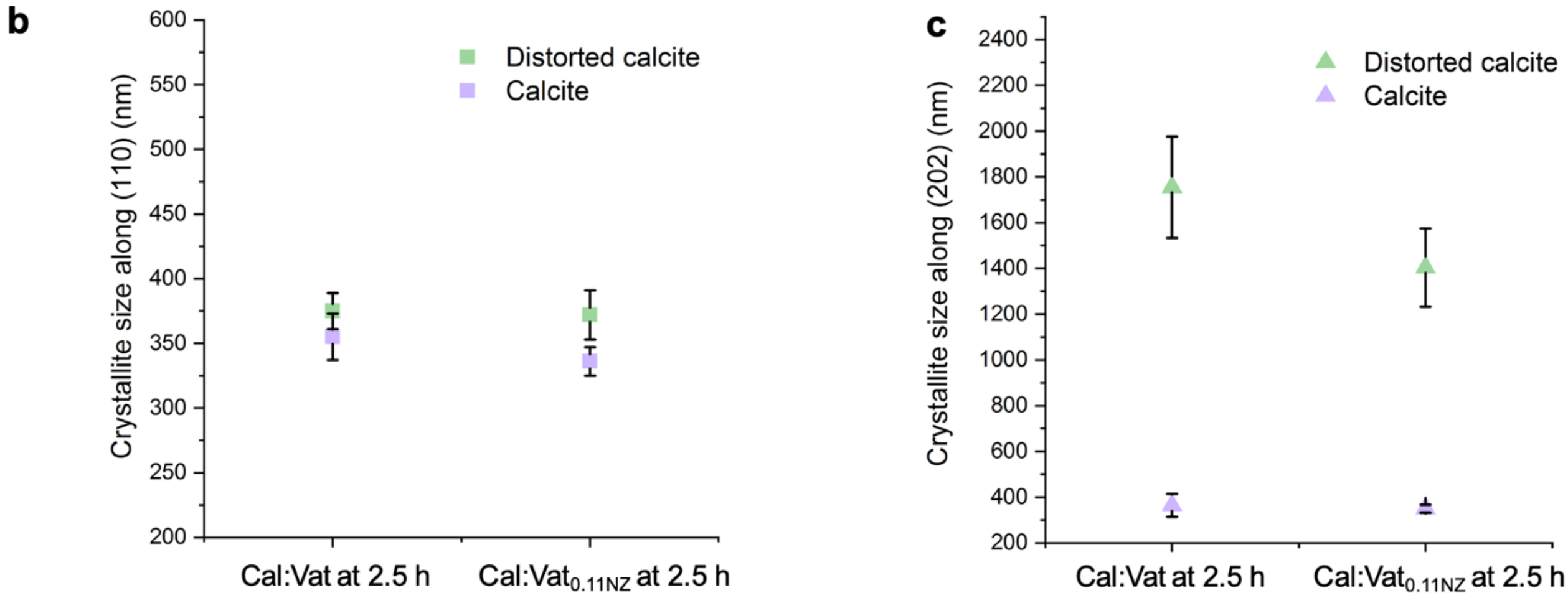

Supplementary Fig. 11. Crystallite size analyses. a) Crystallite sizes (µm) and error (µm) of the calcite and distorted calcite nanodomains along the (110) and (202) directions obtained after peak fitting using a Voigt function according to Pokroy and Zolotoyabko, 2006 [7] and crystallite sizes of the calcite and distorted calcite nanodomains along **b)** the (110) and **c)** the (202) directions of the Cal:Vat and Cal:Vat$_{0.11NZ}$ samples at 2.5h. The error bars correspond to the standard error of the fitting with the Voigt function.

Supplementary references

1. Savko, M. *et al.* Ground and excited state properties of naphthazarin: Absorption spectroscopy and theoretical modeling study. *J. Mol. Struct.-Theochem* **803**, 79–87 (2007).

2. Cavalcanti, G. R. S. *et al.* Inorganic-organic hybrid pigments based on carminic acid and clay minerals. *Dyes Pigments* **190**, 109306 (2021).

3. Trigueiro, P. *et al.* When anthraquinone dyes meet pillared montmorillonite: Stability or fading upon exposure to light? *Dyes Pigments* **159**, 384–394 (2018).

4. Chu, D. H. *et al.* CO2 mineralization into different polymorphs of CaCO3 using an aqueous-CO2 system. *RSC Adv.* **3**, 21722–21729 (2013).

5. Chaupard, M. Electron spectromicroscopy : from organic-inorganic drug nanocarriers to biological systems. (Université Paris-Saclay, 2023).

6. Chaupard, M. *et al.* Nanoscale Multimodal Analysis of Sensitive Nanomaterials by Monochromated STEM-EELS in Low-Dose and Cryogenic Conditions. *ACS Nano* **17**, 3452–3464 (2023).

7. Pokroy, B., Fitch, A. & Zolotoyabko, E. The Microstructure of Biogenic Calcite: A View by High-Resolution Sync hrotron Powder Diffraction. *Adv. Mater.* **18**, 2363–2368 (2006).
