# Supplementary material for: Halochromic modulation of amorphous calcium carbonate crystallization driven by pH-responsive bioinspired pigments
Source: Nat Commun. 2026 Aug 3;17:9378. doi: 10.1038/s41467-026-75888-8 (PMC13534625; doi:10.1038/s41467-026-75888-8)
Supplement: Supplementary file 2 [file 41467_2026_75888_MOESM2_ESM.pdf]

nature portfolio

Corresponding author(s): Marie Albéric

Last updated by author(s): 2026 - 04 - 15

Reporting Summary

Nature Portfolio wishes to improve the reproducibility of the work that we publish. This form provides structure for consistency and transparency in reporting. For further information on Nature Portfolio policies, see our Editorial Policies and the Editorial Policy Checklist.

Statistics

For all statistical analyses, confirm that the following items are present in the figure legend, table legend, main text, or Methods section.

n/a	Confirmed	
☐	☒	The exact sample size (*n*) for each experimental group/condition, given as a discrete number and unit of measurement
☒	☐	A statement on whether measurements were taken from distinct samples or whether the same sample was measured repeatedly
☒	☐	The statistical test(s) used AND whether they are one- or two-sided<br>*Only common tests should be described solely by name; describe more complex techniques in the Methods section.*
☒	☐	A description of all covariates tested
☒	☐	A description of any assumptions or corrections, such as tests of normality and adjustment for multiple comparisons
☐	☒	A full description of the statistical parameters including central tendency (e.g. means) or other basic estimates (e.g. regression coefficient) AND variation (e.g. standard deviation) or associated estimates of uncertainty (e.g. confidence intervals)
☒	☐	For null hypothesis testing, the test statistic (e.g. *F*, *t*, *r*) with confidence intervals, effect sizes, degrees of freedom and *P* value noted<br>*Give P values as exact values whenever suitable.*
☒	☐	For Bayesian analysis, information on the choice of priors and Markov chain Monte Carlo settings
☒	☐	For hierarchical and complex designs, identification of the appropriate level for tests and full reporting of outcomes
☒	☐	Estimates of effect sizes (e.g. Cohen's *d*, Pearson's *r*), indicating how they were calculated

Our web collection on statistics for biologists contains articles on many of the points above.

Software and code

Policy information about availability of computer code

Data collection	tiamo 2.5, Zen core v3.8 , Zen core v2.7 and v3.8, Cary Win UV scan Application v 6.2.0.1588, Avantage© v5.9931, PC_SEM.3.1, COMMANDER v8.61, Bruker Topspin software 3.6.3 and 3.7, Nion Swift 16.9.1
Data analysis	tiamo 2.5, Zen core v3.8 , Zen core v2.7, Cary Win UV scan Application v 6.2.0.1588, Avantage© v5.9925, PC_SEM.3.1, COMMANDER v8.61, Bruker Topspin software 3.6.3, Bruker Topspin software 3.7, Nion Swift, Gatan's Digital Micrograph 3.62.4986.0

For manuscripts utilizing custom algorithms or software that are central to the research but not yet described in published literature, software must be made available to editors and reviewers. We strongly encourage code deposition in a community repository (e.g. GitHub). See the Nature Portfolio guidelines for submitting code & software for further information.

Data

Policy information about availability of data

All manuscripts must include a data availability statement. This statement should provide the following information, where applicable:

- Accession codes, unique identifiers, or web links for publicly available datasets
- A description of any restrictions on data availability
- For clinical datasets or third party data, please ensure that the statement adheres to our policy

The data that support the finding of this study are available from Zenodo92 and from the corresponding authors upon request.

Research involving human participants, their data, or biological material

Policy information about studies with human participants or human data. See also policy information about sex, gender (identity/presentation), and sexual orientation and race, ethnicity and racism.

Reporting on sex and gender	n/a
Reporting on race, ethnicity, or other socially relevant groupings	n/a
Population characteristics	n/a
Recruitment	n/a
Ethics oversight	n/a

Note that full information on the approval of the study protocol must also be provided in the manuscript.

Field-specific reporting

Please select the one below that is the best fit for your research. If you are not sure, read the appropriate sections before making your selection.

[x] Life sciences [ ] Behavioural & social sciences [ ] Ecological, evolutionary & environmental sciences

For a reference copy of the document with all sections, see nature.com/documents/nr-reporting-summary-flat.pdf

Life sciences study design

All studies must disclose on these points even when the disclosure is negative.

Sample size	In total 134 samples were prepared, i.e. each sample was replicated about 10 times. No sample size calculation was performed. It was chosen as a function of the number of characterization techniques used and depending on how many time we could repeat the measurements according to the equipment avaibility and the duration of the analyses. The sample size seems sufficient because all replicates showed no significant variation after characterization.
Data exclusions	No data was excluded
Replication	pH and Ca measurements were repeated about 10 times, microscope images were aquired on about 20 crystals, spectroscopy (UV-visible, NMR) and xrd diffraction techniques result in an average measurements of several mg of powders, therefore providing good statistics of the bulk samples. About 20 EELS spectra were aquired whitin the sample to have a good overview. All replications were successful.
Randomization	This is not relevant to our study since the samples were not allocated into experimental groups
Blinding	This is not relevant to our study since there was no group allocation

Reporting for specific materials, systems and methods

We require information from authors about some types of materials, experimental systems and methods used in many studies. Here, indicate whether each material, system or method listed is relevant to your study. If you are not sure if a list item applies to your research, read the appropriate section before selecting a response.

Materials & experimental systems

n/a	Involved in the study	
[x]	[ ]	Antibodies
[x]	[ ]	Eukaryotic cell lines
[x]	[ ]	Palaeontology and archaeology
[x]	[ ]	Animals and other organisms
[x]	[ ]	Clinical data
[x]	[ ]	Dual use research of concern
[x]	[ ]	Plants

Methods

n/a	Involved in the study	
[x]	[ ]	ChIP-seq
[x]	[ ]	Flow cytometry
[x]	[ ]	MRI-based neuroimaging

Plants

Seed stocks	n/a
Novel plant genotypes	n/a
Authentication	n/a
