# Supplementary material for: Halochromic modulation of amorphous calcium carbonate crystallization driven by pH-responsive bioinspired pigments
Source: Nat Commun. 2026 Aug 3;17:9378. doi: 10.1038/s41467-026-75888-8 (PMC13534625; doi:10.1038/s41467-026-75888-8)
Supplement: Supplementary file 3 [file 41467_2026_75888_MOESM3_ESM.pdf]

nature portfolio

Peer Review File

Halochromic modulation of amorphous calcium carbonate crystallization driven by pH-responsive bioinspired pigments

Corresponding Author: Dr Marie Albéric

This file contains all reviewer reports in order by version, followed by all author rebuttals in order by version.

Attachments originally included by the reviewers as part of their assessment can be found at the end of this file.

Version 0:

Reviewer comments:

Reviewer #1

(Remarks to the Author)
The report of Sardhalia et al. describes the incorporation of a bio-inspired pH-sensitive dye into calcium carbonate. It builds on earlier research on sea urchin pigment molecules, adapting a synthesis route via amorphous transient precursors. In short, it shows the incorporation of the dye dihydroxy-naphthoquinone (NZ) into intermediate amorphous calcium carbonate, which transforms mainly to NZ-loaden calcite.
The study employs a broad range of methods to demonstrate the incorporation of NZ, how it impacts precipitation and crystallization, and rationalize the observed behavior, i.e., the color change during the process due to the halochromic NZ dye. With this, the authors produce chalk-based pigments with enhanced temperature and claimed pH stability (see comment below). The paper is of high scientific standard, with state-of-the-art methods employed. The presentation is scholarly and detailed; it would, in fact, benefit from a more concise style. The figures are prepared in a suitable fashion. Overall, the study can be accepted for publication after addressing the pending issues below.
- You determine two calcite phases. Give some background information: was it already reported elsewhere (references?), and how does it differ the “metastable” differ from the “stable” calcite? What is its origin?
- You apply ACC-NZ as a textile finish and assign the observed color stability to the ACC matrix, providing color stability. Why does ACC not dissolve under these harsh conditions? At pH 3, calcium carbonate in whatever phase should have already been decomposed completely. I assume it is not the ACC matrix that provide the color stability but the complexation of Ca to NZ, stabilizing its deprotonated variants in acidic media. I suggest a suitable control experiment with the additional presence of Ca.
- For those unfamiliar with DRS, one should introduce parameters L*, a*, and b* in the context of the CIE color space. Consider explaining the meaning in the experimental part, along with references.

Reviewer #2

(Remarks to the Author)
An in vitro study of color associated with calcium carbonate that highlights the role of pH and speciation on likely interactions between the 'color inducing agent and the mineral phase. The molecule chosen is a proxy for molecules found in nature that are not readily available in sufficient quantity to perform detailed fundamental studies- so the approach is justifiable.
The work contains a plethora of experimental approaches for all aspects of the research and reaches some conclusions concerning the interaction of the various mineralising species and the chosen dye molecule in its different protonated states. It concludes with a short section where the dye is used to colour cloth. The research appears to have been performed carefully with the authors having access to a wide range of state of the art instrumentation and many collaborators involved. This reviewer would have liked to have seen more comparison from the start to the structure and likely behaviour of at least one of the 'dye' molecules found in nature, including from a structural perspective. I would have liked to have seen more emphasis on the techniques and their results (for example better annotated NMR results- there are differences in the data, present in the figures and not accounted for in the text that show directly the interactions (if they really do so!) as opposed to the lengthy discussion of some of the solution data- much of this belongs in the SI and is supportive - perhaps done 'first' but does not add to the story. Data on the role of water, though this seems to play a key role in the interactions/ stabilisation- the data does not seem to support this. The role of the different calcite phases- metastable/ or other- more should be made of

this- particularly as this is largely a manuscript about the mechanism of formation of a material in the presence of a dye molecule.
I would recommend the authors rethink their storyline and ensure that all figures in the main paper relate to the key findings alone. The microscopy images, at least for this referee are not useful in that it is virtually impossible to 'see' the annotations aside from arrows. There are points in the MS where conclusions are drawn but the data not findable until later-an annotated file is provided to assist for specific queries. Towards the end there are numerous small errors in word endings that need to be corrected.

Reviewer #3

(Remarks to the Author)
See attached.

Version 1:

Reviewer comments:

Reviewer #1

(Remarks to the Author)
The revision has addressed several points raised by this reviewer, and the manuscript has improved substantially through reorganization. The reason for the remarkable acid stability of the colored textiles, however, remains unresolved, as no control experiments were performed. The additional data (Supplementary Figure 11) also do not clarify this issue, particularly since compositional analyses of the µm-sized patches (e.g., EDX/EDS) are missing. Given that this part of the manuscript is now framed more as an outlook on potential applications of these pigments, this may be acceptable. In its present form, the manuscript can be accepted for publication, provided one issue is clarified: the term “halochromic crystallization pathway” suggests the discovery of a novel crystallization mechanism in which halochromy directs crystallization. In reality, the work shows how crystallization conditions influence the halochromic behavior of a dye. The phrasing should therefore be adjusted to avoid overselling, as similar crystallization pathways have been reported previously.

Reviewer #2

(Remarks to the Author)
This is a much improved version of the MS. Referencing much better, figures and legends much better and the front part of the MS hangs together very well. It makes an interesting story to read.
To improve- the abstract needs to be rewritten to remove any ambiguity (the writing for this is much weaker than that for the conclusions, for example).
Line 141 a percentage is not a ratio- please amend
Line 378- I think you mean Figure S9 and not as written.
I would stop the paper after the presented scheme- conclusions could remain the same.
I recommend completely removing the section on dyeing of materials- in my opinion this takes away from the main thrust of the paper. The material should be published separately.

Reviewer #3

(Remarks to the Author)
The authors have made substantial changes. Thank you, for example, for explaining the use of NZ vs PHNQs and for using labels for the different materials combinations. Despite the notable effort, I still find this manuscript to be too dense and the conclusions not fully well-reasoned. The authors provide detailed descriptions of their data; however, it is still unclear which pieces of data are most relevant to their story. Especially for Nature Communications, the article ought to be crisp, logical, and profound. I would recommend resubmission after the authors have a chance to thoroughly analyze their data, clarify and substantiate their hypotheses, refine their story, and revamp their figures for clarity. I do appreciate the authors’ efforts in revision, but a more serious revision is needed to make the paper suitable for Nat Comm.
I hope the following additional comments can be helpful.

1. It seems to me that the main storyline ought to be centered on the authors’ findings regarding “....naphthazarin modifies the kinetic and thermodynamic formation of the crystalline $CaCO_3$ polymorphs…” If this is indeed the case, please explicitly address both the kinetics and thermodynamics and how NZ modifies each of these. As is, this is not clearly and logically drawn out. Several of the references cited in this submission do a great job of distilling similarly complicated data and coherently communicating insights with appropriate messaging and clear, helpful figures. I recommend using those as stylistic guides. One example is ref 43 on ACC formation and transformation (and refs 10-12 therein on the crystallization and the role of inclusions such as magnesium ions, sulfate, etc.). Another example is ref 57 on micelle incorporation into $CaCO_3$ and formation of occlusions.
2. Response to reviewer 1 regarding crystallography (metastable vs distorted): the authors make unsubstantiated claims and only correct them/ look more deeply when questioned by reviewers. While being open to revising their hypothesis is excellent, this raises a red flag for me. I think they need more time analyzing their data & previous literature to ensure the logic of their conclusions.

3. The response to reviewer 1 regarding dissolution of calcium carbonate raises questions regarding the necessity of the NZ/CaCO3 combination. Doesn't the dissolution of CaCO3 discount the authors' claim that "the resulting hybrid pigments exhibit enhanced color stability in response to changes in pH, as demonstrated by textile finishing assays" moot altogether? Rather than acting as a stabilizer for NZ, it seems that CaCO3 is a carrier for delivering NZ to the textile, and it is really the NZ-textile association that stabilizes the NZ.
4. Thank you for updating the naming convention and including a table. I recommend keeping X#NZ in line with Cal and KBr for the physical mixtures, rather than making it a subscript.
5. Abstract: 'primarily' → 'often' [reason: color can also arise from geometry alone, in the form of layering or patterning of biominerals, i.e. structural color]
6. Intro: 'fascinates with' is technically grammatically correct, but reads unnaturally. OK to keep, of course, but I suggest to replace with something like "Marline life is fascinating for its ability…"
7. Line 92: What is meant by "pigmentation mechanism of biominerals?" Does this refer to the biological mechanisms of molecular pigment incorporation into biominerals, or something else?
8. Line 153: "color changes" suggest dynamics; I suggest replacing with "color differences." This is relevant to other places within the manuscript as the word choice gives the impression of dynamic color changes as opposed to static color shifts.
9. Line 203: "indicating that the solution's color can indeed be determined from its spectrum." Seems like a circular statement - when does the spectrum not determine the color?
10. The figures and captions can still be substantially improved - don't make the reader guess what is intended. For example, in Fig 2a, the vertical lines - likely absorption maxima of the corresponding molecule? - are not defined in the caption. The schematic inset in Fig 2b isn't particularly helpful. Fig 3a should say "from top to bottom" rather than the other way around. I am not really sure what to make of Figure 5a. The schematics seem to show the final morphologies than the formation processes.
11. Line 378: should be "Fig S8"?
12. Lines 367-372: Regarding the formation of pores, the authors suggest that stacking of naphthazarin could result in pore formation but also that this could be a result of pitting/etching due to acid/base conditions; have the authors run a control experiment that can clarify this? Does the growth of these pores over time result in any structural colour? Additionally, the discussion around the SEM, optical images and EELS used for pore analysis could be more succinct and less disjoint.

Version 2:

Reviewer comments:

Reviewer #1

(Remarks to the Author)
The revised manuscript sufficiently addresses all remaining issues raised by this reviewer who now can recommend acceptance of the manuscript for publication.

Reviewer #2

(Remarks to the Author)
In addition to previous comments (I will not repeat them here) I am happy that the authors have addressed this referees' queries. The new MS is significantly improved. the story is clear and well supported by a range of relevant data. The topic is of interest to the biomineralization community as well as to those interested in 'colour'!
I believe it is now suitable for publication.

Reviewer #3

(Remarks to the Author)
The authors have substantially improved the manuscript through reorganization and curation of the results, clarifying their explanations, and revising the figures. Fig. 5 schematic is much more comprehensible and informative now. I believe this manuscript is now ready for publication in Nature Communications.

Open Access This Peer Review File is licensed under a Creative Commons Attribution 4.0 International License, which permits use, sharing, adaptation, distribution and reproduction in any medium or format, as long as you give appropriate credit to the original author(s) and the source, provide a link to the Creative Commons license, and indicate if changes were made.
In cases where reviewers are anonymous, credit should be given to 'Anonymous Referee' and the source.
The images or other third party material in this Peer Review File are included in the article's Creative Commons license, unless indicated otherwise in a credit line to the material. If material is not included in the article's Creative Commons license and your intended use is not permitted by statutory regulation or exceeds the permitted use, you will need to obtain permission directly from the copyright holder.
To view a copy of this license, visit https://creativecommons.org/licenses/by/4.0/

We would like to sincerely thank the reviewers for their very constructive comments and suggestions, which have significantly helped us improve the clarity and quality of our manuscript. We address each of the comments below, where the reviewer comments are in black, and our responses are in blue. Changes in the manuscript are also marked in blue.

Reviewer #1 (Remarks to the Author):

The report of Sardhalia et al. describes the incorporation of a bio-inspired pH-sensitive dye into calcium carbonate. It builds on earlier research on sea urchin pigment molecules, adapting a synthesis route via amorphous transient precursors. In short, it shows the incorporation of the dye dihydroxy-naphthoquinone (NZ) into intermediate amorphous calcium carbonate, which transforms mainly to NZ-loaden calcite. The study employs a broad range of methods to demonstrate the incorporation of NZ, how it impacts precipitation and crystallization, and rationalize the observed behavior, i.e., the color change during the process due to the halochromic NZ dye. With this, the authors produce chalk-based pigments with enhanced temperature and claimed pH stability (see comment below). The paper is of high scientific standard, with state-of-the-art methods employed. The presentation is scholarly and detailed; it would, in fact, benefit from a more concise style. The figures are prepared in a suitable fashion. Overall, the study can be accepted for publication after addressing the pending issues below.

1) You determine two calcite phases. Give some background information: was it already reported elsewhere (references?), and how does the "metastable" differ from the "stable" calcite? What is its origin?

Thanks to the high-resolution XRD measurement performed at the ID22 beamline of ESRF, we evidenced a splitting of some calcite reflections that we first attributed to the presence of two calcite phases: one "stable" named "calcite-1" and one "metastable" phase called "calcite-2". Both phases are of rhombohedral symmetry and show only slightly different lattice parameters between them in the hexagonal setting of calcite. However, thanks to the comments of reviewers 1 and 2, a review of the literature, and the calculation of the lattice distortions of one phase with respect to the other (Figure SI10D), we now attribute this splitting to two distinct domains of calcite with varying degrees of distortion.

The previously named "calcite-1" phase, which has the closest lattice parameters to typical geological and synthetic calcite, is now attributed to one domain that we call "calcite". The previously named "calcite-2", which is slightly distorted with respect to typical calcite, is now attributed to nanodomains called "distorted calcite". "Distorted calcite" shows in fact a very small contraction of the lattice along the c-axis (-0.02 to -0.07%) and a relatively small extension of the (ab) plane (0.08-0.15%) with respect to "calcite". For comparison, Mg occlusion within calcite induces higher distortions (Goldsmith 1961). These minor distortions between "distorted calcite" and "calcite", therefore, cannot be attributed to two distinct phases but instead to two domains with different levels of distortions within the calcite phase.

In addition, we could not find in the literature a calcite phase similar to what we previously named a "metastable calcite phase", i.e., with the same R3c space group, similar lattice parameters, and synthesized under identical experimental conditions (room temperature, atmospheric pressure, in solution) as in our study. Although different $CaCO_3$ metastable phases have already been reported, such as CCHH (Zou 2019), high-pressure polymorphs $CaCO_3$-II, -III, -IIIb, and -VI (Gavryushkin 2020), nanocrystalline monoclinic $CaCO_3$ (Nemeth 2018). Computational studies also predicted the presence of metastable $CaCO_3$ phases during the transformation of aragonite into calcite (Gavryushkin 2020, Bruno 2022), but also with different space groups and lattice parameters than the "calcite-2" phase identified in this study. However, nanodiscontinuities (Metzger 2014), nano-crystalline domains in single crystalline prisms (Mastropietro2017) also called internal nanocristallinity (Suzana 2024) or

lattice distortions across scales (Schoeppler2022) have been often reported in biogenic and synthetic calcitic systems. Suzanna et al. 2024 revealed multiple crystalline domains in calcite crystals synthesized by direct precipitation. These crystals are twinned and/or overgrown, exhibiting a similar shape to the ones observed by SEM in our study. It is therefore highly expected that different crystalline domains are also present in our samples.

Regarding the origin of these different domains, they are both present after the crystallization of ACC and persist after the transformation of vaterite into calcite, although their proportions vary. At the calcite-vaterite mixture stage, the "distorted calcite" domain is the most prominent, while at the calcite stage, it decreases from 64 wt.% to 46 wt.%, and the "calcite" domain increases from 24 wt.% to 54 wt.%.% (vaterite is 12 wt.%). Although we don't know if vaterite tends to directly transform into mainly "calcite" domains or first through "distorted calcite" domains, considering the different percentages, at least some "distorted calcite" domains directly convert to typical "calcite" ones in solution (although not completely after 3 days of synthesis). In addition, the distorted domains completely disappear after heating and convert into typical calcite domains, at 300 °C for 1h at the calcite stage and at 250 °C for 30 min at the vaterite:calcite stage. Therefore, the reorganization of the distorted domains is time-dependent and also thermally activated. The presence of distorted domains and their transformation occurs both in the presence and absence of NZ, therefore suggesting that they are possibly due to water molecules trapped within the calcite crystals during the crystallization of ACC into vaterite and the conversion of vaterite into calcite. Finally, the presence of NZ limits the presence of these distorted nano-domains, and thus may also limit the presence of water, as already suggested for other additives (Tobler et al. 2015).

According to these new considerations and this review of the literature, we revised lines 414-438 in the Results section and lines 486-495 in the Discussion section of the revised manuscript. Fig. 4 has also been updated, and the some of the following references have been added.

Bruno, M., et al., Calcite/Aragonite epitaxy: a computational study for understanding mollusk shell formation, J. Phys. Chem. C 2022, 126:6472.

Gavryushkin, P.N., et al., Metastable structures of CaCO3 and their role in transformation of calcite to aragonite and postaragonite, Cryst. Growth Des. 2020, 21:65.

Goldsmith, J.R., et al., Lattice constants of calcium-magnesium carbonates, The American mineralogist, 1961, 46:453.

Mastropietro, F., et al. Revealing crystalline domains in a mollusc shell single-crystalline prism, Nature materials 2017, 16:946.

Metzger, T.H., et al., Nanostructure of biogenic calcite and its modification under annealing: study by high-resolution x-ray diffraction and nanoindentation, Cryst. Growth Des. 2014, 14:5275.

Nemeth, P., et al. A nanocrystalline monoclinic CaCO3 precursor of metastable aragonite, Science Advances 2018, 4:eaau6178.

Schoeppler, V., et al., Untangling the mechanisms of lattice distortions in biogenic crystals across scales, Adv. Mater. 2022, 34:2200690.

Suzana, A. F., et al. visualizing the internal nanocrystallininty of calcite due to nonclassical crystallization by 3D coherent X-ray diffraction imaging, Adv. Mater. 2024 36:2310672.

Tobler, D.J., et al. Citrate effects on ACC structure, stability and crystallization, Adv. Funct. Mater. 2015, 25:3081.

Zou, Z., et al. A hydrated crystalline calcium carbonate phase: calcium carbonate hemihydrate, Science 2019, 363:396.

2a) You apply ACC-NZ as a textile finish and assign the observed color stability to the ACC matrix, providing color stability. Why does ACC not dissolve under these harsh conditions? At pH 3, calcium carbonate in whatever phase should have already been decomposed completely.

We agree that ACC nanoparticles or calcite crystals should dissolve at pH 3 even if they are interacting strongly with the silk fibers. To better explain this point, as well as the transformation of the ACC-NZ hybrid pigment and its interaction with the silk fibers during the various steps of the dyeing procedure, we further characterized the hybrid pigment and the dyed fabrics.
The hybrid pigment considered here consists of ACC nanoparticles produced in the presence of NZ by the vapor diffusion method in ethanol. Although this sample is less prone to crystallization than the one synthesized in water, it likely crystallizes during the preparation of the printing paste (grinding step). Indeed, when stored in a desiccator, such a sample begins to crystallize within 1.5 days and fully crystallizes into mainly calcite after one month, forming nanoparticles of approximately 20-50 nm in size that can aggregate into structures of a few micrometers. SEM images of such a sample and XRD profiles showing its crystallization are reported now in Supplementary Figure 11A-C and mentioned lines 540-541.
After screen-printing, the crystalline nanoparticles are expected to "fuse" with the silk fibers, which are composed mainly of silk fibroin, resulting in strong organic-inorganic interactions, as already evidenced when $CaCO_3$ waste oyster powder is used as a mordant during dyeing (Sumang et al., 2024). At this stage, naphthazarin is deprotonated, resulting in the observed blue shade of the fabrics, as shown in Supplementary Figure 11D.
After washing at basic pH and rinsing, SEM observations of the dyed fabrics reveal very localized 1-2 µm patches at the surface of the fibers (Supplementary Figure 11E), possibly $CaCO_3$, but which is not detected by FT-IR analyses (not shown). Indeed, the latter only shows typical silk fibroin spectra with the absence of $CaCO_3$ likely due to its low concentration (1 wt.% hybrid pigment). Nevertheless, these patches seem less apparent once the dyed fabric is subjected to acidic shading, suggesting that indeed $CaCO_3$ dissolution occurs.

These new characterizations are now displayed in Supplementary Figure 11, and Figure 5 is better detailed; it presents some schematics to show the different baths with their respective pHs at which the dyed fabrics are subjected. The text has been revised accordingly (lines 545-556) in order to report the transformation from ACC-NZ to calcite-NZ of the hybrid pigment and its further dissolution when subjected to acetic acid treatment during the dyeing procedure.

Sumang, R. et al. Investigating the potential of waste oyster shell as a sustainable bio-mordant in natural dyeing. Scientific reports, 2024, 14:25509.

2b) I assume it is not the ACC matrix that provide the color stability but the complexation of Ca to NZ, stabilizing its deprotonated variants in acidic media. I suggest a suitable control experiment with the additional presence of Ca.

We agree that after shading using acetic acid, the lavender blue color stability likely originates from the presence of stable Ca^{2+}-NZ^{2-} and Ca^{2+}-NZH^{-} complexes, as suggested in line 490 of the first version of the manuscript. Unfortunately, due to the designer's unavailability for dyeing silk with our hybrid pigment, we were unable to perform the control experiment suggested by this reviewer. This experiment would have involved the screen-printing of silk fibers with a printing paste composed of Ca^{2+} (e.g., $CaCl_2$) and NZ mixed in guar gum at a basic pH. NZ would have required to be previously dissolved in basic aqueous solution (instead of ethanol), in order to stabilize the NZ^{2-} species and possibly form Ca^{2+}-NZ^{2-} complexes as stable as the ones present in the hybrid pigment.
However, we know that the complexation of Ca^{2+} and NZ^{2-} in basic solution is reversible and not strong enough to stabilize NZ^{2-} when the pH is decreased (Figure 4E of the revised Supplementary file). This therefore indicates that undergoing a solid $CaCO_3$ phase, which associates with NZ, where stable Ca-

NZ complexes are formed, is essential to maintain the complexation and thus the color stability after screen-printing, even under acidic conditions when CO_3^{2-} decomposes. We thus expect that a printing paste composed of $CaCl_2$, NZ, and guar gum at basic pH would not have been sufficient to stabilize the lavender blue color, as the Ca^{2+}-NZ^{2-} complexes may not have been stable enough to prevent NZ^{2-} from protonating again at acid pH, which will then induce a reddish hue.

Therefore, we propose that indeed the color stability is due to the complexation of Ca to NZ, but that the stability of this complex is possible thanks to the precipitation of ACC-NZ beforehand. ACC-NZ further crystallizes and interacts with the silk fibers. This is in agreement with the fact that $CaCO_3$ used as a mordant enhances dye fixation (Sumang et *al.* 2024).

To summarize, the interest of using ACC-NZ as a hybrid pigment for dyeing textiles resides in its ability to form stable Ca^{2+}-NZ^{2-} complexes that can be easily stored and preserved in the form of a dried powder before the dyeing procedure. In addition, the likely CO_2 release that occurs during the shading step with acetic acid could potentially combine the advantages of the screen-printing technique and the supercritical CO_2 dyeing process (Goni *et al.,* 2021) for competitive dye fixing and color fastness in limited water processes.

According to these considerations, the main text has been modified lines 558-565. In addition, in the revised version, we moved this section, now called "potential applications," to the end of the manuscript in response to some concerns from Reviewer 2 about the relevance of this part to the main story; however, we still believe it is an important and interesting point to address.

Goni, M. et al., Supercritical CO2-assisted dyeing and functionalization of polymeric materials: A review of recent advances (2015-2020), Journal of CO_2 utilization 2021, 54:101760.

3) For those unfamiliar with DRS, one should introduce parameters L*, a*, and b* in the context of the CIE color space. Consider explaining the meaning in the experimental part, along with references.

We complemented the experimental part line 790-792 concerning the DRS measurements accordingly by explaining that L* a* b* is a uniform color space recommended by the Commission Internationale de l'Eclairage (CIE), where L*, a*, and b* correspond to the lightness, the red-green axis, and the yellow-blue axis, respectively (Luo et al. 2014). We also included the reference below as 119.

Luo, M. R. CIELAB. In Encyclopedia of Color Science and Technology; Luo, R., Ed.; Springer: Berlin, Heidelberg, 2014; pp 1–7.

Reviewer #2 (Remarks to the Author):

An in vitro study of color associated with calcium carbonate that highlights the role of pH and speciation on likely interactions between the 'color inducing agent and the mineral phase. The molecule chosen is a proxy for molecules found in nature that are not readily available in sufficient quantity to perform detailed fundamental studies- so the approach is justifiable. The work contains a plethora of experimental approaches for all aspects of the research and reaches some conclusions concerning the interaction of the various mineralising species and the chosen dye molecule in its different protonated states. It concludes with a short section where the dye is used to colour cloth. The research appears to have been performed carefully with the authors having access to a wide range of state-of-the-art instrumentation and many collaborators involved.

I. General comments

1) This reviewer would have liked to have seen more comparison from the start to the structure and likely behaviour of at least one of the 'dye' molecules found in nature, including from a structural perspective.

Indeed, Spinochrome A, which is a PHNQ found in sea urchins was only mentioned at line 495 of the first version of the manuscript. Therefore, as advised, we now mention spinochrome A and and compare it to naphthazarin (NZ) in terms of molecular structure and halochromic properties in the Introduction (lines 77 and 108-110). We also present the structure of Spinochrome A in Fig. 1a.
This comparison is based on the results we reported in a manuscript entitled “Pigment-Macromolecule Isolation Complexes from Sea Urchin Biomineral Waste for Coloring Materials” by *Ferreira et al.,* which was just accepted with major revisions in the Chemistry-Methods Journal. We submitted our revisions on the 12th of September, they are under consideration. This manuscript is cited in the revised version as reference 54.
Some results from this manuscript are summarized below:
1) Spinochrome A ($C_{12}O_7H_8$, 3-Acetyl-2,5,7,8-tetrahydoxy-1,4-naphthoquinone) is the main polyhydroxy-1,4-naphthoquinone (PHNQ) molecule found in purple *Paracentrotus lividus* sea urchin spines. Echinochrome A, Spinochrome B, C, and E are found in minor quantities (Figure A1).
2) Spinochrome A displays absorbance bands in the same range as the ones of NZ (Figure A2) and similar halochromic properties as NZ (Figure A3).
3) The purple color of the spines is likely due to the encapsulation of deprotonated spinochrome A within calcite.
4) Direct precipitation of $CaCO_3$ in the presence of spinochrome A leads to powder with purple color similar to that obtained for $CaCO_3$+NZ in the present study and with similar UV-visible spectra (Figure A4).
5) Like NZ, spinochrome A delays the transformation of vaterite into calcite (for similar concentrations).

We now mention 3) to 5) in the Discussion part line 442, lines 447-449 and lines 501-502.

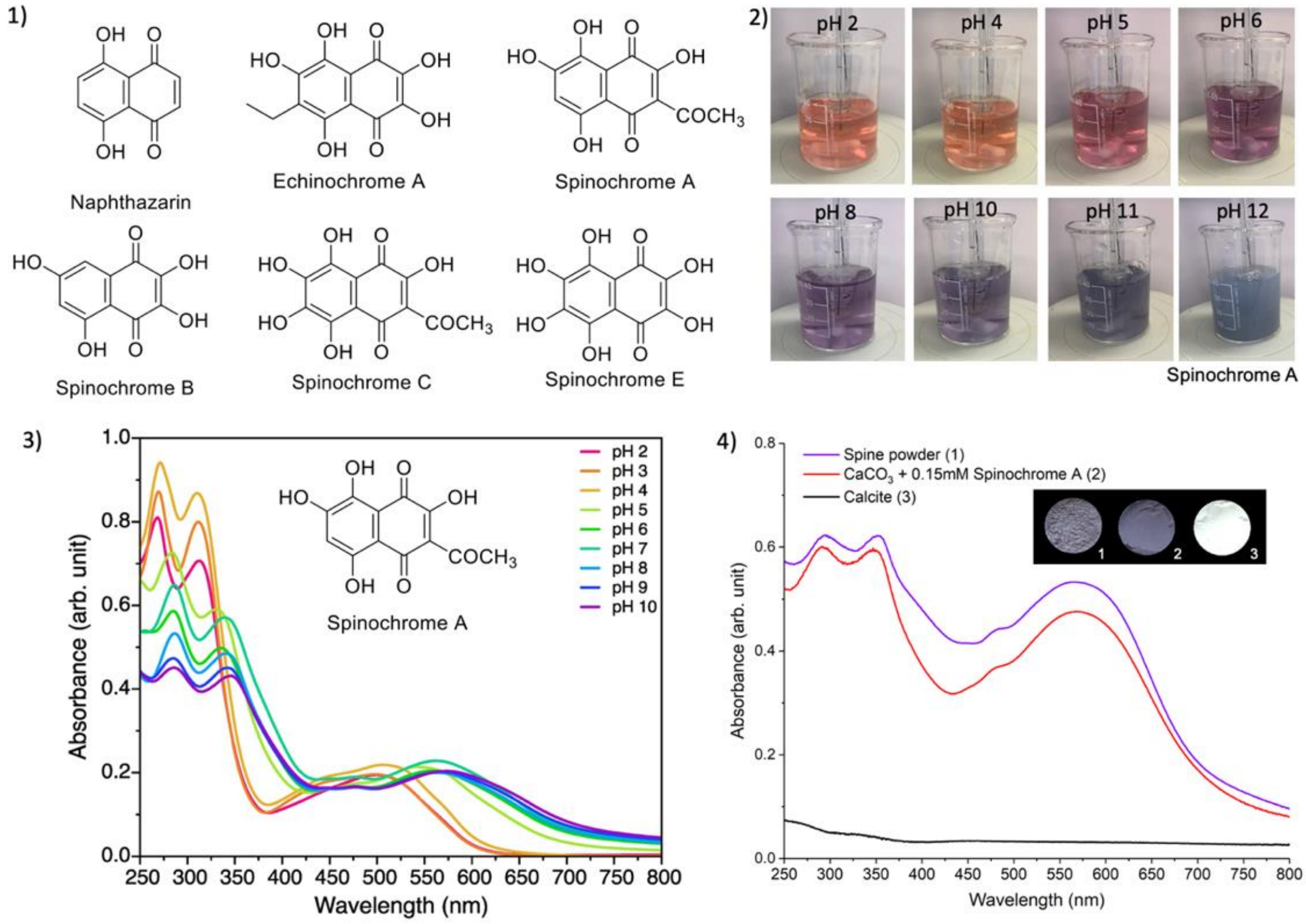

Figure A. 1) Comparison of the molecular structure of NZ and the five PHNQs found in P. lividus, 2) halochromic properties of spinochrome A extracted from purple *P. lividus* sea urchin spines and 3) respective UV-visible spectra according to pH and 4) Diffuse reflectance UV visible spectra and photographs of spine powder (1), calcite precipitated in the presence of 0.15mM Spinochrome A (2) and pure calcite (3).

2) I would have liked to have seen more emphasis on the techniques and their results (for example better annotated NMR results- there are differences in the data, present in the figures and not accounted for in the text that show directly the interactions (if they really do so!) …

Regarding the solid-state NMR results, the related paragraph has been significantly modified to emphasize the most important findings and clarify the discussion lines 240-276. The annotations of Figure 2c have been modified, and the three main "interactions", i.e., "spatial proximities", evidenced by solid-state NMR have also been annotated on the 2D ^{1}H-^{13}C HETCOR spectrum (Fig. 2d):

(i) The correlation between the ^{13}C and ^{1}H resonances from naphthazarin is now indicated by magenta dashed lines.

(ii)The correlation between the ^{13}C resonance from the CO_3 of calcite and the ^{1}H resonance from H_2O is now annotated by dashed black and light blue lines.

(ii) The correlation between H_2O and naphthazarin is now indicated by a dashed light blue line. The corresponding ^{13}C slice is also shown in light blue at the bottom of the 2D spectrum (Fig. 2d). Indeed, this slice displays the characteristic resonances of NZ^{2-} (in addition to the CO_3 resonance), which unambiguously evidences the spatial proximity of naphthazarin to water molecules.

For each of the decisive techniques, i.e., UV-Visible spectroscopy, NMR, STEM-EELS, and HR-XRD, we emphasized in the text their respective key findings by making clearer the contribution of each method to answer the main question of the story: explain the origin of the color changes and the formation of bio-inspired colored calcite crystals through amorphous precursors. All corresponding figures have also been better annotated.

3) … as opposed to the lengthy discussion of some of the solution data- much of this belongs in the SI and is supportive - perhaps done 'first' does not add to the story.

As advised, we moved the UV-visible data in solution, as well as their description and the determination of the pKas in the Supplementary file (Supplementary Fig. 4). We only kept in the main text the "in situ" UV-visible data, where we measured the supernatant at each step of the ACC synthesis and crystallization (Fig. 2b).

4) Data on the role of water, though this seems to play a key role in the interactions/ stabilisation- the data does not seem to support this.

These data concern our two-dimensional (2D) ^{1}H-^{13}C HETCOR NMR experiments of the Calcite+0.53mM_NZ. The obtained 2D ^{1}H-^{13}C HETCOR spectrum does suggest some spatial proximities of water and $CaCO_3$, as well as water and NZ. However, as stated above (comment 2), these data were not well described, and the related figure (now Fig. 2d) was not clearly annotated. We corrected these points (see answer to comment 2). We rewrote the NMR paragraph to show how the NMR data support the presence of water molecules between calcite and NZ, therefore suggesting that the interaction between calcite and NZ is mediated by water. Lines 271-276.

5) The role of the different calcite phases- metastable/ or other- more should be made of this- particularly as this is largely a manuscript about the mechanism of formation of a material in the presence of a dye molecule.

Based on the comment 1) of reviewer 1, we now consider different nanodomains with different levels of distortions rather than two calcite phases. As suggested by reviewers 1 and 2, we discuss in more detail the mechanism of formation of these nanodomains and the effect of the dye molecule (NZ) in comparison to the existing literature. In particular, the literature concerning the role of hydroxyl-rich molecules (similar to NZ) on vaterite/calcite polymorph selection. We revised lines 414-438 in the Results section and lines 486-495, 500-502 in the Discussion section of the revised manuscript. Fig. 4 has also been updated.

6) I would recommend the authors rethink their storyline and ensure that all figures in the main paper relate to the key findings alone.

As suggested by reviewer 3, we have drastically reorganized our manuscript and modified our storyline. Therefore, all figures in the main text have been modified, and the main text has been reorganized and partially rewritten (blue paragraphs). We ensure that the key findings are presented in the main text, and we have moved all supporting data to the supplementary information file, including UV-visible and NMR data in solution. We also removed the results concerning the crystallization of ACC into calcite induced by heating, specifically the UV-visible DRS data and the TGA-DSC results, which will be included in another paper. We decided to focus on crystallization in solution.

7) The microscopy images, at least for this referee are not useful in that it is virtually impossible to 'see' the annotations aside from arrows.

We modified this figure (now Fig. 3) and increased the size of the microscopy images as well as the annotations.

8) There are points in the MS where conclusions are drawn but the data not findable until later

After reorganizing the manuscript, we ensured that our conclusions were derived from the data described immediately preceding them.

II. Specific queries

We report here below the different annotations made by Reviewer 2 for specific queries in the Manuscript file highlighted in yellow and his/her comments are written in purple.

Line 133: "CaCO3+NZ samples display similar spectra than pure NZ powder"
now Line 169: similar to those of pure naphthazarin powder
Line 136: In more details,
now Line 172: In more detail,
Line 140: previously all attributed to
now Line 175: They are all attributed to
Line 144: the CaCO3+NZ samples also displays multiple
now Line 177: the colored $CaCO_3$ samples also display
Line 145: visible distinct bands but which are all red shifted
now Line 177: , but are all red-shifted.
Line 162: exhibit internal diffused violet
Now line 342: diffuse
Line 167: the lavender-bleu and
This sentence has been removed
Line 170: This color changes are due
Now line 154: These color changes
Line 182: Follows a pH drop to 9.8,
Now line 128: this sentence has been modified.
Line 187: Second, the two pKa of
Now line 189: pK_a values
Line 201: a pKa1 of 8.2 and a pKa2 of 10.3
Line 203: for pKa1 and 10.4-11.8 for pKa2
Line 204: is known to shift pKa values
Line 207: the pKa values
Line 222: pH=pKa2.
Line 223: pKa value.
Line 227: pH pKa2 , pKa
All pK_a spellings have been checked and replaced by pK_{a1} and pK_{a2}
Line 238: Similarly than
Now line 229: Similarly to
Line 246 : purplier
This sentence has been removed from the revised manuscript.

Line 251: lyophilized histidine powder by evidencing - relevance? make explicit
In this reference, the authors demonstrated that lyophilized histidine exhibits distinct and specific NMR chemical shifts, which are dependent on its speciation, i.e., its protonation state. Therefore, based on this study, we also lyophilized NZ at different pHs to control its protonation state. Thus, the NMR analyses of the ^{13}C chemical shifts allow us to identify the speciation of NZ in the Calcite+NZ sample. As this study is more relevant for its technical aspect, the corresponding reference is now cited in the Methods section (lines 716-717).

Line 264-270: just concentrate on the methods that TELL you what the states of NZ are- others to the SI
As advised, the NMR part has been largely rewritten to focus on the NZ protonation states and its interaction with calcite (see also answer to comment 2). Consequently, the NMR results in the presence of Ca^{2+} have been entirely moved to SI (Figure 5D).

Line 282-287: more should be made of the data in the figure- annotate to show
See answer to comment 2).

Line 296: nanoparticles neither on its local
Now line 137: neither the size…, nor … is…

Line 297: the water content of the samples and
This part has been removed as we have decided not to display the TGA-DSC data anymore, as it concerns the transformation of ACC in air. In contrast, this study is now focused on crystallization in solution.

Line 309-311: This is in agreement with our NMR results that suggest the presence of a water layer between NZ and $CO3^{2-}$ ions in the case of the Calcite+0.53mM_NZ sample. be explicit in where this data is
These NMR data refer to the NMR 2D map (now Fig. 2d). However, this sentence has been removed as we decided not to present the TGA-DSC data.

Line 326: reveal two calcite phases, state how these differ- why is this important to the mechanistic aspects?
Now line 415.
As stated in the answer to comment 1) of reviewer 1 and to comment 5) of this reviewer, we now consider two different levels of distortions of one calcite phase rather than two calcite phases. Indeed, after a review of the literature, we now propose that “calcite-1”, which has the closest lattice parameters to typical calcite, corresponds to one domain that we call “calcite” and that “calcite-2”, which is slightly distorted with respect to typical calcite, corresponds to nanodomains called “distorted calcite”. These two domains are essential to the mechanistic aspect because their proportions vary according to NZ concentrations and change over time during the transformation of vaterite into calcite. The distorted nanodomains decrease after vaterite conversion and disappear entirely after mild heating, suggesting they are due to the presence of trapped water.
In addition, NZ has an impact on the formation of these domains: limiting their occurrence maybe by reducing the occlusion of trapped water. We revised lines 414-438 in the Results section and lines 486-495, 500-502 in the Discussion section of the revised manuscript. Fig. 4 has also been updated.

Line 335-337. Overall, these results indicate that NZ promotes the formation of calcite over vaterite after ACC crystallization and the one of the stable calcite phase over the metastable one during vaterite conversion. surely there should be literature refs to include?
Yes, we included the literature concerning the effect of organic additives on $CaCO_3$ polymorph selection in the Discussion section lines 500-502.

Line 350-352: (Figure 3D, Fig. SI8). They show numerous 20-50 nm rhombohedral holes and labyrinthic crevasses. After two to three days of synthesis some of these rhombohedral holes increase in size up to 200 nm. These labyrinthic crevasses, Figure not convincing
Now line 360-362.
We agree that it was not easy to see on the provided SEM images what we named “labyrinthic crevasses”. We now report additional SEM images in Supplementary Fig. 8A, which provide a better display of these features, although the terminology may not be entirely appropriate. Therefore, in the revised version, we simply referred to these features as “crevasses” in agreement with Estroff et al. (2004) Org. Biomol. Chem., who evidenced similar features at the surface of calcite crystals grown in hydrogels.

Line 360: stacked NZ molecules, where is your evidence for this?
Now line 369-370.
We don't have evidence that shows that NZ molecules are possibly stacked. Here, it is merely a hypothesis, based on the planar molecular structure of NZ and how such molecules have been shown to often stack in the literature through pi-pi stacking (Cradwick, 1971; Pascard-Billy, 1962). Therefore, we reformulated this sentence to ensure that it is viewed only as a hypothesis.

Line 398-405: The results, summarized in Table SI3, show that ACC+0.11mM_NZ contains 0.49 wt.% NZ, ACC+0.53mM_NZ holds 2.4 wt.% NZ, Calcite+0.11mM_NZ, 0.47 wt.% NZ and Calcite+0.53mM_NZ, 2.26 wt.% NZ, which indicate that all samples capture most of the NZ molecules dissolved in the Na_2CO_3 solution
very difficult to compare without knowing actual amounts.
Now line 316-318. The actual amounts are indicated in mM in Table SI2. To ensure readers can refer to this table for more details, we have modified this sentence.

Line 407-409: Nevertheless, because Calcite+NZ and ACC+NZ comprise similar amount of total NZ and ACC+NZ shows less adsorbed NZ than calcite+NZ (XPS results), the former must encapsulate more than the latter. So what?
Now line 325-330. This suggests that after ACC crystallization via dissolution/reprecipitation, NZ is released in the solution before being incorporated in smaller amounts in the crystalline polymorphs. We added this point.

Line 432-434: Figure 3G and Fig. SI10 B-C -correct ratios- in figure/legend
Now, in Fig. 4c and Supplementary Fig. 9, we checked that the ratios were correct.

Line 435-437: Nevertheless, the spectra of some contiguous individual pixels (4x4 nm^2) of area 7 also reveal the presence of NZ (pixels c and d, Figure 3F), while others do not, suggesting a heterogenous distribution of NZ at the nano-scale within the bulk matrix. to say this you must define detection limits.
Now 404-405.
Although EELS measurements can be quantitative for the concentration of a single element after performing specific measurements, they are generally not a quantitative technique, making it difficult to assess detection limits. It would have been particularly difficult in the case of naphthazarin, which accounts for only one part of the carbon signal. Therefore, we cannot exclude the possibility that when not detected, NZ may be present, albeit in very low quantities. We therefore added this point lines 406-407. Nevertheless, we believe that our interpretation of the heterogeneous distribution of NZ remains valid because when NZ is detected, it must be present in a higher concentration than when it is not detected.

Line 452-454: Nevertheless, the higher lattice distortions measured for calcite-2 phase as compared to calcite-1, when NZ is present, indicate that NZ is preferentially incorporated within the metastable phase. but as one phase transforms to the other- what happens to the structure and to the NZ molecules?
Now line 429-430 and 437.
To answer this comment, we first calculated lattice distortions also at the calcite:vaterite stage, i.e., before the partial conversion of the distorted domains into typical calcite. We did so for the sample with 0.11 mM NZ only, as we don't have a measurement for the 0.53 mM sample. These values are reported in Supplementary Fig. 10E and mentioned in the main text lines 411, 430, and 438. They show the same trend as at the calcite stage, i.e., slightly higher lattice distortions in the presence of NZ for the distorted domains as compared to the typical calcite ones.

Second, we calculated lattice distortions of the typical calcite domains and the distorted ones at the calcite stage with respect to the calcite:vaterite stage in the absence and presence of 0.11mM NZ. We obtained similar lattice contraction in the absence and presence of 0.11mM NZ, with $\Delta c/c$= -0.05 and -0.04% and $\Delta a/a$ = -0.03 and -0.02%, respectively, for the typical calcite, and with $\Delta c/c$= -0.03 and -0.03 and $\Delta a/a$ = -0.09 and -0.04%, respectively, for distorted domains.
Therefore, it seems that the lattice contraction is independent of the presence of NZ (at least for 0.11mM NZ), so it is difficult to conclude if, for example, NZ molecules are released from the distorted nanodomains or become occluded in the "new" typical calcite domains after the partial transformation of the distorted nanodomains.
These values are also difficult to interpret because the transformation of the domains coincides with the conversion of vaterite. Therefore, we cannot say much more than that NZ is possibly more associated with the distorted nanodomains, both at the calcite:vaterite and calcite stages.

Line 476: difficult to see why this is included and its relevance to the main story?
It is true that these two parts may not be essential to the main fundamental aspect of the story; however, we believe that at least the screen-printing results, showing the color stability with respect to the pH of the hybrid pigment, are an interesting application that should be reported. Therefore, we decided to focus on this application and removed the increased thermal color stability (see Figure 5b and5c). In addition, this part has been moved after the Discussion and is now named " Potential application of the hybrid pigments" to improve the storyline flow and to make sure it is seen as a potential application only lines 534-565.

Line 495: Spinochrome A (3-acetyl-2,5,7,8-tetrahydroxy-1,4-naphthoquinone), if you put this in full- remind your reader that this is PHNQ- from the intro.
Now line 442. In the revised manuscript, we introduced this molecule in line 77 of the introduction, as suggested, and also reminded the reader in line 442 of the discussion that it is a PHNQ.

Line 518: interactions are strong enough to incorporate a maximum amount of NZ. likely to be?
Now line 467.
We meant that almost all NZ introduced in solution before ACC synthesis, i.e., 0.11mM and 0.53mM is associated with ACC (actual amounts are reported in Table SI2: 0.11mM and 0.51mM). We make this point clearer.

Line 537-538: The transient vaterite and the metastable calcite are not present during sea urchin biomineralization, is this because of analysis issues or really the case?
Now line 528. We don't know if it is due to analysis issues; therefore, it is indeed more accurate to say that "these phases have not been reported," so we modified this sentence accordingly.

Line 541-542: Therefore, we propose that after being released from lavender blue ACC during its dissolution, NZ^{2-}/NZH^{-} molecules capture additional protons from the solution, before getting encapsulated into violet-blue calcite. specify form here – in what form here
Now line 483. Both forms are present at this stage of the reaction, so we replace "/" with "and".

Line 545-546: allowing us to generalize the proposed halochromic ACC crystallization in solution to low water environment as well. not enough is made of this in the MS
We agree that we had only evidence for the color change to purple and access to the protonation state of NZ by DRS characterization during the transformation of ACC into calcite in air, induced by heating. Therefore, we removed these results and the discussion about them, as they would require additional experiments to confirm. This also allows for focusing the storyline on the ACC transformation in water only.

Line 549-550: 100 nm inclusions of NZ enriched with CO_3^{2-} surrounded by a water layer (which one?) as well as nano NZ inclusions likely associated with the metastable calcite phase are present. Make more of the water story

Now line 525-528. The presence of water between NZ and calcite is evidenced by the NMR results, which are now better explained (see answer to comment 2). We actually don't have direct evidence for the presence of a "water layer," so we now write that "the interaction between NZ and calcite is mediated by water molecules." To extend the discussion, we added a reference, Benshir et al. 2013, where the presence of water was also evidenced by NMR, here to stabilize organic inclusions in contact with aragonite.

In addition, the role of water in the formation of the distorted nanodomains is also now discussed lines 492-495.

Line 566: chaperon, chaperone

Now line 520, corrected.

Line 581-582: for applications such as sustainable textile finishing. Yes this is a potential application

We agree, so we ensured that it is presented as such by moving this part to the end of the MS under the name "Potential application of the hybrid pigments".

Line 620: point NZ, point to, the white asterisks, enlarge-not visible

Now line 633. The error has been corrected, and the white asterisks have been replaced by black arrows.

Line 628: CaO3/Ca map; CO3/Ca

Now 643, corrected.

Line 871: sikl, silk

Now line 882, corrected.

Line 874-875: for providing results concerning pure naphthazarin.

Now line 885, "results related to"

Line 877: advices.

Now line 888, advice.

Line 883: beamtimes at

Now line 894 "several beamtimes at the ID15 and ID22 beamlines".

Line 885, 890: acknowledge

Now line 896 and 901, corrected.

Towards the end there are numerous small errors in word endings that need to be corrected.

We ensured that we carefully read and corrected the MS to avoid any errors.

Reviewer #3 (Remarks to the Author):

The authors study the mineralization of calcium carbonate in the presence of the organic pigment naphthazarin. This system forms an interesting bioinspired hybrid material that displays a combination of structural color and pigmentation. It also can shed light on biomineralization process and coloration of sea urchin spines, which consist of polyhydroxy naphthoquinone pigments (PHNQ) within calcium carbonate and can display a range of colors depending on pH. The encapsulation of pigment within the mineral structure protects it from UV and chemical damage. The premise of the work (explained on pg. 4 lines 103-106) is very nice. However, it is extremely difficult to follow the text and to understand what insights the authors provide. I believe the answers are there in the extensive amount of data presented, but the manuscript requires a full overhaul before it can be even reconsidered. These include:

I. General comments

1) reducing the amount of data in the main text to make the text clearer and more linear;

As suggested by reviewer 3, we have substantially revised our manuscript and reorganized the storyline. Therefore, all figures of the main text have been modified accordingly, and the main text rewritten. We ensure that the key findings are presented in the main text, and we have moved all supporting data to the supplementary information file, including UV-visible data in solution. Data about the crystallization of ACC in air induced by heating (TGA/DSC, UV-visible DRS on solid powder) have been removed to focus on the crystallization of ACC in solution and will be used for another manuscript.

2) providing full explanations rather than expecting readers to fill in the blanks, thereby strengthening the arguments;

We carefully attempted to do so, particularly for the NMR results, as both reviewers 2 and 3 had similar remarks. Please see answers to comment 2) of Reviewer 2 and to specific comments 7, 8, 9, and 10 below.

3) including meaningful schematics and more easy-to-process naming conventions to aid understanding and data analysis; and

We now include a scheme for the description of the synthesis (Fig. 1b) and for the screen-printing procedure (Fig. 5b and c), all sample names have been changed as suggested by this reviewer.

4) fixing the plethora of grammatical errors, repetitions, convoluted sentences, and incorrect punctuation that need to be corrected for clarity. The manuscript, in the current state, is nowhere near the quality expected for papers submitted to Nature Communications and has to be rejected.

We ensured that we carefully read and corrected the MS to avoid any grammatical errors, convoluted sentences, and incorrect punctuation. We also request the assistance of a fluent English speaker.

II. Suggestions

1. Include a clarifying schematic in the first figure providing an overview of the system(s) studied.

Fig. 1b illustrates a simplified setup of the direct precipitation method employed in this study for the precipitation of $CaCO_3$ in the presence of naphthazarin, along with the time at which each sample is recovered.

2. Provide easy-to-follow/ easy-to-distinguish names for all material combinations. For example, rather than ACC+0.11mm_NZ, ACC+0.53mM_NZ, Calcite+0.53mM_NZ, why not $ACC_{0.11NZ}$, $Cal_{0.53NZ}$,$Cal_{0.53NZ}$? This would help following the arguments and understanding the figures much smoothly.

As suggested, we simplified sample names and detailed their meaning in a table (Table 1). The text has been modified accordingly.

3. Related to the above, it is difficult to remember/ understand which samples are physical mixtures vs. $CaCO_3$ synthesized in the presence of (and thus incorporating) NZ. Perhaps the following naming convention could be adopted: Calx0.53NZ for a physical mixture of the same composition as $Cal_{0.53NZ}$.

We did it , in addition, the physical mixing of potassium bromide (KBr) with NZ at 0.5wt.% is now named $KBrx_{0.5NZ}$.

4. Include a table listing these short form names of all materials combinations presented in the main text.

We included a table in the main text, Table 1.

5. The naming convention of NZ is likewise difficult to follow and therefore results in ambiguity: for instance, NZ is used to indicate the naphthazarin molecule (with no characteristic ionization, line 147), the protonated species (line 199) and the deprotonated form (line 269), whereas it would aid understanding to consistently use NZ2-, NZ- where relevant. Critically, the ambiguous naming creates confusion in the interpretation of the NMR spectra of Figures 2E, F.

As suggested, we now use the full name “naphthazarin” most of the time when referring to the molecule, without considering its protonation state, and use “NZH_2”, “NZH^-” or “NZ^{2-}” when the protonation state is known or discussed. We use the convention of NZ in the sample names or when considering concentrations [NZ], as suggested above in comment 2).

6. It would be helpful if many of the optical and SEM images could be enlarged and potentially arrows added to more clearly indicate aspects discussed in the text. Likewise, font sizes for text of images, scale bars, axes, legend labels, etc. are often too small.

As suggested by Reviewer 2, font sizes for scale bars have been increased from 8 to 10 pt, and the font size for text in the images has been increased from 10 to 14pt. Additionally, scale bars have been thickened from 2 to 3pt. Axes and legend font sizes of graphs made with Origin Pro software have also been increased.
Optical images and SEM images from Fig. 3 have also been enlarged, and black and white arrows have been added to the SEM images.

7. Figures are often missing key information such as what wt% or concentration of respective compounds was used, etc.

We now display more information about the concentrations. For example, for Fig. 1 and 2, the physical mixture of KBr with [NZ] = 0.5 wt.% has been indicated by renaming the sample as suggested in comment 3. The concentration of NZ for Fig. 2b and d has been specified. If not specified in the figures to avoid overloading, the concentrations are indicated in the Figure legends.

8. Identify the key points and experiments - it is likely that much of what is presented can be moved to the supplementary information.

We paid attention to this and moved all the UV-visible data concerning the reference solutions to the supplementary information, as well as the description of the results regarding the NMR data in solution (Supplementary Fig. 4 and Fig. 5).

9. The Conclusions read like a haphazard collection of sentences. Please revise.

We did revise our conclusions in order to link the different ideas better, please see lines 569-582.

10. Fix the excessive number of grammatical errors throughout the text and figure captions. Individually, some of these are minor, but their prevalence is truly a detriment to the manuscript. See below for some - but absolutely not all - corrections that should be made.
a. Pg 2, line 46: “color stability regarding temperature and pH” → “color stability *with respect to changes in* temperature and pH”
We corrected
b. Pg 3, line 69: “marine life fascinates by” - already not correct.
We changed to “fascinates with”
c. Pg 3, line 77: “such has” → “such as”
We corrected
d. Pg 3, line 96: what is “poly(acrylic)”? poly(acrylic acid) or polyacrylate?
Now line 97, it was poly (acrylic acid) when referring to Xu et al. 2019, Adv. Funct. Mater. 29, 1808146. However, we removed the list of organics to make it more general and cited three reviews that report them instead of the 10 references we had previously.
e. Pg 4, line 122: “1.3%” wt% or ratio? Please clarify.
1.3 was the ratio, this value has been removed from the main text but all concentrations and ratios are reported in the supplementary information in table SI2.
f. Pg 4, line 133: “similar spectra than” → “similar spectra to”
Now line 169, corrected.
g. Pg 4, line 156: “orangish” is much too casual.. “Appear orange-tinted” or “appear to have an orange hue” would be better.
Now line 335, corrected.
More egregious, pg 8, line 246: “purplier” is not an acceptable word here.
This term has been removed
h. Pg 5, line 167: “bleu” → “blue”
This sentence has been removed, but we checked that all “blue are written correctly.
i. Pg 6, line 187: comma (,) between “ACC formation” and “were calculated” is incorrect. This type of comma misuse is seen throughout the manuscript.
This sentence has been modified, but we paid attention throughout the manuscript to use commas properly.
j. Pg 8, line 238: “Similarly than” → “Similarly to”
Now line 229, corrected.
k. Pg 8, line 243: the first “more” should be deleted in “more is more”
Now line 234, corrected.
l. Pg 9, line 277: “Calcite” is capitalized when used in sample names throughout the manuscript but not here.
We changed all sample names as suggested.
m. Pg 17, line 574: “we evidenced” is incorrect.
Now line 570, we changed to “we have demonstrated”
n. Pg 19, line 601: “summarize” → “summarized”

This error has been corrected in the legend of the supplementary file, Figure 4 as it is no longer in the main text.

III. Specific comments

1. Why do the authors choose naphthazarin rather than using the PHNQ directly?

PHNQ are not commercially available, and their extraction and purification in large amounts is quite tedious. Therefore, analogues such as naphthazarin, which is commercially available and relatively inexpensive, are of particular interest for systematic bio-inspired studies, such as the one presented here. This point is mentioned on line 108.

2. Pg 2, lines 44-45: In the abstract, what does it mean that NZ "controls polymorph selection, with minimal impact on ACC formation." In my opinion, this point is neither well-articulated nor is it effectively supported by the text. Please clarify in the abstract and manuscript. Given this phrase, I would expect some discussion on the interactions with specific calcite planes/ explanation of mechanisms, but do not see it in the text.

What we meant by "minimal impact on ACC formation" is that our results show that naphthazarin has no significant effect on ACC particle size, local structure, or stabilization against crystallization in solution. In contrast, naphthazarin appears to control the polymorph selection after ACC crystallization, as it increases the calcite:vaterite ratio and significantly delays the conversion of vaterite into calcite.
We therefore changed the sentence in the abstract to "NZ favors the formation of calcite over vaterite, while having minimal impact on ACC formation" line 44.
In the manuscript, we clarified the minimal impact of NZ on ACC formation, lines 136-138, and the polymorph selection was described lines 141-145 in the results section.
We now have a dedicated discussion section, where we explain the mechanisms of formation of the hybrid pigments. In the discussion, lines 499-502, we now better compare our results to the literature concerning the influence of organics on $CaCO_3$ polymorph selection. Lastly, lines 511-514, we complemented the discussion about the interaction of NZ with the calcite surface by proposing some possible interactions of naphthazarin with specific calcite planes, namely the (012) one.

3. Pg 4, line 129: L*, a*, b*, and sRGB are well-known in the color field, but should be defined somewhere in the manuscript.

As suggested, we did in the Methods section lines 790-792.

4. Pg 7, line 224: "this suggests the formation of Ca^{2+}-NZ in solution" and the entire discussion related to pH/PKa can be explained much clearer and succinctly.

Now lines 185 to 200, we have clarified and shortened this part by also moving the complete description and discussion of these data to the supplementary information lines 59 to 98, as well as the related figure (Supplementary Fig.4), as suggested below.

5. Pg 6, line 187 - pg 7, line 209: The calculations of the pKa1 and pKa2 are perhaps not relevant, since this is already reported in reference 68 and 40 mM of NaCO3 should not change the pKa values. To not dilute the overall message of the manuscript, this and associated figure panels can go to the SI.

As suggested, we have moved this part to the supplementary information (Fig. 4B).

6. More critical, it is written “see experimental” for the pKa calculations, but they are present neither in that section nor in the SI. Also, recommendation to write “see Methods” given that is the title of the relevant section.

The calculation of the pKa values is now described in the Supplementary information file lines 76 to 82.

7. Pg 8, line 264 - Pg 9, line 287: the entire description of NMR measurements and conclusions is sloppy and inconsistent. (see comments 8, 9 for specific examples)

We rewrote the entire paragraph lines 240-276, also taking into account comment 2) of reviewer 2 concerning the “interactions” between calcite, naphthazarin, and water (see answer to comment 2) of reviewer 2 and answers below to specific comments 8 and 9).

8. Pg 8, lines 265 - 268: “The ^{13}C CPMAS NMR spectrum of this sample displays defined peaks at 180, 170 and 168 ppm, one broader signal at 137 ppm and one peak at 117ppm” Looking at Figure 2E, however, it is unclear why the 2 peaks for Calcite+NZ near 170 are distinguished as such while the peaks near 140 ppm are called one “broad peak”? The peak structure in both these locations look very similar and are either doublets or 2 peaks located very close together.

We agree that the “broad peak” at 137 ppm is likely composed of several resonances close together, which is why we named it “a broad signal,” but for clarification, we now use the term “^{13}C resonance” independently of the linewidth of the signal.
In addition, based on the 2D HETCOR, it is clear that the different peaks composing this 137 ppm signal belong to naphthazarin as they all share the same ^{1}H chemical shift (around 3.1 ppm). The same is true for the peak at 170 ppm. In contrast, the peak at 168 ppm is characteristic of CO_3 from calcite, as attested by the pure calcite reference. We now label the ^{13}C NMR spectrum in the revised figure (Fig. 2c) more clearly to avoid confusion, and we provide a better explanation of this point in the revised manuscript version (lines 260-262).

9. Are there negligible NZH2 molecules, as some lines suggest, or enough to make a difference in signal, as indicated elsewhere? Specifically, it is claimed that the Calcite+NZ contains only a few NZH2 molecules. However, the presence of the double peaks near 170 and at 137 ppm (Figure 2E, purple), suggest otherwise, indicating a significant amount of NZH2 (or any secondary species).
Pg. 9, line 303 - Pg. 10 line 307: In conjunction with the explanations of NMR results, the conclusions derived from TGA/DSC measurements also seem tenuous, especially regarding the presence of NZ vs water. Perhaps it is correct, but these aspects must be explained much more clearly and precisely.

After deeper considerations, it is actually difficult to conclude on the nature of the naphthazarin species corresponding to the 170 ppm signal. Indeed, although this value is pretty close to the C=O resonance of the NZH_2 form at 173 ppm, it is important to note that the C-H and C=C resonances of this NZH_2 form, which should be at 135 and 110 ppm, respectively, are not detected in the $Cal_{0.53NZ}$ sample. In contrast, the C-H and C=C signals of the $Cal_{0.53NZ}$ sample match with the NZ^{2} form. Hence, if this 170 ppm peak is unambiguously assigned to Naphthazarin (see the 2D HETCOR spectrum and answer to comment 8), its exact nature is still unknown, but the presence of Naphthazarin in its NZH_2 form can be surely ruled out. Therefore, in the revised version we limit our interpretation to the presence of deprotonated naphthazarin species, and do not mention the residual presence of NZH_2 anymore. The NMR paragraph has been modified accordingly, lines 262-266.

The part of this comment concerning the TGA/DSC measurements is addressed below in response to specific comment 10.

10. Pg 9, line 303 - pg 10, line 305: the evidence provided regarding the presence of water vs NZ is not convincing and not well-supported by the figure.

Figure B, below, presents a direct comparison of the weight losses of pure naphthazarin with those of the ACC_{NZ} samples (Figure B1) and the same comparison for the DSC profiles (Figure B2). They do show that the decomposition of pure naphthazarin starts at 200°C, while adsorbed water typically evaporates below 100°C, and structural water, together with ACC crystallization, occurs between 150 and 220°C. Therefore, it is possible that structural water evaporation and NZ degradation occur simultaneously for $ACC_{0.53NZ}$. However, we decided to remove these data and focus the paper on the crystallization of ACC in solution rather than induced by heating. These data will be part of another manuscript.

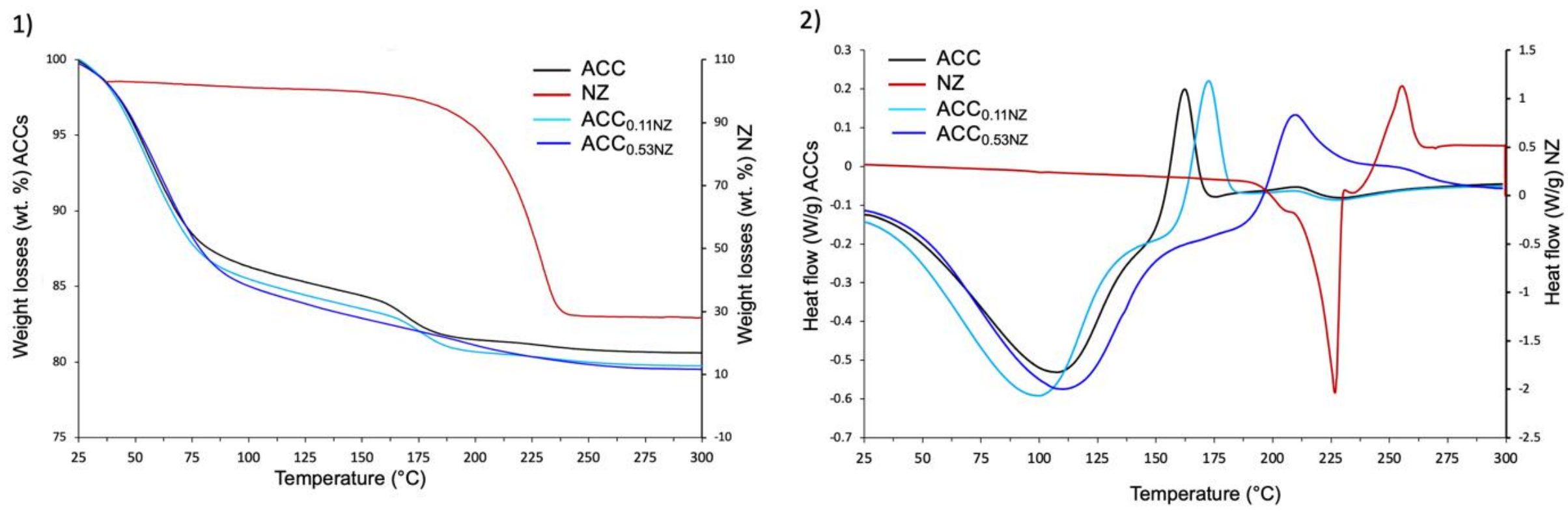

Figure B. 1) TGA profiles and 2) DSC profiles of ACC, NZ, $ACC_{0.11NZ}$ and $ACC_{0.53NZ}$.

11. Pg 10, line 330: text mentions heating to 250degC whereas figure has both 250 and 300. Text should discuss all components of figures.

Now lines 424. Indeed, the (110) splitting disappears at 250°C at the vaterite:calcite stage and at 300°C at the calcite stage. Therefore, we modified the text accordingly.

11. The acknowledgements list a few people who seem to have done key work for this manuscript: David Montero, Arturo J. Alvarez, Isabella Correia, Maeva Chaupard... Do their contributions perhaps warrant authorship (by comparing their contributions to the long list of currently-listed authors).

David Montero and Isabella Correia are the technicians who assisted us during the SEM-FEG and liquid-state NMR analyses performed on external platforms for which we incur costs. These data are not key findings and supportive results. Maeva Chaupard is a student who assisted us with the EELS measurements mentioned in SI, and Arturo Alvarez, a student who also helped with taking four photographs of the screen-printing silk fabrics as part of his training, also reported in SI, so we don't think they warrant authorship. However, we still wanted to acknowledge them. Lastly, because the TGA results were removed, Delphine Talbot, who helped with the TGA measurements, preferred to be removed from the author list of this manuscript and would rather be associated with the follow-up paper on the ACC crystallization in air.

IV. Figure comments

1. Figure 1: add an overview schematic.

We did as suggested.

2. Figure 1A: enlarging the label and scale bar font as well as the material name changes proposed above would be helpful.

As suggested, we did it, and it is now Fig. 1d.

3. Figure 1 B,C: NZ with KBr, NZ in water, ACC, calcite ... what concentrations are used in each case? ACC and calcite are in water?

The spectra of the powder samples, measured in their dried states and previously shown in Figure 1B and C, are now compiled in Fig. 2a. All characteristics of the samples, including NZ concentrations, are reported in Table 1 in the main text, along with their abbreviated names as suggested. ACC and Cal are reference samples synthesized in water in the absence of NZ.

4. Figure 2C: Recommendation to replace colored dots with all-black shapes (e.g. rhombus, circle, square, star, etc.); otherwise the dots are hard to see, and even more so for the large % of colorblind readers.

As suggested for the UV-visible data in solution, Figure 2C has been moved to SI and is now Supplementary Fig. 4C. The colored dots have been replaced by colored shapes. Additionally, we retained the colors because some shapes overlap with each other.

5. Figure 1D,E: the authors claim that the color changes with the focal plane (c.f. Pg. 5, line 163). Regardless of the correctness of the statements, the images in Figure 1D and E seem to be different crystals and not different focal planes of the same crystal.

Indeed, they are different crystals. In Supplementary Fig. 7B-D, we now show two different focal planes of various crystals, which allows us to better distinguish between naphthazarin flakes at the surface of the crystals and the spread blue coloration within the crystals, mainly at the center.

6. Figure 2D, caption: indicate that supernatants are taken as shown in A. It would be nice to see several (~5) additional spectra in the 45 s - 325 s time window as well as at 1 day and 2 days post injection of $CaCl_2$.

Indeed, it would have been interesting to have more measurements. However, in practice, it is not so easy, because the measurement is not automated. Therefore, we must manually stop the synthesis within the Methrom setup and, as soon as possible, filter and centrifuge the supernatant before taking UV-visible measurements. An automated setup would have maybe allowed for more measurements. Here, we instead focus on 5 key times of the reaction and repeat them several times to optimize and validate our approach.

7. Figure 4C: the schematic could be more illuminating. As is, it doesn't provide much insight.

The schematic of Figure 4C, which is now displayed in Fig. 5a is a summary of the ACC transformation showing the different NZ protonation states at the different stages of the reaction and a representation of NZ spatial distribution at the micro and nano-scale. A legend has now been added to distinguish between adsorbed and occluded NZ in order to help the reader. The nanostructure of the bio-inspired colored calcite crystals is also compared to that of sea urchin spines proposed in the literature. This part has also been slightly modified to improve its interpretation: a more straightforward legend and a sea urchin spine (instead of the animal) is added to show which biogenic nanostructure we consider here.

8. Figure SI2 A: Is the wt% of NZ incorrect in the images at the top (should it rather be increasing from left to right)? Is the total mass of material kept constant between the samples measured? Please change the legend colors - there are too many similar colors such that it becomes impossible to tell which spectrum corresponds to which material; consider using dashes or other marking/symbols to better identify the different curves.

The wt.% of NZ mixed with KBr is correct; the lower the amount, the lighter the powder. However, to facilitate easier comprehension, we have inverted the order of the pictures for the different samples and now display them from left to right, with increasing NZ concentrations (now Supplementary Fig. 3A). For sample preparation, the total mass of the different samples is not constant; we used 200 mg of KBr and increased the mass of NZ (as we did for the preparation of $Calx_{NZ}$), which is why we refer to wt.%. We have added this point for KBr physically mixed with NZ as well in the Methods (lines 712). For UV-visible measurements, a similar mass of each sample is put in the sample holder. We changed the legend colors and the sample names as suggested (now Supplementary Fig. 3B).

We would like to thank the reviewers for their additional comments and suggestions, which have been addressed below. The reviewer comments are in black, and our responses are in blue. Changes in the manuscript are also marked in blue.

Reviewer #1 (Remarks to the Author):

The revision has addressed several points raised by this reviewer, and the manuscript has improved substantially through reorganization.

We thank the reviewer for his/her favorable comments.

1) The reason for the remarkable acid stability of the colored textiles, however, remains unresolved, as no control experiments were performed. The additional data (Supplementary Figure 11) also do not clarify this issue, particularly since compositional analyses of the µm-sized patches (e.g., EDX/EDS) are missing. Given that this part of the manuscript is now framed more as an outlook on potential applications of these pigments, this may be acceptable.

As requested by reviewers 2 et 3 who recommended to shorten the manuscript and "completely remove" as well as "publish separately" this section (Reviewer 2), we have removed this part and now only mention the potential application for textile dyeing in the conclusion. Nevertheless, we refer to your decision if this part should be kept in the manuscript and further addressed with additional characterizations.

2) In its present form, the manuscript can be accepted for publication, provided one issue is clarified: the term "halochromic crystallization pathway" suggests the discovery of a novel crystallization mechanism in which halochromy directs crystallization. In reality, the work shows how crystallization conditions influence the halochromic behavior of a dye. The phrasing should therefore be adjusted to avoid overselling, as similar crystallization pathways have been reported previously.
Additional information received on the 3rd of November 2025:
The crystallization route described (solution → amorphous calcium carbonate (ACC) → crystalline $CaCO_3$) is an established pathway and is consistent with classical concepts such as Ostwald's step rule. The authors are surely aware of this established pathway and suitable references (e.g., ref. 32, 34, 39, 47 and more). The halochromic dye functions as a crystallization modifier, influencing nucleation kinetics, ACC stability, morphology, and polymorph selection. Its halochromy is independent of the phase-transition mechanism; neither its presence nor its pH-responsive color change defines a new crystallization pathway. Framing this as a new pathway would oversell the result—indeed, the title currently suggests this by announcing a "halochromic pathway." The novelty lies in using a pH-reporting (halochromic) function within a crystallization modifier, rather than in proposing a distinct route of phase formation.

We particularly thank this reviewer for this comment, with which we fully agree. Therefore, we changed the title and made sure to replace in the abstract and in the text the term "halochromic crystallization pathway" by "halochromic modulation of calcium carbonate crystallization" (line 38, 108, 514). We hope this revision has reached the reviewer expectation. Line 102, we also added "classically" for the different polymorphs formed.

Reviewer #2 (Remarks to the Author):

This is a much improved version of the MS. Referencing much better, figures and legends much better and the front part of the MS hangs together very well. It makes an interesting story to read.

We thank the reviewer for his/her favorable comments.

I. General comments

1) To improve- the abstract needs to be rewritten to remove any ambiguity (the writing for this is much weaker than that for the conclusions, for example).

We have rewritten the abstract to clarify our findings based on the conclusions and hope this has reached the reviewer expectation.

2) I would stop the paper after the presented scheme- conclusions could remain the same. I recommend completely removing the section on dyeing of materials- in my opinion this takes away from the main thrust of the paper. The material should be published separately.

This section has been therefore removed and will be published separately.

II. Specific queries

Line 141 a percentage is not a ratio- please amend
Now line 136, “ratio” has been replaced by “percentage”
Line 378- I think you mean Figure S9 and not as written.
Now line 352, it was meant Fig. S8 F-G, which has been corrected.

Reviewer #3 (Remarks to the Author):

The authors have made substantial changes. Thank you, for example, for explaining the use of NZ vs PHNQs and for using labels for the different materials combinations. Despite the notable effort, I still find this manuscript to be too dense and the conclusions not fully well-reasoned. The authors provide detailed descriptions of their data; however, it is still unclear which pieces of data are most relevant to their story. Especially for Nature Communications, the article ought to be crisp, logical, and profound. I would recommend resubmission after the authors have a chance to thoroughly analyze their data, clarify and substantiate their hypotheses, refine their story, and revamp their figures for clarity. I do appreciate the authors' efforts in revision, but a more serious revision is needed to make the paper suitable for Nat Comm. I hope the following additional comments can be helpful.

We thank the reviewer for considering the efforts we made for clarifying the manuscript. Following all reviewer instructions, we have made new revisions in the whole manuscript to further reduce the amount of data and focus on the most relevant ones (including further data analyses). We hope that this new version has reached the expectation of the reviewer.

I. General comments

1) It seems to me that the main storyline ought to be centered on the authors' findings regarding ".... naphthazarin modifies the kinetic and thermodynamic formation of the crystalline CaCO3 polymorphs…" If this is indeed the case, please explicitly address both the kinetics and thermodynamics and how NZ modifies each of these. As is, this is not clearly and logically drawn out. Several of the references cited in this submission do a great job of distilling similarly complicated data and coherently communicating insights with appropriate messaging and clear, helpful figures. I recommend using those as stylistic guides. One example is ref 43 on ACC formation and transformation (and refs 10-12 therein on the crystallization and the role of inclusions such as magnesium ions, sulfate, etc.). Another example is ref 57 on micelle incorporation into CaCO3 and formation of occlusions.

As highlighted by reviewer 1, the novelty of our study and thus the main story line lies in using "a pH-reporting (halochromic) function within a crystallization modifier, rather than in proposing a distinct route of phase formation". Therefore, in order to avoid any misleading interpretations, we have modified the title and the text accordingly (please see answer to comment 2 of reviewer 1).
The influence of naphthazarin on the kinetic and thermodynamic formation of $CaCO_3$ has been also clarify. In order to do so, we added HR-XRD data at 700 s of the reaction (Fig. 4e.) and further show the influence of NZ on the polymorph selection with time (Fig. 4f.).
In addition, we have drastically implemented Fig. 5, taking inspiration from the references mentioned by the reviewer. The results section and the discussion section have been modified accordingly, respectively lines 385, 400-404 and lines 460-467, 471-480.

2) Response to reviewer 1 regarding crystallography (metastable vs distorted): the authors make unsubstantiated claims and only correct them/ look more deeply when questioned by reviewers. While being open to revising their hypothesis is excellent, this raises a red flag for me. I think they need more time analyzing their data & previous literature to ensure the logic of their conclusions.

Indeed, reviewer 1 comment helped us to refine the interpretation of our HR-XRD results concerning the presence of the shoulder in some calcite peaks. However, our claims and conclusions were correct and remain unchanged (i.e., the shoulder results from the presence of distorted metastable calcite lattice that transforms into typical stable calcite lattice with time and during heating). Nevertheless, the terminology we chose was indeed not appropriate because of the term "phase", therefore, we decided to name the shoulder "distorted calcite" rather than "metastable phase".

As suggested by this reviewer, we took time to re-examine our HR-XRD analyzed data and further analyzed them as well as re-consider previous literature. In particular, considering the different references mentioned above including the one on micelle incorporation (Kim et al. 2011, Nature Materials), we calculated width peaks of the HR-XRD data. After peak fitting, the crystallite sizes of the different nanodomains (stable versus distorted) have been determined as a function of naphthazarin concentration (Fig. 4g). This new data analysis allows for better characterizing the differences between the nanodomains and confirming our conclusion proposing that the distortion is due to the occlusion of impurities (lines 395-400), likely water and that the presence of naphthazarin preferentially modifies the nanostructure of the distorted nanodomains (line 405-407).
Regarding the literature, we reexamined all relevant papers to our study to make sure we are not missing any point of comparison and are placing our mechanism in the landscape of what has been already proposed. For this, we collaborated with Prof. Zou, who is an expert in the stabilization and crystallization mechanism of ACC and is now part of our co-authors. Therefore, we now propose a more detailed mechanism of the different transformations (lines 329-331 and lines 460-465) with a corresponding scheme (new Fig 5).

3) The response to reviewer 1 regarding dissolution of calcium carbonate raises questions regarding the necessity of the NZ/CaCO3 combination. Doesn't the dissolution of CaCO3 discount the authors' claim that "the resulting hybrid pigments exhibit enhanced color stability in response to changes in pH, as demonstrated by textile finishing assays" moot altogether? Rather than acting as a stabilizer for NZ, it seems that CaCO3 is a carrier for delivering NZ to the textile, and it is really the NZ-textile association that stabilizes the NZ.

We agree that NZ is stabilized due to NZ-textile association and that this association is possible thanks to the presence $CaCO_3$, which then acts more as a carrier than a stabilizer. Nevertheless, as requested by reviewer 2 and following the recommendation of this reviewer for lighten the manuscript, this section has been completely removed in the revised version. Nevertheless, we refer to this reviewer's decision and that of reviewer 1 if this part should be kept in the manuscript.

II. Specific comments

4) Thank you for updating the naming convention and including a table. I recommend keeping X#NZ in line with Cal and KBr for the physical mixtures, rather than making it a subscript.

We have followed the reviewer's instruction.

5) Abstract: 'primarily' → 'often' [reason: color can also arise from geometry alone, in the form of layering or patterning of biominerals, i.e. structural color]

Line 35 has been changed as suggested.

6) Intro: 'fascinates with' is technically grammatically correct, but reads unnaturally. OK to keep, of course, but I suggest to replace with something like "Marline life is fascinating for its ability…"

Line 65 has been changed as suggested.

7) Line 92: What is meant by "pigmentation mechanism of biominerals?" Does this refer to the biological mechanisms of molecular pigment incorporation into biominerals, or something else?

Indeed, it refers to what the reviewer is mentioning, so we have modified the sentence line 87-88 to clarify it.

8) Line 153: "color changes" suggest dynamics; I suggest replacing with "color differences." This is relevant to other places within the manuscript as the word choice gives the impression of dynamic color changes as opposed to static color shifts.

As suggested, line 148 has been modified accordingly.

9) Line 203: "indicating that the solution's color can indeed be determined from its spectrum." Seems like a circular statement - when does the spectrum not determine the color?

We agree that this sentence seems a circular statement. Here, we meant that the color can be assessed based on the relative intensity of the NZH_2, NZH^- and NZ^{2-} bands. To make it clearer, we have modified lines 194-199 and hope we have reached the reviewer expectation by doing so.

10) The figures and captions can still be substantially improved - don't make the reader guess what is intended. For example, in Fig 2a, the vertical lines - likely absorption maxima of the corresponding molecule? - are not defined in the caption. The schematic inset in Fig 2b isn't particularly helpful. Fig 3a should say "from top to bottom" rather than the other way around. I am not really sure what to make of Figure 5a. The schematics seem to show the final morphologies than the formation processes.

We have re-examined all figures and legends:
-Fig. 2a: vertical lines have been changed to colored vertical bars and their signification mentioned in the legend (line 1021-1022).
-Fig 2b: the inset has been removed; in order to keep the same color code as in Fig2a, the color code for the spectra has been changed from a dark blue line to a dark blue dashed line for 45 s (corresponding to $ACC_{0.53NZ}$ retrieval) and from a purple line to a dark blue line for 3 d (corresponding to $Cal_{0.53NZ}$ retrieval). A sentence has been added in the legend to specify the correspondence between time points of the reaction and sample retrievals (line 1024-1025).
-Fig 2c and 2d: the NMR spectra from the $Cal_{0.53NZ}$ sample have then also been changed from purple to dark blue.
-Fig 3a: we have corrected the legend.
-Fig 5 has been drastically modified to propose a detailed formation process for the different steps of the reaction rather than the final morphologies only (see also the answer to the general comment 2) of this reviewer).

11) Line 378: should be "Fig S8"?

Indeed, it should be Fig. S8 F-G, so it has been corrected, now line 352.

12) Lines 367-372: Regarding the formation of pores, the authors suggest that stacking of naphthazarin could result in pore formation but also that this could be a result of pitting/etching due to acid/base conditions; have the authors run a control experiment that can clarify this?

A control experiment has been previously reported in the literature, which we cited: ref. 55. It shows that pitting (rhombohedral holes) can be observed on pure calcite grown at pH 8.6. Here, our control experiment (without naphthazarin) show that calcite crystals synthesized at pH 9.2 (Fig 1c) present smooth surfaces with no pits nor "crevasses" (Fig 3c'). Since the final pH of the synthesis medium with naphthazarin is also 8.6, we propose that the rhombohedral holes can result from a pH effect. We clarify that crevasses may be due to NZ stacking and pits to acid/base conditions (line 346 and 347).

Does the growth of these pores over time result in any structural colour?

We did not observe any structural colour of the crystals by light microscopy. Pores would need to present a highly organized repeated structure at the nm scale in order to do so, which appear more randomly distributed here.

Additionally, the discussion around the SEM, optical images and EELS used for pore analysis could be more succinct and less disjoint.

We agree and therefore have reduced the discussion about these data and also combined the different discussion about the pores. In particular, we have moved the description of the internal pores observed in the FIB lamella after the SEM section reporting (line 351-357).

We would like to deeply thank the three reviewers for their implications, concerns and insightful comments all along the reviewing process that greatly contributed to the quality of the paper. The comments are reported below and do not seem to need further answer.

REVIEWERS' COMMENTS

Reviewer #1 (Remarks to the Author):

The revised manuscript sufficiently addresses all remaining issues raised by this reviewer who now can recommend acceptance of the manuscript for publication.

Reviewer #2 (Remarks to the Author):

In addition to previous comments (I will not repeat them here) I am happy that the authors have addressed this referees' queries. The new MS is significantly improved. the story is clear and well supported by a range of relevant data. The topic is of interest to the biomineralization community as well as to those interested in 'colour'! I believe it is now suitable for publication.

Reviewer #3 (Remarks to the Author):

The authors have substantially improved the manuscript through reorganization and curation of the results, clarifying their explanations, and revising the figures. Fig. 5 schematic is much more comprehensible and informative now. I believe this manuscript is now ready for publication in Nature Communications.

The authors study the mineralization of calcium carbonate in the presence of the organic pigment naphthazarin. This system forms an interesting bioinspired hybrid material that displays a combination of structural color and pigmentation. It also can shed light on biomineralization process and coloration of sea urchin spines, which consist of polyhydroxy naphthoquinone pigments (PHNQ) within calcium carbonate and can display a range of colors depending on pH. The encapsulation of pigment within the mineral structure protects it from UV and chemical damage.

The premise of the work (explained on pg. 4 lines 103-106) is very nice. However, it is extremely difficult to follow the text and to understand what insights the authors provide. I believe the answers are there in the extensive amount of data presented, but the manuscript requires a full overhaul before it can be even reconsidered. These include: 1) reducing the amount of data in the main text to make the text clearer and more linear; 2) providing full explanations rather than expecting readers to fill in the blanks, thereby strengthening the arguments; 3) including meaningful schematics and more easy-to-process naming conventions to aid understanding and data analysis; and 4) fixing the plethora of grammatical errors, repetitions, convoluted sentences, and incorrect punctuation that need to be corrected for clarity. The manuscript, in the current state, is nowhere near the quality expected for papers submitted to Nature Communications and has to be rejected.
More detailed comments and suggestions are below.

Suggestions:

1. Include a clarifying schematic in the first figure providing an overview of the system(s) studied.
2. Provide easy-to-follow/ easy-to-distinguish names for all material combinations. For example, rather than ACC+0.11mm_NZ, ACC+0.53mM_NZ, Calcite+0.53mM_NZ, why not $ACC_{0.11NZ}$, $Cal_{0.53NZ}$,$Cal_{0.53NZ}$? This would help following the arguments and understanding the figures much smoother.
3. Related to the above, it is difficult to remember/ understand which samples are physical mixtures vs. $CaCO_3$ synthesized in the presence of (and thus incorporating) NZ. Perhaps the following naming convention could be adopted: Calx0.53NZ for a physical mixture of the same composition as $Cal_{0.53NZ}$.
4. Include a table listing these short form names of all materials combinations presented in the main text.
5. The naming convention of NZ is likewise difficult to follow and therefore results in ambiguity: for instance, NZ is used to indicate the naphthazarin molecule (with no characteristic ionization, line 147), the protonated species (line 199) and the deprotonated form (line 269), whereas it would aid understanding to consistently use NZ2-, NZ- where relevant. Critically, the ambiguous naming creates confusion in the interpretation of the NMR spectra of Figures 2E, F.
6. It would be helpful if many of the optical and SEM images could be enlarged and potentially arrows added to more clearly indicate aspects discussed in the text. Likewise, font sizes for text of images, scale bars, axes, legend labels, etc. are often too small.

7. Figures are often missing key information such as what wt% or concentration of respective compounds was used, etc.
8. Identify the key points and experiments - it is likely that much of what is presented can be moved to the supplementary information.
9. The Conclusions read like a haphazard collection of sentences. Please revise.
10. Fix the excessive number of grammatical errors throughout the text and figure captions. Individually, some of these are minor, but their prevalence is truly a detriment to the manuscript. See below for some - but absolutely not all - corrections that should be made.
    a. Pg 2, line 46: “color stability regarding temperature and pH” → “color stability *with respect to changes in* temperature and pH”
    b. Pg 3, line 69: “marine life fascinates by” - already not correct.
    c. Pg 3, line 77: “such has” → “such as”
    d. Pg 3, line 96: what is “poly(acrylic)”? poly(acrylic acid) or polyacrylate?
    e. Pg 4, line 122: “1.3%” wt% or ratio? Please clarify.
    f. Pg 4, line 133: “similar spectra than” → “similar spectra to”
    g. Pg 4, line 156: “orangish” is much too casual.. “Appear orange-tinted” or “appear to have an orange hue” would be better. More egregious, pg 8, line 246: “purplier” is not an acceptable word here.
    h. Pg 5, line 167: “bleu” → “blue”
    i. Pg 6, line 187: comma (,) between “ACC formation” and “were calculated” is incorrect. This type of comma misuse is seen throughout the manuscript.
    j. Pg 8, line 238: “Similarly than” → “Similarly to”
    k. Pg 8, line 243: the first “more” should be deleted in “more is more”
    l. Pg 9, line 277: “Calcite” is capitalized when used in sample names throughout the manuscript but not here.
    m. Pg 17, line 574: “we evidenced” is incorrect.
    n. Pg 19, line 601: “summarize” → “summarized”

Specific comments:

1. Why do the authors choose naphthazarin rather than using the PHNQ directly?
2. Pg 2, lines 44-45: In the abstract, what does it mean that NZ “controls polymorph selection, with minimal impact on ACC formation.” In my opinion, this point is neither well-articulated nor is it effectively supported by the text. Please clarify in the abstract and manuscript. Given this phrase, I would expect some discussion on the interactions with specific calcite planes/ explanation of mechanisms, but do not see it in the text.
3. Pg 4, line 129: L*, a*, b*, and sRGB are well-known in the color field, but should be defined somewhere in the manuscript.
4. Pg 7, line 224: “this suggests the formation of Ca^{2+}-NZ in solution” and the entire discussion related to pH/PKa can be explained much clearer and succinctly.
5. Pg 6, line 187 - pg 7, line 209: The calculations of the pKa1 and pKa2 are perhaps not relevant, since this is already reported in reference 68 and 40 mM of NaCO3 should not change the pKa values. To not dilute the overall message of the manuscript, this and associated figure panels can go to the SI.
6. More critical, it is written “see experimental” for the pKa calculations, but they are present neither in that section nor in the SI. Also, recommendation to write “see Methods” given that is the title of the relevant section.

7. Pg 8, line 264 - Pg 9, line 287: the entire description of NMR measurements and conclusions is sloppy and inconsistent. (see comments 8, 9 for specific examples)
8. Pg 8, lines 265 - 268: “The ^{13}C CPMAS NMR spectrum of this sample displays defined peaks at 180, 170 and 168 ppm, one broader signal at 137 ppm and one peak at 117ppm” Looking at Figure 2E, however, it is unclear why the 2 peaks for Calcite+NZ near 170 are distinguished as such while the peaks near 140 ppm are called one “broad peak”? The peak structure in both these locations look very similar and are either doublets or 2 peaks located very close together.
9. Are there negligible NZH2 molecules, as some lines suggest, or enough to make a difference in signal, as indicated elsewhere? Specifically, it is claimed that the Calcite+NZ contains only a few NZH2 molecules. However, the presence of the double peaks near 170 and at 137 ppm (Figure 2E, purple), suggest otherwise, indicating a significant amount of NZH2 (or any secondary species). Pg. 9, line 303 - Pg. 10 line 307: in conjunction with the explanations of NMR results, the conclusions derived from TGA/DSC measurements also seem tenuous, especially regarding the presence of NZ vs water. Perhaps it is correct, but these aspects must be explained much more clearly and precisely.
10. Pg 9, line 303 - pg 10, line 305: the evidence provided regarding the presence of water vs NZ is not convincing and not well-supported by the figure.
11. Pg 10, line 330: text mentions heating to 250degC whereas figure has both 250 and 300. Text should discuss all components of figures.
12. The acknowledgements list a few people who seem to have done key work for this manuscript: David Montero, Arturo J. Alvarez, Isabella Correia, Maeva Chaupard… Do their contributions perhaps warrant authorship (by comparing their contributions to the long list of currently-listed authors).

Figure comments:

1. Figure 1: add an overview schematic.
2. Figure 1A: enlarging the label and scale bar font as well as the material name changes proposed above would be helpful.
3. Figure 1 B,C: NZ with KBr, NZ in water, ACC, calcite … what concentrations are used in each case? ACC and calcite are in water?
4. Figure 2C: Recommendation to replace colored dots with all-black shapes (e.g. rhombus, circle, square, star, etc.); otherwise the dots are hard to see, and even more so for the large % of colorblind readers.
5. Figure 1D,E: the authors claim that the color changes with the focal plane (c.f. Pg. 5, line 163). Regardless of the correctness of the statements, the images in Figure 1D and E seem to be different crystals and not different focal planes of the same crystal.
6. Figure 2D, caption: indicate that supernatants are taken as shown in A. It would be nice to see several (~5) additional spectra in the 45 s - 325 s time window as well as at 1 day and 2 days post injection of $CaCl_2$.
7. Figure 4C: the schematic could be more illuminating. As is, it doesn’t provide much insight.

8. Figure SI2 A: Is the wt% of NZ incorrect in the images at the top (should it rather be increasing from left to right)? Is the total mass of material kept constant between the samples measured? Please change the legend colors - there are too many similar colors such that it becomes impossible to tell which spectrum corresponds to which material; consider using dashes or other marking/symbols to better identify the different curves.

These comments are not exhaustive, and I strongly recommend that the authors reevaluate the presentation of information and do a thorough revision of the entire manuscript.
